# Supplementary material for: Approaches for combining primary care electronic health record data from multiple sources: a systematic review of observational studies
Source: BMJ Open. 2020 Oct 14;10(10):e037405. doi: 10.1136/bmjopen-2020-037405 (PMC7559041; doi:10.1136/bmjopen-2020-037405)
Supplement: Supplementary data [file bmjopen-2020-037405supp001.pdf]

BMJ Publishing Group Limited (BMJ) disclaims all liability and responsibility arising from any reliance placed on this supplemental material which has been supplied by the author(s)

Multidatabase Systematic Review: Supplementary Appendix

Appendix S1: Review Protocol: Approaches for combining primary care EHR data from multiple sources: a systematic review of observational studies

Appendix S1: Review Protocol: Approaches for combining primary care EHR data from multiple sources: a systematic review of observational studies

Contents

1 Introduction 2
2 Rationale and scope 2
2.1 Objectives 2
3 Methods 2
3.1 Eligibility Criteria 2
3.2 Information sources 3
3.3 Search strategy 3
3.3.1 Test sets 3
3.3.2 Conceptual searches 3
3.3.3 Search of named databases and common terms 4
3.4 Study records 4
3.4.1 Data Management 4
3.4.2 Selection process 4
3.4.3 Data extraction 5
4 Data synthesis 6
5 References 7
6 Appendices 8
6.1 Appendix I: OVID Medline Search Strategy for Systematic Review 8
6.2 Appendix 2: Data extraction tables 10

Dedman D, *et al. BMJ Open* 2020; 10:e037405. doi: 10.1136/bmjopen-2020-037405

1 Introduction

A recent systematic review evaluated methods for data management and analysis of multi-database studies in pharmacoepidemiology. The current study adopted a similar approach, but with a specific focus on studies combining primary care EHR databases, while expanding the scope to include all areas of observational epidemiology and healthcare database research.

2 Rationale and scope

The primary aim was to describe the full range of completed studies which brought together primary care electronic health record (EHR) data from two or more sources, and to generate a clear overview of methods used to manage, assess variability in, and analyse the data. The main motivation for the review was to inform a planned study of cancer risk in patients with Huntington's disease combining data from two UK primary care EHR databases.

The review specifically covered 'horizontal' combination of data from different sources, containing data from different sets of individuals (possibly after deduplication). Here the primary purpose of combining might be to increase the number of individuals available for analysis, or increase the range of population settings to which findings can be applied (i.e. increase external validity). The review did not include studies where data sources were only combined 'vertically' i.e. linkage studies whereby data on the same individuals was combined to provide richer (deeper) information about each study participant.

As the focus was on analysis of primary care EHR data, the review was restricted to studies using primary care EHR data from at least two sources. For the purpose of this review, primary care EHR data was defined as data collected by primary care clinicians and related staff for the purpose of diagnosis, treatment, management, and delivery of care of individual patients. It may include information collected or contributed by other care providers (1). It excludes data generated primarily for administrative purposes such as claims data.

The review was conducted following the PRISMA guidelines for reporting in systematic reviews (2,3) [http://www.prisma-statement.org].

2.1 Objectives

1. Identify studies which combined data from two or more sources of primary care EHR data.
2. Summarise key study characteristics, including the main reasons or motivations for combining data from different EHR databases
3. Describe the methods used to manage and analyse data including, where applicable, methods for combining data.
4. Describe the methods used to assess and report heterogeneity between primary care EHR data sources.
5. Describe and summarise any reported differences between different primary care EHR data sources.

Quality and completeness of reporting of methods was assessed using criteria adapted from the STROBE (4) and RECORD (5) guidelines. No formal assessment of quality in terms of risk of bias was attempted, either for individual studies, or for particular methodological approaches.

3 Methods

3.1 Eligibility Criteria

1. Peer reviewed, English language publication of an observational study.

2. Study participants selected from at least *two* different primary care EHR data sources.
3. Re-analyses of previously reported cohorts were included if they used substantially different methods.

There were no specific eligibility criteria relating to exposures, comparator groups, outcomes, or study design.

3.2 Information sources

The following databases were searched for eligible studies

1. Medline (OVID)
2. EMBASE (OVID)

3.3 Search strategy

The key challenges anticipated when searching for relevant studies were the lack of a specific MeSH concept for multi-database studies (6), and the lack of consensus on terminology for such studies in the published literature. This raised the possibility of having to hand search all database studies.

3.3.1 Test sets

Given the challenges outlined above, the performance of different search strategies was evaluated for their ability to recall results from the following test references sets:

> Test Set 1: 1673 publications using CPRD or GPRD databases were identified using keyword searches in Medline.
>
> Test Set 2: an *ad hoc* sample of 14 records identified from a published systematic review of multi-database pharmacoepidemiology studies (6), plus a small number identified from non-systematic review of the literature. All of these studies used at least one primary care EHR data source.

3.3.2 Conceptual searches

An initial search strategy was defined based on 3 concepts, identified using both MeSH terms and keywords, and the sensitivity of each concept was assessed against Test Sets 1 and 2 :

1. Database studies: this included MeSH terms and keyword searches for databases and related concepts such as Electronic Health Records, and Computerized Medical Records System. This concept was reasonably sensitive for recalling CPRD/GPRD studies (Test Set 1 sensitivity= 1365/1673 = 0.82), and all records in the sample of multi-database studies (Test Set 2 sensitivity 14/14 = 1). However it returned over 395 thousand results.
2. Primary care setting: a combined MeSH term and keyword search returned over 280 thousand records, but had very low sensitivity with both Test Set 1 (526/1673 = 0.31), and only moderate sensitivity with Test Set 2 (10/14 = 0.71).
3. (Observational) epidemiology studies: The InterTASC Information Specialists' Sub-Group Search Filter Resource (7) was accessed to identify potentially suitable and validated search filters. Waffenschmidt *et al* (8) reviewed search strategies to identify epidemiological studies, concluding that there was "no suitable approach to conducting *efficient* systematic searches for epidemiologic publications in bibliographic databases". One filter, from a systematic review of Hepatitis C prevalence in prisons by Larney *et al* (9), was found to be suitably sensitive, recalling almost 96% of their test set of 729 references. This filter had very high sensitivity for CPRD/GPRD studies in Test Set 1 (1593/1673 = 0.95), and also recalled all records in Test Set 2 (sensitivity 14/14 = 1). However it returned over 6 million records.

Dedman D, *et al. BMJ Open* 2020; 10:e037405. doi: 10.1136/bmjopen-2020-037405

A Medline search combining all 3 of the concepts above returned 14309 records and recalled only 10 of the 14 records in Test Set 2 (sensitivity 0.71), with the sensitivity being limited by the 'Primary Care' concept. However any attempt to broaden this concept to improve sensitivity would have increased the number of recalled records beyond what could be feasibly reviewed manually. For example, broadening the Primary Care concept to include 'population-based' studies, allowed recall of 12/14 records from Test Set 2, but increased the total number of records retrieved to 23656.

3.3.3 Search of named databases and common terms

Given the relatively poor performance of the conceptual searches, an alternative strategy was developed using a combination of named databases, and commonly used key words and phrases. Three sources were used to compile a list of candidate primary care EHR (or closely related) databases:

1. ENCePP Resources Database: the European Network of Centres for Pharmacoepidemiology and Pharmacovigilance (ENCePP) maintains a searchable database of research organisations, networks and data sources (10). Despite a strong European focus, the register is not restricted to European data sources. The database was searched to identify 20 registered data sources identified as a 'Routine primary care electronic patient registry'.
2. B.R.I.D.G.E. TO DATA®: a "non-profit online reference describing population healthcare databases for use in epidemiology and health outcomes research" (10). A search interface is provided as a subscription service, however a simple listing of 286 named database resources was downloaded (23 Jan 2018) and searched for candidate primary care databases.
3. A list of 41 databases or primary care research networks identified in a 2017 review by Gentil et al (11) which examined factors associated with successful implementation of initiatives to collect and curate collections of primary care electronic health record data.

Abstracts of published studies identified from the initial named database search were scanned for additional terms and phrases used to describe the primary care EHR data sources, and these were added to the final search. Finally, reference lists of papers selected for full review were searched for additional studies.

The full search strategy used to search the Medline database is included in Appendix I.

This search was able to recall 12/14 records in Test Set 2 (sensitivity = 0.86), and all records in Test Set 1 (by definition - since CPRD/GPRD were among the named databases included in the search).

3.4 Study records

3.4.1 Data Management

All search results were exported from OVID in batches, with copies of export files retained. The references were imported into Mendeley V1.1 (Mendeley, Elsevier, Amsterdam, NL). Details of studies selected for full review were exported into a Microsoft Access database, in which was used to record subsequent inclusion/exclusion decisions and data extraction.

3.4.2 Selection process

Initial screening of all selected titles and abstracts was undertaken by 1 reviewer (DD). A second reviewer (MC) screened a 20% random sample of all abstracts. Full text was reviewed in instances where it was not possible to assess eligibility from the title and abstract alone.

Full text was then obtained for all papers selected during the initial abstract screening, and read by two reviewers, who completed the eligibility assessment before and performing data extraction.

3.4.3 Data extraction

Each reviewer extracted standardised information via a data collection form into a review database (MS Access). The following information was collected:

Year of publication
Primary care EHR data source details:<br>• number of sources<br>• name(s) of database<br>• country
Other (non-primary care EHR) data source details:<br>• number of sources<br>• name(s) of database<br>• type of database e.g. claims, disease registry<br>• country
Study type or broad objective e.g.:<br>• Descriptive e.g. drug utilisation, disease epidemiology<br>• Comparative or hypothesis testing e.g. comparative treatment effectiveness, drug safety, disease epidemiology<br>• Disease risk prediction<br>• Methodology / data quality assessment<br>• Health service research
Study design e.g.:<br>• Cross sectional<br>• Case-control<br>• Cohort<br>• Case-only designs<br>• Time series
Target population(s) for study:
Main exposure(s) if applicable e.g.:<br>• Drug treatment<br>• Disease risk factor<br>• Other
Main outcome(s) if applicable e.g.:<br>• All cause mortality<br>• Disease<br>• Treatment patterns<br>• Other
Main analysis methods, including confounder control e.g.:<br>• descriptive using summary statistics<br>• incidence or prevalence calculations<br>• multiple regression modelling
Motivation or rationale for using and/or combining data sources e.g.:<br>• increase study power<br>• assess consistency of findings in multiple settings<br>• international comparisons
Assssement of heterogeneity of exposures, outcomes and effect estimates e.g.:<br>• descriptive only (no formal comparisons)<br>• univariate comparisons<br>• formal tests for heterogeneity (Q-test, I-test)
Main approach for combining data sources e.g.:<br>• Data not combined: results presented separately for each source<br>• Meta-analysis of aggregate results from each data source

• Meta-analysis of semi-aggregated results from each data source<br>• Pooled analysis of individual patient data
Data management and analysis e.g.:<br>• Data managed and analysed separately by each database partner<br>• Use of common protocol<br>• Use of common data model (study specific, or externally defined e.g. OMOP CDM)<br>• Use of common analysis programs<br>• Data management and analysis arrangements (distributed, central, hybrid)<br>• Data sharing model (individual, semi-aggregate, aggregate)

4 Data synthesis

Results will be summarised in tables which will describe .

- basic characteristics of included studies: study design, statistical method
- rationale for combining databases
- methods used to assess heterogeneity
- methods use for combining or synthesising results

Further narrative descriptions will focus on specific subgroups. For example analytical studies which combined two or more databases from the same country.

5 References

1. Häyrinen K, Saranto K, Nykänen P. Definition, structure, content, use and impacts of electronic health records: a review of the research literature. Int J Med Inform [Internet]. 2008 May;77(5):291–304. Available from: http://www.ncbi.nlm.nih.gov/pubmed/17951106

2. Liberati A, Altman DG, Tetzlaff J, Mulrow C, Gøtzsche PC, Ioannidis JPA, et al. The PRISMA Statement for Reporting Systematic Reviews and Meta-Analyses of Studies That Evaluate Health Care Interventions: Explanation and Elaboration. PLoS Med [Internet]. 2009 Jul 21;6(7):e1000100. Available from: http://dx.plos.org/10.1371/journal.pmed.1000100

3. Moher D, Liberati A, Tetzlaff J, Altman DG, PRISMA Group. Preferred reporting items for systematic reviews and meta-analyses: the PRISMA statement. Ann Intern Med [Internet]. 2009 Aug 18;151(4):264–9, W64. Available from: http://www.ncbi.nlm.nih.gov/pubmed/19622511

4. von Elm E, Altman DG, Egger M, Pocock SJ, Gøtzsche PC, Vandenbroucke JP, et al. The Strengthening the Reporting of Observational Studies in Epidemiology (STROBE) statement: guidelines for reporting observational studies. J Clin Epidemiol [Internet]. 2008 Apr;61(4):344–9. Available from: http://www.ncbi.nlm.nih.gov/pubmed/18313558

5. Benchimol EI, Smeeth L, Guttmann A, Harron K, Moher D, Petersen I, et al. The REporting of studies Conducted using Observational Routinely-collected health Data (RECORD) statement. PLoS Med [Internet]. 2015 Oct;12(10):e1001885. Available from: http://www.ncbi.nlm.nih.gov/pubmed/26440803

6. Bazelier MT, Eriksson I, de Vries F, Schmidt MK, Raitanen J, Haukka J, et al. Data management and data analysis techniques in pharmacoepidemiological studies using a pre-planned multi-database approach: a systematic literature review. Pharmacoepidemiol Drug Saf [Internet]. 2015 Sep [cited 2017 Feb 15];24(9):897–905. Available from: http://doi.wiley.com/10.1002/pds.3828

7. Glanville J, Lefebvre C, Wright K. ISSG Search Filter Resource [Internet] [Internet]. York (UK): The InterTASC Information Specialists' Sub-Group; 2008. 2008 [cited 2018 Mar 12]. Available from: https://sites.google.com/a/york.ac.uk/issg-search-filters-resource/how-to-cite-this-site

8. Waffenschmidt S, Hermanns T, Gerber-Grote A, Mostardt S. No suitable precise or optimized epidemiologic search filters were available for bibliographic databases. J Clin Epidemiol [Internet]. 2017 Feb 1 [cited 2017 Oct 17];82:112–8. Available from: http://www.sciencedirect.com/science/article/pii/S0895435616303663

9. Larney S, Kopinski H, Beckwith CG, Zaller ND, Jarlais D Des, Hagan H, et al. Incidence and prevalence of hepatitis C in prisons and other closed settings: Results of a systematic review and meta-analysis. Hepatology [Internet]. 2013 Oct [cited 2017 Oct 17];58(4):1215–24. Available from: http://www.ncbi.nlm.nih.gov/pubmed/23504650

10. ENCePP. ENCePP Resource Database [Internet]. [cited 2018 Jan 18]. Available from: http://www.encepp.eu/encepp/resourcesDatabase.jsp

11. Gentil M-L, Cuggia M, Fiquet L, Hagenbourger C, Le Berre T, Banâtre A, et al. Factors influencing the development of primary care data collection projects from electronic health records: a systematic review of the literature. BMC Med Inform Decis Mak [Internet]. 2017 Dec 25 [cited 2017 Oct 24];17(1):139. Available from: http://bmcmedinformdecismak.biomedcentral.com/articles/10.1186/s12911-017-0538-x

6 Appendices

6.1 Appendix I: OVID Medline Search Strategy for Systematic Review

Run on 16 Feb 2018 on Medline to Feb Week 2 2018

Item	Term
1	Lifelink.mp.
2	Disease Analyzer.mp.
3	(OsMed not dysplasia).mp.
4	EpiChron.mp.
5	(Integrated Primary Care Information or IPCI or Interdisciplinary Processing of Clinical Information).mp.
6	PHARMO.mp.
7	(Primary Care Clinical Informatics Unit or PCCIU).mp.
8	(BIFAP or Database for Pharmacoepidemiolog* Research in Primary Care or Base de datos para la Investigacion Farmacoepidemiologica en Atencion Primaria).mp.
9	(SIDIAP or Information System for the Development of Research in Primary Care or (Sistema and Desenvolupament and Atencio Primaria)).mp.
10	((LINH and database) or Netherlands Information Network of General Practice or Landelijk Informatie Netwerk Huisatsenzorg).mp.
11	(NIVEL adj3 database).mp.
12	(CPRD or Clinical Practice Research Data*).mp.
13	(GPRD or General Practice Research Data*).mp.
14	(OPCRD or Optimum Patient Care Research Data*).mp.
15	((THIN adj4 database) or Health Information Network or Health Improvement Network).mp.
16	(QResearch or Q Research).mp.
17	(ResearchOne or (Research One adj4 database*)).mp.
18	(DIN LINK or (DIN adj4 database*) or Doctors Independent Network).mp.
19	((SAIL adj4 Data*) or Secure Anon* Information Link*).mp.
20	(Arianna data* or (Caserta and database)).mp.
21	Pedianet.mp.
22	(Health Search and (Database or Dataset)).mp.
23	Longitudinal Patient Database.mp.
24	(mediplus and database).mp.
25	(centricity and (database* or EMR or electronic medical record*)).mp.
26	OCHIN.mp.
27	PHINEX.mp.
28	Regenstrief Medical Record.mp.
29	(Clalit and database).mp.
30	(Electronic Medical Record Administrative data Linked Database or EMRALD).mp.
31	(Intego or (database* and (general practice or primary care) and Belgi*)).mp.
32	Julius General Practi*.mp.
33	((primary care or primary health care or general practi* or family practi* or ambulatory care) adj4 database*).mp.
34	population database*.mp
35	(healthcare adj2 database*).mp
36	health care database*.mp
37	(electronic health* adj2 database*).mp

38	(population health* adj2 database*).mp
39	((EHR or electronic health record*) adj2 database*).mp
40	Or/1-39
41	limit 40 to (abstracts and english language and yr="2000 -Current")

6.2 Appendix 2: Data extraction tables

Table name: StudyInfo1

Description: Basic publication details

Completed for: All studies selected at initial screening round

Name	Type	Size	Description
StudyID	Long Integer	4	UNIQID for study
Authors	Long Text	-	
Title	Long Text	-	
JName	Short Text	255	Name of journal
JVol	Short Text	255	Journal volume
JPage	Short Text	255	Journal pages
YearPub	Long Integer	4	Year of publication

Table name: DataSource1

Description: Key information about each primary care EHR database

Completed for: Each primary care EHR database, plus partial details collected for other databases described in the included studies

Name	Type	Size	Description
DataSourceID	Long Integer	4	UNIQID for datasource
SourceName	Short Text	255	Full or official name of database
Shortname	Short Text	255	Short name for database
Aliases	Short Text	255	Other names used in published papers
IsEHR	Short Text	10	Is it a primary care EHR database
SourceType	Integer	2	What type of database (primary care EHR or some other type)
SourceCountry	Short Text	255	Country of database
ClinicalCoding	Short Text	255	Name of clinical coding scheme (if known)
DrugCoding	Short Text	255	Name of drug coding scheme (if known)
SourceInfo1	Long Text	-	Other relevant information about data source
SourceReference	Long Text	-	Key reference for data source

Table name: Review1

Description: Summary of review process, including whether publication was selected for full review

Completed for: All studies selected at initial screening round

Name	Type	Size	Description
Review1ID	Long Integer	4	record identifier
StudyID	Long Integer	4	ID number of paper being reviewed
ReviewerID	Integer	2	Reviewer: DD or MC (or adjudicated)
IncExc	Integer	2	Inclusion / exclusion with reasons
IncExcComment 1	Long Text	-	Comment on decision to include or exclude
Review1Date	Date With Time	8	Date of completion of review

Table name: Review2

Description: Full details of study objectives, methods and relevant results

Completed for: All studies included after full paper review

Name	Type	Size	Description
Review2ID	Long Integer	4	record identifier
StudyID	Integer	2	Study ID (FK)
ReviewerID	Short Text	20	Reviewer: DD or MC (or Adjudicated)
Review2Status	Short Text	255	set to "in progress" once data entry is started; user specifies when completed
Review2Date	Date With Time	8	autoset when status is set to completed
Objective	Short Text	50	Short description of study objectives
OBjectiveText	Long Text	-	Further details of study objectives, including quoted text from publication if relevant
TargetPop	Short Text	255	Short description of the target population or patient group for the study
TargetPopText	Long Text	-	Further details of target population, including quoted text from publication if relevant
MainExposures	Short Text	255	Lookup: category for main exposure(s)
MainExposuresText	Long Text	-	Further details of main exposure(s), including quoted text from publication if relevant
MainOutcomes	Short Text	255	Lookup: category for main outcome(s)
MainOutcomesText	Long Text	-	Further details of main outcome(s), including quoted text from publication if relevant
StudyType	Short Text	255	Lookup: type of study e.g drug safety; disease epidemiology;
StudyTypeText	Long Text	-	Further details of study type, including quoted text from publication if relevant
StudyDesign	Short Text	255	Lookup: study design e.g. cohort, case control, etc
StudyDesignText	Long Text	-	Further details of study design, including quoted text from publication if relevant
MainRationale	Short Text	255	Lookup: main reason for combining data from multiple sources
MainRationaleText	Long Text	-	Further details of study rationale, including quoted text from publication if relevant
Stats1	Short Text	255	Lookup: main statistical method or model used
Stats1Text	Long Text	-	Further details of statistical method or model including quoted text from publication if relevant
ExposureTime	Short Text	255	Lookup: main method for modelling exposure
ConfounderControl	Short Text	255	Lookup: main method for confounder adjustment
HeterogeneityAssess	Short Text	255	Lookup: main method for assessing heterogeneity
CombineMethod	Short Text	255	Lookup: main method for combining results
CombineMethodText	Long Text	-	Further details of combination methods including quoted text from publication if relevant

CompareExposure	Short Text	255	Lookup: main method for comparing exposure variables in each data source
CompareOutcome	Short Text	255	Lookup: main method for comparing outcome variables in each data source
CompareOther	Short Text	255	Lookup: main method for comparing other variables in each data source
CompareText	Long Text	-	Further details of methods used to compare variables including quoted text from publication if relevant
DataManagement	Short Text	255	Lookup: how was data managed e.g. central vs multicentre etc
DataManageText	Long Text	-	Further details of data management approach including quoted text from publication if relevant
Programming	Short Text	255	Lookup: how was programming managed e.g. central vs multicentre etc
ProgrammingText	Long Text	-	Further details of programming approach including quoted text from publication if relevant

Dedman D, *et al. BMJ Open* 2020; 10:e037405. doi: 10.1136/bmjopen-2020-037405
