# Supplementary material for: Approaches for combining primary care electronic health record data from multiple sources: a systematic review of observational studies
Source: BMJ Open. 2020 Oct 14;10(10):e037405. doi: 10.1136/bmjopen-2020-037405 (PMC7559041; doi:10.1136/bmjopen-2020-037405)
Supplement: Supplementary data [file bmjopen-2020-037405supp002.pdf]

Multidatabase Systematic Review: Supplementary Tables and Figures

Figure S1: Number of included studies by year of publication

Table S1: Summary details of 109 studies included in systematic review

Table S2: Summary details of primary care electronic health record (EHR) data sources used in studies included in systematic review

Figure S1: Number of included studies by year of publication

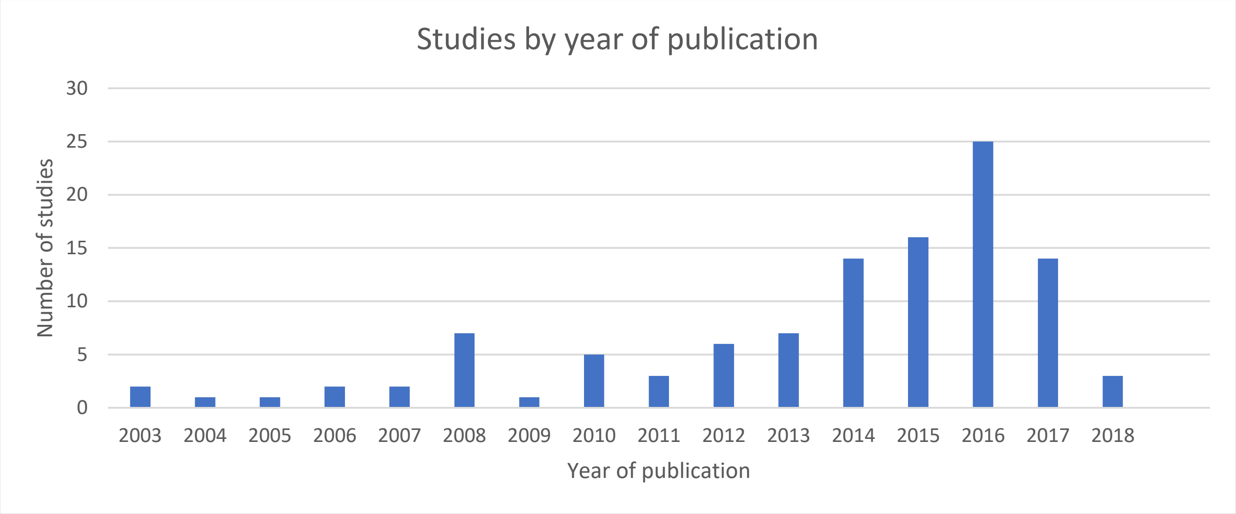

Table S1: Summary details of 109 studies included in systematic review.

Study ID[a]	Citation	Resource locator: [doi unless specified otherwise][b]	Study topic	Study type	Study design[c]	Data combining	Primary care EHR data sources[d]	Other sources (if applicable)
1	Lum KJ, Newcomb CW, Roy JA, et al. Evaluation of methods to estimate missing days' supply within pharmacy data of the Clinical Practice Research Datalink (CPRD) and The Health Improvement Network (THIN). Eur J Clin Pharmacol 2017;73:115–23	10.1007/s00228-016-2148-4	Drug utilization	Other	Other	Data not combined	2: CPRD; THIN	
2	de Bie S, Kaguelidou F, Verhamme KMC, et al. Using Prescription Patterns in Primary Care to Derive New Quality Indicators for Childhood Community Antibiotic Prescribing. Pediatr Infect Dis J 2016;35:1317–23	10.1097/INF.0000000000001324	Drug utilization	Descriptive	Cohort	Data not combined	3: IPCI; Pedianet; THIN	
3	Masclee GMC, Coloma PM, Spaander MCW, et al. NSAIDs, statins, low-dose aspirin and PPIs, and the risk of oesophageal adenocarcinoma among patients with Barrett's oesophagus: a population-based Case-control. BMJ Open 2015;5:e006640	10.1136/bmjopen-2014-006640	Drug safety	Analytical	Case-control	Pooled analysis: individual patient data	2: IPCI; THIN	
7	Laforest L, Licaj I, Devouassoux G, et al. Prescribed therapy for asthma: therapeutic ratios and outcomes. BMC Fam Pract 2015;16:49	10.1186/s12875-015-0265-2	Drug comparative effectiveness	Analytical	Cohort	Data not combined	2: LPD (France); THIN	
8	Afonso A, Schmiedl S, Becker C, et al. A methodological comparison of two European primary care databases and replication in a US claims database: inhaled long-acting beta-2-agonists and the risk of acute myocardial infarction. Eur J Clin Pharmacol 2016;72:1105–16	10.1007/s00228-016-2071-8	Drug safety	Analytical	Cohort	Data not combined	2: CPRD; NPCRD (Mondriaan)	1
9	Ali MS, Groenwold RHH, Belitser S V, et al. Methodological comparison of marginal structural model, time-varying Cox regression, and propensity score methods: the example of antidepressant use and the risk of hip fracture. Pharmacoepidemiol Drug Saf 2016;25 Suppl 1:114–21	10.1002/pds.3864	Drug safety	Analytical	Cohort	Data not combined	3: AHC (Mondriaan); BIFAP; NPCRD (Mondriaan)	
10	Bezemer ID, Verhamme KMC, Gini R, et al. Use of oral contraceptives in three European countries: a population-based multi-database study. Eur J Contracept Reprod Health Care 2016;21:81–7	10.3109/13625187.2015.1102220	Drug utilization	Descriptive	Cohort	Data not combined	3: HSD (Italy); IPCI; THIN	1
11	Brauer R, Douglas I, Garcia Rodriguez LA, et al. Risk of acute liver injury associated with use of antibiotics. Comparative cohort and nested case-control studies using two primary care databases in Europe. Pharmacoepidemiol Drug Saf 2016;25 Suppl 1:29–38	10.1002/pds.3861	Drug safety	Analytical	Multiple: case-control; cohort	Data not combined	2: BIFAP; CPRD	
12	Brauer R, Ruigómez A, Downey G, et al. Prevalence of antibiotic use: a comparison across various European health care data sources. Pharmacoepidemiol Drug Saf 2016;25 Suppl 1:11–20	10.1002/pds.3831	Drug utilization	Descriptive	Cross-sectional	Data not combined	5: AHC (Mondriaan); BIFAP; CPRD; NPCRD (Mondriaan); THIN	2
14	Castellsague J, Perez-Gutthann S, Calingaert B, et al. Characterization of new users of cilostazol in the UK, Spain, Sweden, and Germany. Pharmacoepidemiol Drug Saf 2017;26:615–24	10.1002/pds.4167	Drug utilization	Descriptive	Cohort	Data not combined	2: SIDIAP; THIN	3
15	Charlton RA, Pierini A, Klungsøyr K, et al. Asthma medication prescribing before, during and after pregnancy: a study in seven European regions. BMJ Open 2016;6:e009237	10.1136/bmjopen-2015-009237	Drug utilization	Descriptive	Cohort	Data not combined	2: CPRD; SAIL	5
16	Charlton R, Garne E, Wang H, et al. Antiepileptic drug prescribing before, during and after pregnancy: a study in seven European regions. Pharmacoepidemiol Drug Saf 2015;24:1144–54	10.1002/pds.3847	Drug utilization	Descriptive	Cohort	Data not combined	2: CPRD; SAIL	5
17	Chui CSL, Chan EW, Wong AYS, et al. Association between oral fluoroquinolones and seizures: A self-controlled case series study. Neurology 2016;86:1708–15	10.1212/WNL.0000000000002633	Drug safety	Analytical	SCCS	Meta-analysis: random effects	2: CDARS; CPRD	
18	Clegg A, Bates C, Young J, et al. Development and validation of an electronic frailty index using routine primary care electronic health record data. Age Ageing 2016;45:353–60	10.1093/ageing/afw039	Disease risk prediction	Analytical	Cohort	Data not combined	2: ResearchOne; THIN	
19	De Bortoli N, Ripellino C, Cataldo N, et al. Unspecified intestinal malabsorption in patients treated with angiotensin-converting enzyme inhibitors or angiotensin receptor blockers: a retrospective analysis in primary care settings. Expert Opin Drug Saf 2017;16:1221–5	10.1080/14740338.2017.1376647	Drug safety	Descriptive	Cohort	Pooled analysis: individual patient data	2: Disease Analyzer (Germany); HSD (Italy)	

Study ID[a]	Citation	Resource locator: [doi unless specified otherwise][b]	Study topic	Study type	Study design[c]	Data combining	Primary care EHR data sources[d]	Other sources (if applicable)
20	de Groot MCH, Candore G, Uddin MJ, et al. Case-only designs for studying the association of antidepressants and hip or femur fracture. Pharmacoepidemiol Drug Saf 2016;25 Suppl 1:103–13	10.1002/pds.3850	Drug safety	Analytical	Multiple: CCX; SCCS	Data not combined	3: AHC (Mondriaan); NPCRD (Mondriaan); THIN	
24	Ferrajolo C, Verhamme KMC, Trifirò G, et al. Antibiotic-Induced Liver Injury in Paediatric Outpatients: A Case-control in Primary Care Databases. Drug Saf 2017;40:305–15	10.1007/s40264-016-0493-y	Drug safety	Analytical	Case-control	Pooled analysis: individual patient data	3: HSD (Italy); IPCI; Pedianet	
25	Gold R, Esterberg E, Hollombe C, et al. Low Back Imaging When Not Indicated: A Descriptive Cross-System Analysis. Perm J 2016;20:25–33	10.7812/TPP/15-081	Health services research	Analytical	Cohort	Pooled analysis: individual patient data	2: KP Clarity (Epic EHR); OCHIN Clarity (Epic EHR)	
26	Hippisley-Cox J, Coupland C. Development and validation of risk prediction equations to estimate future risk of blindness and lower limb amputation in patients with diabetes: Cohort. BMJ 2015;351:h5441	10.1136/bmj.h5441	Disease risk prediction	Analytical	Cohort	Data not combined	2: CPRD; QResearch	
27	Hippisley-Cox J, Coupland C. Development and validation of risk prediction equations to estimate future risk of heart failure in patients with diabetes: a prospective Cohort. BMJ Open 2015;5:e008503	10.1136/bmjopen-2015-008503	Disease risk prediction	Analytical	Cohort	Data not combined	2: CPRD; QResearch	
28	Israel E, Roche N, Martin RJ, et al. Increased Dose of Inhaled Corticosteroid versus Add-On Long-acting ß-Agonist for Step-Up Therapy in Asthma. Ann Am Thorac Soc 2015;12:798–806	10.1513/AnnalsATS.201412-580OC	Drug comparative effectiveness	Analytical	Cohort	Pooled analysis: individual patient data	2: CPRD; OPCRD	
30	Levi M, Rosselli M, Simonetti M, et al. Epidemiology of iron deficiency anaemia in four European countries: a population-based study in primary care. Eur J Haematol 2016;97:583–93	10.1111/ejh.12776	Disease epidemiology	Descriptive	Cohort	Data not combined	4: HSD (Italy); LPD (Belgium); LPD (Germany); LPD (Spain)	
31	Levin D, Bell S, Sund R, et al. Pioglitazone and bladder cancer risk: a multipopulation pooled, cumulative exposure analysis. Diabetologia 2015;58:493–504	10.1007/s00125-014-3456-9	Drug safety	Analytical	Cohort	Meta-analysis: fixed & random effects	2: CPRD; SIR	4
32	Murray CS, Thomas M, Richardson K, et al. Comparative Effectiveness of Step-up Therapies in Children with Asthma Prescribed Inhaled Corticosteroids: A Historical Cohort. J allergy Clin Immunol Pract 2017;5:1082-1090.e7	10.1016/j.jaip.2016.12.28	Drug comparative effectiveness	Analytical	Cohort	Pooled analysis: individual patient data	2: CPRD; OPCRD	
33	Nyberg F, Horne L, Morlock R, et al. Comorbidity Burden in Trial-Aligned Patients with Established Gout in Germany, UK, US, and France: a Retrospective Analysis. Adv Ther 2016;33:1180–98	10.1007/s12325-016-0346-1	Disease epidemiology	Descriptive	Cohort	Data not combined	3: CPRD; Disease Analyzer (France); Disease Analyzer (Germany)	1
34	Oteri A, Mazzaglia G, Pecchioli S, et al. Prescribing pattern of antipsychotic drugs during the years 1996-2010: a population-based database study in Europe with a focus on torsadogenic drugs. Br J Clin Pharmacol 2016;82:487–97	10.1111/bcp.12955	Drug utilization	Descriptive	Cohort	Data not combined	3: HSD (Italy); IPCI; THIN	4
35	Petersen I, McCrea RL, Sammon CJ, et al. Risks and benefits of psychotropic medication in pregnancy: cohort studies based on UK electronic primary care health records. Health Technol Assess 2016;20:1–176	10.3310/hta20230	Drug safety	Analytical	Cohort	Pooled analysis: individual patient data	2: CPRD; THIN	
36	Petherick ES, Pickett KE, Cullum NA. Can different primary care databases produce comparable estimates of burden of disease: results of a study exploring venous leg ulceration. Fam Pract 2015;32:374–80	10.1093/fampra/cmv013	Disease epidemiology	Descriptive	Cohort	Data not combined	2: CPRD; THIN	
37	Rathmann W, Czech M, Franek E, et al. Regional differences in insulin therapy regimens in five European countries. Int J Clin Pharmacol Ther 2017;55:403–8	10.5414/CP202906	Drug utilization	Descriptive	Cross-sectional	Data not combined	3: Disease Analyzer (France); Disease Analyzer (Germany); Disease Analyzer (UK)	2
38	Requena G, Huerta C, Gardarsdottir H, et al. Hip/femur fractures associated with the use of benzodiazepines (anxiolytics, hypnotics and related drugs): a methodological approach to assess consistencies across databases from the PROTECT-EU project. Pharmacoepidemiol Drug Saf 2016;25 Suppl 1:66–78	10.1002/pds.3816	Drug safety	Analytical	Multiple: case-control; cohort	Meta-analysis: random effects	3: BIFAP; CPRD; NPCRD (Mondriaan)	
39	Requena G, Logie J, Martin E, et al. Do case-only designs yield consistent results across design and different databases? A case study of hip fractures and benzodiazepines. Pharmacoepidemiol Drug Saf 2016;25 Suppl 1:79–87	10.1002/pds.3822	Drug safety	Analytical	Multiple: CCX;SCCS	Data not combined	2: BIFAP; CPRD	

Study ID[a]	Citation	Resource locator: [doi unless specified otherwise][b]	Study topic	Study type	Study design[c]	Data combining	Primary care EHR data sources[d]	Other sources (if applicable)
40	Saine ME, Carbonari DM, Newcomb CW, et al. Determinants of saxagliptin use among patients with type 2 diabetes mellitus treated with oral anti-diabetic drugs. BMC Pharmacol Toxicol 2015;16:8	10.1186/s40360-015-0007-z	Drug utilization	Analytical	Cross-sectional	Data not combined	2: CPRD; THIN	2
41	Souverein PC, Abbing-Karahagopian V, Martin E, et al. Understanding inconsistency in the results from observational pharmacoepidemiological studies: the case of antidepressant use and risk of hip/femur fractures. Pharmacoepidemiol Drug Saf 2016;25 Suppl 1:88–102	10.1002/pds.3862	Drug safety	Analytical	Multiple: case-control; cohort	Data not combined	4: AHC (Mondriaan); BIFAP; NPCRD (Mondriaan); THIN	
43	Sultana J, Fontana A, Giorgianni F, et al. The Effect of Safety Warnings on Antipsychotic Drug Prescribing in Elderly Persons with Dementia in the United Kingdom and Italy: A Population-Based Study. CNS Drugs 2016;30:1097–109	10.1007/s40263-016-0366-z	Health services research	Analytical	Interrupted time series	Data not combined	2: HSD (Italy); THIN	
44	Turner SW, Richardson K, Burden A, et al. Initial step-up treatment changes in asthmatic children already prescribed inhaled corticosteroids: a historical Cohort. NPJ Prim care Respir Med 2015;25:15041	10.1038/npjpcrm.2015.41	Drug utilization	Descriptive	Cohort	Pooled analysis: individual patient data	2: CPRD; OPCRD	
45	Turner S, Richardson K, Murray C, et al. Long-Acting ß-Agonist in Combination or Separate Inhaler as Step-Up Therapy for Children with Uncontrolled Asthma Receiving Inhaled Corticosteroids. J allergy Clin Immunol Pract 2017;5:99-106.e3	10.1016/j.jaip.2016.06.9	Drug comparative effectiveness	Analytical	Cohort	Pooled analysis: individual patient data	2: CPRD; OPCRD	
46	Tyrstrup M, van der Velden A, Engstrom S, et al. Antibiotic prescribing in relation to diagnoses and consultation rates in Belgium, the Netherlands and Sweden: use of European quality indicators. Scand J Prim Health Care 2017;35:10–8	10.1080/02813432.2017.1288680	Health services research	Descriptive	Cross-sectional	Data not combined	3: Intego; Jonkoping County; Julius (Mondriaan)	
47	Uddin MJ, Groenwold RHH, de Boer A, et al. Evaluating different physician's prescribing preference based instrumental variables in two primary care databases: a study of inhaled long-acting beta2-agonist use and the risk of myocardial infarction. Pharmacoepidemiol Drug Saf 2016;25 Suppl 1:132–41	10.1002/pds.3860	Drug safety	Analytical	Cohort	Pooled analysis: individual patient data	2: CPRD; NPCRD (Mondriaan)	
48	Uddin MJ, Groenwold RHH, de Boer A, et al. Instrumental variables analysis using multiple databases: an example of antidepressant use and risk of hip fracture. Pharmacoepidemiol Drug Saf 2016;25 Suppl 1:122–31	10.1002/pds.3863	Drug safety	Analytical	Cohort	Pooled analysis: individual patient data	3: BIFAP; NPCRD (Mondriaan); THIN	
49	Vinogradova Y, Coupland C, Hippisley-Cox J. Use of combined oral contraceptives and risk of venous thromboembolism: nested case-control studies using the QResearch and CPRD databases. BMJ 2015;350:h2135	10.1136/bmj.h2135	Drug safety	Analytical	Case-control	Meta-analysis: fixed & random effects	2: CPRD; QResearch	
50	Hippisley-Cox J, Coupland C, Brindle P. The performance of seven QPrediction risk scores in an independent external sample of patients from general practice: a validation study. BMJ Open 2014;4:e005809	10.1136/bmjopen-2014-005809	Disease risk prediction	Descriptive	Cohort	Data not combined	2: CPRD; QResearch	
51	Kostev K, Jockwig A, Hallwachs A, et al. Prevalence and risk factors of neuropathy in newly diagnosed type 2 diabetes in primary care practices: a retrospective database analysis in Germany and UK. Prim Care Diabetes 2014;8:250–5	10.1016/j.pcd.2014.01.11	Disease epidemiology	Analytical	Cohort	Data not combined	2: Disease Analyzer (Germany); Disease Analyzer (UK)	
52	Kostev K, Rathmann W. Influence of macro- and microvascular comorbidity on time to insulin initiation in type 2 diabetes patients: a retrospective database analysis in Germany, France, and UK. Prim Care Diabetes 2013;7:167–71	10.1016/j.pcd.2013.02.001	Drug utilization	Analytical	Cohort	Data not combined	3: Disease Analyzer (France); Disease Analyzer (Germany); Disease Analyzer (UK)	
54	Masclee GMC, Coloma PM, de Wilde M, et al. The incidence of Barrett's oesophagus and oesophageal adenocarcinoma in the United Kingdom and The Netherlands is levelling off. Aliment Pharmacol Ther 2014;39:1321–30	10.1111/apt.12759	Disease epidemiology	Descriptive	Cohort	Data not combined	2: IPCI; THIN	
55	Masclee GMC, Valkhoff VE, van Soest EM, et al. Cyclo-oxygenase-2 inhibitors or nonselective NSAIDs plus gastroprotective agents: what to prescribe in daily clinical practice?.Aliment Pharmacol Ther 2013;38:178–89	10.1111/apt.12348	Drug safety	Analytical	Case-control	Multiple: pooled IPD + meta-analysis (RE)	3: CPRD; HSD (Italy); IPCI	
56	de Jonge L, Garne E, Gini R, et al. Improving Information on Maternal Medication Use by Linking Prescription Data to Congenital Anomaly Registers: A EUROmediCAT Study. Drug Saf 2015;38:1083–93	10.1007/s40264-015-0321-9	Disease epidemiology	Descriptive	Cohort	Data not combined	5: AHC (Mondriaan); BIFAP; CPRD; NPCRD (Mondriaan); THIN	2

Study ID[a]	Citation	Resource locator: [doi unless specified otherwise][b]	Study topic	Study type	Study design[c]	Data combining	Primary care EHR data sources[d]	Other sources (if applicable)
57	Ruigómez A, Brauer R, Rodríguez LAG, et al. Ascertainment of acute liver injury in two European primary care databases. Eur J Clin Pharmacol 2014;70:1227–35	10.1007/s00228-014-1721-y	Disease epidemiology	Descriptive	Cohort	Data not combined	2: BIFAP; CPRD	
58	Thomas SL, Minassian C, Ganesan V, et al. Chickenpox and risk of stroke: A self-controlled case series analysis. Clin Infect Dis 2014;58:61–8	10.1093/cid/cit659	Disease epidemiology	Analytical	SCCS	Multiple: pooled IPD + meta-analysis (RE + FE)	4: CPRD; Disease Analyzer (UK); QResearch; THIN	
59	Trifirò G, Mokhles MM, Dieleman JP, et al. Risk of cardiac valve regurgitation with dopamine agonist use in Parkinson's disease and hyperprolactinaemia: a multi-country, nested Case-control. Drug Saf 2012;35:159–71	10.2165/11594940-000000000-00000	Drug safety	Analytical	Case-control	Pooled analysis: individual patient data	3: HSD (Italy); IPCI; THIN	
60	Valkhoff VE, van Soest EM, Masclee GMC, et al. Prescription of nonselective NSAIDs, coxibs and gastroprotective agents in the era of rofecoxib withdrawal - a 617,400-patient study. Aliment Pharmacol Ther 2012;36:790–9	10.1111/apt.12028	Drug utilization	Descriptive	Cohort	Data not combined	3: CPRD; HSD (Italy); IPCI	
61	Valkhoff VE, Coloma PM, Masclee GMC, et al. Validation study in four health-care databases: Upper gastrointestinal bleeding misclassification affects precision but not magnitude of drug-related upper gastrointestinal bleeding risk. J Clin Epidemiol 2014;67:921–31	10.1016/j.jclinepi.2014.02.020	Methodology / data quality	Analytical	Cohort	Pooled analysis: individual patient data	2: HSD (Italy); IPCI	2
62	Valkhoff VE, Schade R, 't Jong GW, et al. Population-based analysis of non-steroidal anti-inflammatory drug use among children in four European countries in the SOS project: what size of data platforms and which study designs do we need to assess safety issues?BMC Pediatr 2013;13:192	10.1186/1471-2431-13-192	Drug utilization	Descriptive	Cohort	Pooled analysis: semi-aggregate data	3: IPCI; Pedianet; THIN	4
63	Valkhoff VE, van Soest EM, Mazzaglia G, et al. Adherence to gastroprotection during cyclooxygenase 2 inhibitor treatment and the risk of upper gastrointestinal tract events: a population-based study. Arthritis Rheum 2012;64:2792–802	10.1002/art.34433	Drug safety	Analytical	Case-control	Multiple: pooled IPD + meta-analysis (RE)	3: CPRD; HSD (Italy); IPCI	
64	Vinogradova Y, Coupland C, Hippisley-Cox J. Exposure to bisphosphonates and risk of common non-gastrointestinal cancers: series of nested case-control studies using two primary-care databases. Br J Cancer 2013;109:795–806	10.1038/bjc.2013.383	Drug safety	Analytical	Case-control	Meta-analysis: fixed effects	2: CPRD; QResearch	
66	Vinogradova Y, Coupland C, Hippisley-Cox J. Exposure to bisphosphonates and risk of gastrointestinal cancers: series of nested case-control studies with QResearch and CPRD data. BMJ 2013;346:f114	10.1136/bmj.f114	Drug safety	Analytical	Case-control	Meta-analysis: fixed effects	2: CPRD; QResearch	
67	Bremner SA, Carey IM, DeWilde S, et al. Infections presenting for clinical care in early life and later risk of hay fever in two UK birth cohorts. Allergy 2008;63:274–83	10.1111/j.1398-9995.2007.01599.x	Disease epidemiology	Analytical	Case-control	Meta-analysis: fixed effects	2: CPRD; DIN	
68	Cooper C, Steinbuch M, Stevenson R, et al. The epidemiology of osteonecrosis: findings from the GPRD and THIN databases in the UK. Osteoporos Int 2010;21:569–77	10.1007/s00198-009-1003-1	Disease epidemiology	Analytical	Case-control	Pooled analysis: individual patient data	2: CPRD; THIN	
69	De Clercq E, Van Casteren V, Jonckheer P, et al. Electronic Patient Record data as proxy of GPs' thoughts. Stud Health Technol Inform 2008;141:103–10	10.3233/978-1-58603-922-6-103	Methodology / data quality	Descriptive	Other	Data not combined	3: Resoprim (x3 networks)	
70	Hippisley-Cox J, Coupland C, Vinogradova Y, et al. Performance of the QRISK cardiovascular risk prediction algorithm in an independent UK sample of patients from general practice: a validation study. Heart 2008;94:34–9	10.1136/hrt.2007.134890	Disease risk prediction	Descriptive	Cohort	Data not combined	2: QResearch; THIN	
71	Hippisley-Cox J, Coupland C. Individualising the risks of statins in men and women in England and Wales: population-based Cohort. Heart 2010;96:939–47	10.1136/hrt.2010.199034	Disease risk prediction	Analytical	Cohort	Data not combined	2: QResearch; THIN	
72	Hippisley-Cox J, Coupland C. Predicting the risk of chronic Kidney Disease in men and women in England and Wales: prospective derivation and external validation of the QKidney Scores. BMC Fam Pract 2010;11:49	10.1186/1471-2296-11-49	Disease risk prediction	Analytical	Cohort	Data not combined	2: QResearch; THIN	
73	Hsia Y, Neubert A, Sturkenboom MCJM, et al. Comparison of antiepileptic drug prescribing in children in three European countries. Epilepsia 2010;51:789–96	10.1111/j.1528-1167.2009.02331.x	Drug utilization	Descriptive	Cohort	Pooled analysis: semi-aggregate data	3: Disease Analyzer (UK); IPCI; Pedianet	
74	Molokhia M, McKeigue P, Curcin V, et al. Statin induced myopathy and myalgia: time trend analysis and comparison of risk associated with statin class from 1991-2006. PLoS One 2008;3:e2522	10.1371/journal.pone.0002522	Drug safety	Analytical	CCX	Data not combined	2: Mediplus; THIN	

Study ID[a]	Citation	Resource locator: [doi unless specified otherwise][b]	Study topic	Study type	Study design[c]	Data combining	Primary care EHR data sources[d]	Other sources (if applicable)
75	Neubert A, Verhamme K, Murray ML, et al. The prescribing of analgesics and non-steroidal anti-inflammatory drugs in paediatric primary care in the UK, Italy and the Netherlands. Pharmacol Res 2010;62:243–8	10.1016/j.phrs.2010.04.006	Drug utilization	Descriptive	Cohort	Data not combined	3: Disease Analyzer (UK); IPCI; Pedianet	
76	Pfeil N, Uhlig U, Kostev K, et al. Antiemetic medications in children with presumed infectious gastroenteritis--pharmacoepidemiology in Europe and Northern America. J Pediatr 2008;153:659–62, 662.e1-3	10.1016/j.jpeds.2008.07.50	Drug utilization	Descriptive	Cohort	Data not combined	3: Disease Analyzer (France); Disease Analyzer (Germany); Disease Analyzer (UK)	2
77	Sturkenboom MCJM, Dieleman JP, Picelli G, et al. Prevalence and treatment of hypertensive patients with multiple concomitant cardiovascular risk factors in The Netherlands and Italy. J Hum Hypertens 2008;22:704–13	10.1038/jhh.2008.82	Disease epidemiology	Descriptive	Cohort	Data not combined	2: HSD (Italy); IPCI	
78	Sturkenboom MCJM, Verhamme KMC, Nicolosi A, et al. Drug use in children: Cohort in three European countries. BMJ 2008;337:a2245–a2245	10.1136/bmj.a2245	Drug utilization	Descriptive	Cohort	Pooled analysis: semi-aggregate data	3: Disease Analyzer (UK); IPCI; Pedianet	
79	van Soest EM, Valkhoff VE, Mazzaglia G, et al. Suboptimal gastroprotective coverage of NSAID use and the risk of upper gastrointestinal bleeding and ulcers: an observational study using three European databases. Gut 2011;60:1650–9	10.1136/gut.2011.239848	Drug safety	Analytical	Case-control	Pooled analysis: individual patient data	3: CPRD; HSD (Italy); IPCI	
80	van Staa TP, Sprafka JM. Study of adverse outcomes in women using testosterone therapy. Maturitas 2009;62:76–80	10.1016/j.maturitas.2008.11.001	Drug safety	Analytical	Cohort	Pooled analysis: individual patient data	2: CPRD; THIN	
81	Brankin E, Walker M, Lynch N, et al. The impact of dosing frequency on compliance and persistence with bisphosphonates among postmenopausal women in the UK: evidence from three databases. Curr Med Res Opin 2006;22:1249–56	10.1185/030079906X112688	Drug utilization	Analytical	Cohort	Data not combined	3: CPRD; DIN; Disease Analyzer (UK)	
82	Bremner SA, Carey IM, DeWilde S, et al. Early-life exposure to antibacterials and the subsequent development of hayfever in childhood in the UK: case-control studies using the General Practice Research Database and the Doctors' Independent Network. Clin Exp Allergy 2003;33:1518–25	10.1046/j.1365-2222.2003.01794.x	Disease epidemiology	Analytical	Case-control	Meta-analysis: fixed effects	2: CPRD; DIN	
83	Bremner SA, Carey IM, DeWilde S, et al. Timing of routine immunisations and subsequent hay fever risk. Arch Dis Child 2005;90:567–73	10.1136/adc.2004.051714	Disease epidemiology	Analytical	Case-control	Meta-analysis: fixed effects	2: CPRD; DIN	
84	Bremner SA, Carey IM, DeWilde S, et al. Vaccinations, infections and antibacterials in the first grass pollen season of life and risk of later hayfever. Clin Exp Allergy 2007;37:512–7	10.1111/j.1365-2222.2007.02697.x	Disease epidemiology	Analytical	Case-control	Meta-analysis: fixed effects	2: CPRD; DIN	
86	Carey IM, Cook DG, De Wilde S, et al. Implications of the problem orientated medical record (POMR) for research using electronic GP databases: a comparison of the Doctors Independent Network Database (DIN) and the General Practice Research Database (GPRD). BMC Fam Pract 2003;4:14	10.1186/1471-2296-4-14	Methodology / data quality	Descriptive	Cohort	Data not combined	2: CPRD; DIN	
88	De Wilde S, Carey IM, Bremner SA, et al. A comparison of the recording of 30 common childhood conditions in the Doctor's Independent Network and General Practice Research Databases. Heal Stat Q 2004;:21–31	pmid:15704391	Methodology / data quality	Descriptive	Cohort	Data not combined	2: CPRD; DIN	
89	Hernández-Díaz S, García Rodríguez LA. Cardioprotective aspirin users and their excess risk of upper gastrointestinal complications. BMC Med 2006;4:22	10.1186/1741-7015-4-22	Drug safety	Descriptive	Cohort	Data not combined	2: BIFAP; CPRD	
90	Jordan K, Clarke AM, Symmons DPM, et al. Measuring disease prevalence: a comparison of musculoskeletal disease using four general practice consultation databases. Br J Gen Pract 2007;57:7–14	pmid:17244418	Disease epidemiology	Descriptive	Cohort	Data not combined	2: CPRD; CiPCA	2
93	Price DB, Gefen E, Gopalan G, et al. Real-life effectiveness and safety of salbutamol Steri-NebTM vs. Ventolin Nebules® for exacerbations in patients with COPD: Historical Cohort. PLoS One 2018;13:e0191404	10.1371/journal.pone.0191404	Drug comparative effectiveness	Analytical	Cohort	Pooled analysis: individual patient data	2: CPRD; OPCRD	
94	Abbing-Karahagopian V, Huerta C, Souverein PC, et al. Antidepressant prescribing in five European countries: application of common definitions to assess the prevalence, clinical observations, and methodological implications. Eur J Clin Pharmacol 2014;70:849–57	10.1007/s00228-014-1676-z	Drug utilization	Descriptive	Cohort	Data not combined	5: AHC (Mondriaan); BIFAP; CPRD; NPCRD (Mondriaan); THIN	2

Study ID[a]	Citation	Resource locator: [doi unless specified otherwise][b]	Study topic	Study type	Study design[c]	Data combining	Primary care EHR data sources[d]	Other sources (if applicable)
101	Charlton RA, Jordan S, Pierini A, et al. Selective serotonin reuptake inhibitor prescribing before, during and after pregnancy: a population-based study in six European regions. BJOG 2015;122:1010–20	10.1111/1471-0528.13143	Drug utilization	Descriptive	Cohort	Data not combined	2: CPRD; SAIL	4
102	Coloma PM, de Ridder M, Bezemer I, et al. Risk of cardiac valvulopathy with use of bisphosphonates: a population-based, multi-country Case-control. Osteoporos Int 2016;27:1857–67	10.1007/s00198-015-3441-2	Drug safety	Analytical	Case-control	Multiple: pooled IPD + meta-analysis (RE + FE)	3: HSD (Italy); IPCI; THIN	3
103	Coloma PM, Schuemie MJ, Trifirò G, et al. Drug-induced acute myocardial infarction: identifying 'prime suspects' from electronic healthcare records-based surveillance system. PLoS One 2013;8:e72148	10.1371/journal.pone.0072148	Methodology / data quality	Analytical	Cohort	Pooled analysis: individual patient data	3: HSD (Italy); IPCI; Pedianet	4
104	Coloma PM, Trifirò G, Schuemie MJ, et al. Electronic healthcare databases for active drug safety surveillance: is there enough leverage?.Pharmacoepidemiol Drug Saf 2012;21:611–21	10.1002/pds.3197	Drug safety	Descriptive	Cohort	Pooled analysis: semi-aggregate data	4: HSD (Italy); IPCI; Pedianet; QResearch	4
105	de Bie S, Coloma PM, Ferrajolo C, et al. The role of electronic healthcare record databases in paediatric drug safety surveillance: a retrospective Cohort. Br J Clin Pharmacol 2015;80:304–14	10.1111/bcp.12610	Drug safety	Descriptive	Cohort	Pooled analysis: semi-aggregate data	3: HSD (Italy); IPCI; Pedianet	4
106	de Groot MCH, Klungel OH, Leufkens HGM, et al. Sources of heterogeneity in case-control studies on associations between statins, ACE-inhibitors, and proton pump inhibitors and risk of pneumonia. Eur J Epidemiol 2014;29:767–75	10.1007/s10654-014-9941-0	Drug safety	Analytical	Case-control	Data not combined	3: AHC (Mondriaan); LRGP; NPCRD (Mondriaan)	2
107	de Groot MCH, Schuerch M, de Vries F, et al. Antiepileptic drug use in seven electronic health record databases in Europe: a methodologic comparison. Epilepsia 2014;55:666–73	10.1111/epi.12557	Drug utilization	Descriptive	Cohort	Data not combined	5: AHC (Mondriaan); BIFAP; CPRD; NPCRD (Mondriaan); THIN	2
108	Ferrajolo C, Coloma PM, Verhamme KMC, et al. Signal detection of potentially drug-induced acute liver injury in children using a multi-country healthcare database network. Drug Saf 2014;37:99–108	10.1007/s40264-013-0132-9	Drug safety	Analytical	Multiple: cohort; SCCS	Pooled analysis: individual patient data	3: HSD (Italy); IPCI; Pedianet	4
110	Huerta C, Abbing-Karahagopian V, Requena G, et al. Exposure to benzodiazepines (anxiolytics, hypnotics and related drugs) in seven European electronic healthcare databases: a cross-national descriptive study from the PROTECT-EU Project. Pharmacoepidemiol Drug Saf 2016;25 Suppl 1:56–65	10.1002/pds.3825	Drug utilization	Descriptive	Cohort	Data not combined	5: AHC (Mondriaan); BIFAP; CPRD; NPCRD (Mondriaan); THIN	2
111	Korhonen P, Heintjes EM, Williams R, et al. Pioglitazone use and risk of bladder cancer in patients with type 2 diabetes: retrospective Cohort using datasets from four European countries. BMJ 2016;354:i3903	10.1136/bmj.i3903	Drug safety	Analytical	Cohort	Multiple: pooled IPD + meta-analysis (RE + FE)	2: CPRD; PHARMO GP	3
112	LoCasale R, Kern DM, Chevalier P, et al. Description of cardiovascular event rates in patients initiating chronic opioid therapy for noncancer pain in observational cohort studies in the US, UK, and Germany. Adv Ther 2014;31:708–23	10.1007/s12325-014-0131-y	Drug safety	Descriptive	Cohort	Data not combined	2: CPRD; Disease Analyzer (Germany)	1
113	Masclee GMC, Valkhoff VE, Coloma PM, et al. Risk of upper gastrointestinal bleeding from different drug combinations. Gastroenterology 2014;147:784-792.e9	10.1053/j.gastro.2014.06.7	Drug safety	Analytical	SCCS	Multiple: pooled IPD + meta-analysis (RE)	3: HSD (Italy); IPCI; Pedianet	4
114	Mokhles MM, Trifirò G, Dieleman JP, et al. The risk of new onset heart failure associated with dopamine agonist use in Parkinson's disease. Pharmacol Res 2012;65:358–64	10.1016/j.phrs.2011.11.009	Drug safety	Analytical	Case-control	Multiple: pooled IPD + meta-analysis (RE + FE)	3: HSD (Italy); IPCI; THIN	1
115	Rottenkolber M, Voogd E, van Dijk L, et al. Seasonal changes in prescribing of long-acting beta-2-agonists-containing drugs. Respir Med 2015;109:828–37	10.1016/j.rmed.2015.01.010	Drug utilization	Descriptive	Cohort	Data not combined	5: AHC (Mondriaan); BIFAP; CPRD; NPCRD	1

Study ID[a]	Citation	Resource locator: [doi unless specified otherwise][b]	Study topic	Study type	Study design[c]	Data combining	Primary care EHR data sources[d]	Other sources (if applicable)
							(Mondriaan); THIN	
116	Rottenkolber M, Voogd E, van Dijk L, et al. Time trends of period prevalence rates of patients with inhaled long-acting beta-2-agonists-containing prescriptions: a European comparative database study. PLoS One 2015;10:e0117628	10.1371/journal.pone.0117628	Drug utilization	Descriptive	Cohort	Data not combined	5: AHC (Mondriaan); BIFAP; CPRD; NPCRD (Mondriaan); THIN	2
117	Trifirò G, de Ridder M, Sultana J, et al. Use of azithromycin and risk of ventricular arrhythmia. CMAJ 2017;189:E560–8	10.1503/cmaj.160355	Drug safety	Analytical	Case-control	Multiple: pooled IPD + meta-analysis (RE)	3: HSD (Italy); IPCI; THIN	4
118	Tyczynski JE, Oleske DM, Klingman D, et al. Safety assessment of an anti-obesity drug (sibutramine): a retrospective Cohort. Drug Saf 2012;35:629–44	10.2165/11599220-000000000-00000	Drug safety	Analytical	Cohort	Data not combined	2: Disease Analyzer (Germany); Disease Analyzer (UK)	
119	Coloma PM, Schuemie MJ, Trifirò G, et al. Combining electronic healthcare databases in Europe to allow for large-scale drug safety monitoring: the EU-ADR Project. Pharmacoepidemiol Drug Saf 2011;20:1–11	10.1002/pds.2053	Drug safety	Analytical	Cohort	Data not combined	4: HSD (Italy); IPCI; Pedianet; QResearch	4
121	Sen EF, Verhamme KMC, Neubert A, et al. Assessment of pediatric asthma drug use in three European countries; a TEDDY study. Eur J Pediatr 2011;170:81–92	10.1007/s00431-010-1275-7	Drug utilization	Descriptive	Cohort	Data not combined	3: Disease Analyzer (UK); IPCI; Pedianet	
122	Cainzos-Achirica M, Varas-Lorenzo C, Pottegård A, et al. Methodological challenges when evaluating potential off-label prescribing of drugs using electronic health care databases: A case study of dabigatran etexilate in Europe. Pharmacoepidemiol Drug Saf 2018;27:713–23	10.1002/pds.4416	Drug utilization	Descriptive	Cross-sectional	Data not combined	2: CPRD; LPD (France)	1
124	Martín-Merino E, Petersen I, Hawley S, et al. Risk of venous thromboembolism among users of different anti-osteoporosis drugs: a population-based cohort analysis including over 200,000 participants from Spain and the UK. Osteoporos Int 2018;29:467–78	10.1007/s00198-017-4308-5	Drug safety	Analytical	Cohort	Data not combined	2: BIFAP; CPRD	
125	Anyanwagu U, Owen K, Mamza J, et al. Demographics, insulin use and clinical targets in type 2 diabetes insulin users: comparison of a local integrated diabetes service vs a UK-wide cohort. Pract Diabetes 2017;34:123–8	10.1002/pdi.2099	Health services research	Descriptive	Cross-sectional	Data not combined	2: EIDS; THIN	
127	Chiquette E, Oral EA, Garg A, et al. Estimating the prevalence of generalized and partial lipodystrophy: findings and challenges. Diabetes Metab Syndr Obes 2017;10:375–83	10.2147/DMSO.S130810	Disease epidemiology	Descriptive	Cross-sectional	Data not combined	2: CPRD; Humedica	2
128	Lo Re V, Carbonari DM, Saine ME, et al. Postauthorization safety study of the DPP-4 inhibitor saxagliptin: a large-scale multinational family of cohort studies of five outcomes. BMJ open diabetes Res care 2017;5:e000400	10.1136/bmjdrc-2017-000400	Drug safety	Analytical	Cohort	Meta-analysis: method not specified	2: CPRD; THIN	2
129	Price D, Thomas V, von Ziegenweidt J, et al. Switching patients from other inhaled corticosteroid devices to the Easyhaler(®): historical, matched-Cohort of real-life asthma patients. J Asthma Allergy 2014;7:31–51	10.2147/JAA.S59386	Drug comparative effectiveness	Analytical	Cohort	Pooled analysis: individual patient data	2: CPRD; OPCRD	
134	Coloma PM, Valkhoff VE, Mazzaglia G, et al. Identification of acute myocardial infarction from electronic healthcare records using different disease coding systems: a validation study in three European countries. BMJ Open 2013;3:e002862	10.1136/bmjopen-2013-002862	Drug safety	Analytical	Cohort	Pooled analysis: individual patient data	2: HSD (Italy); IPCI	1
135	Mor A, Frøslev T, Thomsen RW, et al. Antibiotic use varies substantially among adults: a cross-national study from five European Countries in the ARITMO project. Infection 2015;43:453–72	10.1007/s15010-015-0768-8	Drug utilization	Descriptive	Cohort	Data not combined	2: HSD (Italy); THIN	4
136	Poluzzi E, Diemberger I, De Ridder M, et al. Use of antihistamines and risk of ventricular tachyarrhythmia: a nested Case-control in five European countries from the ARITMO project. Eur J Clin Pharmacol 2017;73:1499–510	10.1007/s00228-017-2317-0	Drug safety	Analytical	Case-control	Multiple: pooled IPD + meta-analysis (RE)	3: HSD (Italy); IPCI; THIN	4
137	Roberto G, Leal I, Sattar N, et al. Identifying Cases of Type 2 Diabetes in Heterogeneous Data Sources: Strategy from the EMIF Project. PLoS One 2016;11:e0160648	10.1371/journal.pone.0160648	Methodology / data quality	Other	Cross-sectional	Data not combined	3: HSD (Italy); IPCI; THIN	5

Study ID[a]	Citation	Resource locator: [doi unless specified otherwise][b]	Study topic	Study type	Study design[c]	Data combining	Primary care EHR data sources[d]	Other sources (if applicable)
138	La Gamba F, Corrao G, Romio S, et al. Combining evidence from multiple electronic health care databases: performances of one-stage and two-stage meta-analysis in matched case-control studies. Pharmacoepidemiol Drug Saf 2017;26:1213–9	10.1002/pds.4280	Drug safety	Analytical	Case-control	Multiple: pooled IPD + meta-analysis (FE)	2: IPCI; THIN	1

[a] Record identifier used in reference management, and different from reference number used in main study text.
[b] If digital object identifier [doi] was not available, Pubmed ID [pmid] was used
[c] SCCS – self controlled case series; CCX – case crossover:
[d] See Table S2 for further details of primary care EHR data sources

Table S2: Summary details of primary care electronic health record (EHR) data sources used in studies included in systematic review.

Data Source ID	Short name	Source Name	Country	Clinical Coding	Drug Coding	Source reference	Resource locator[a]
1	CPRD	Clinical Practice Research Datalink	UK	Read V2	DM+D	https://www.ncbi.nlm.nih.gov/pubmed/26050254	doi: 10.1093/ije/dyv098
2	THIN	The Health Information Network	UK	Read V2	DM+D	https://www.ncbi.nlm.nih.gov/pubmed/22828580	doi: 10.14236/jhi.v19i4.820
3	QResearch	QResearch	UK	Read V2 / SNOMED-CT	DM+D	https://bmjopen.bmj.com/content/5/9/e008503.long	doi:10.1136/bmjopen-2015-008503
4	IPCI	Integrated Primary Care Information database	Netherlands	ICPC	ATC	https://www.ncbi.nlm.nih.gov/pubmed/10805025	doi:10.1055/s-0038-1634402
5	Pedianet	PediaNet	Italy		ATC	https://www.ncbi.nlm.nih.gov/pubmed/15930187	doi:10.1542/peds.2004-0040
7	SAIL	SAIL Databank	UK	Mixed	DM+D	http://www.ncbi.nlm.nih.gov/pubmed/19732426	doi:10.1186/1472-6963-9-157
8	LPD (France)	Cegedim LPD (Longitudinal Patient Data): France	France	ICD-9/10 CM	ATC	https://epidemiologie-france.aviesan.fr/en/ccontent/pdf/(ObjectId)/91221	url:https://epidemiologie-france.aviesan.fr/en/ccontent/pdf/(ObjectId)/91221
9	NPCRD (Mondriaan)	Netherlands Primary Care Research Database (NPCRD)	Netherlands	ICPC/ICD	ATC	http://www.ncbi.nlm.nih.gov/pubmed/25154551	doi:10.1016/s1098-3015(10)74933-0
11	BIFAP	Spanish Base de datos para la Investigación Farmacoepidemiológica en Atención Primaria (BIFAP)	Spain	ICPC	ATC	https://www.ncbi.nlm.nih.gov/pubmed/25066450	doi:10.1007/s00228-012-1386-3
12	HSD (Italy)	Health Search Database (Italy)	Italy	ICD-9 CM	ATC	https://www.ncbi.nlm.nih.gov/pubmed/24816637	doi:10.1371/journal.pone.0095419
15	AHC (Mondriaan)	Mondriaan Almere Healthcare group (AHC) database	Netherlands	ICPC/ICD	ATC	https://www.valueinhealthjournal.com/article/S1098-3015(10)74933-0/abstract	doi:10.1016/s1098-3015(10)74933-0
17	SIDIAP	Sistema de Información para el desarrollo de la Investigación en Atención Primaria	Spain	ICD-10	ATC	http://www.sidiap.org	url:http://www.sidiap.org
25	CDARS	Hong Kong Clinical Data Analysis and Reporting System database	Hong Kong	ICD-9 CM		https://www.ncbi.nlm.nih.gov/pubmed/24833754	doi:10.1093/jac/dku145
26	ResearchOne	ResearchOne	UK	CTV3	DM+D	https://www.ncbi.nlm.nih.gov/pubmed/26944937	url:http://www.researchone.org/
28	Julius (Mondriaan)	Julius Primary Care Network Database	Netherlands		ATC	https://www.ncbi.nlm.nih.gov/pubmed/30253760	doi:10.1016/s1098-3015(10)74933-0
29	NWEH-LDB	NorthWest EHealth linked database	UK	Read V2/SNOMED-CT	DM+D	https://bmcmedinformdecismak.biomedcentral.com/articles/10.1186/s12911-015-0132-z	doi:10.1186/s12911-015-0132-z
30	OCHIN Clarity (Epic EHR)	Oregon Community Health Information Network (OCHIN)	USA	ICD-9 CM	ATC	https://www.ncbi.nlm.nih.gov/pubmed/26934626	doi:10.7812/TPP/15-081
31	KP Clarity (Epic EHR)	Kaiser Permanente Epic EHR Clarity database	USA	ICD-9 CM	ATC	https://www.ncbi.nlm.nih.gov/pubmed/26934626	doi:10.7812/TPP/15-081
32	OPCRD	Optimum Patient Care Research Database (OPCRD)	UK	Read V2	DM+D	https://optimumpatientcare.org/database-overview/	url:https://optimumpatientcare.org/database-overview/
33	LPD (Spain)	IMS Longitudinal Patient Database (Spain)	Spain	ICD-9/10 CM	ATC	https://www.ncbi.nlm.nih.gov/pubmed/27155295	doi:10.1111/ejh.12776
34	LPD (Germany)	IMS Longitudinal Patient Database (Germany)	Germany	ICD-9/10 CM	ATC	https://www.ncbi.nlm.nih.gov/pubmed/27155295	doi:10.1111/ejh.12776
35	LPD (Belgium)	IMS Longitudinal Patient Database (Belgium)	Belgium	ICD-9/10 CM	ATC	https://www.ncbi.nlm.nih.gov/pubmed/27155295	doi:10.1111/ejh.12776
39	SIR	Salford Integrated Record (SIR)	UK	Read V2	DM+D	https://www.ncbi.nlm.nih.gov/pubmed/25481707	doi:10.1007/s00125-014-3456-9
41	Disease Analyzer (Germany)	IMS Disease Analyzer (Germany)	Germany	ICD-10	ATC	https://www.ncbi.nlm.nih.gov/pubmed/19825325	doi:10.5414/cpp47617
42	Disease Analyzer (France)	IMS Disease Analyzer (France)	France	ICD-10	ATC	https://www.ncbi.nlm.nih.gov/pubmed/19825325	doi:10.5414/cpp47617

Data Source ID	Short name	Source Name	Country	Clinical Coding	Drug Coding	Source reference	Resource locator[a]
46	Disease Analyzer (UK)	IMS Disease Analyzer (UK)	UK	ICD-10	ATC	https://bpspubs.onlinelibrary.wiley.com/doi/full/10.1111/j.1365-2125.2005.02450.x	doi:10.1111/j.1365-2125.2005.02450.x
51	DIN	Doctors' Independent Network (DIN)	UK	Read V2	BNF	https://www.ncbi.nlm.nih.gov/pubmed/15171985	doi:10.1016/j.ijmedinf.2004.02.002
52	Intego	Intego Network	Belgium	ICPC	ATC	http://www.ncbi.nlm.nih.gov/pubmed/24906941	doi:10.1186/1472-6947-14-48
53	Jonkoping County	Jonkoping County Primary Care Database	Sweden	ICD-10	ATC	https://www.ncbi.nlm.nih.gov/pubmed/28277045	doi:10.1080/02813432.2017.1288680
58	Resoprim	Resoprim Project	Belgium	ICPC	ATC	https://www.ncbi.nlm.nih.gov/pubmed/18953130	doi:10.3233/978-1-58603-922-6-103
59	Mediplus	IMS Mediplus	UK	Read V2	BNF	https://www.ncbi.nlm.nih.gov/pubmed/16368704	doi:10.1093/fampra/cmi106
62	CiPCA	Consultations in Primary Care Archive	UK	Read V2	BNF	https://www.keele.ac.uk/mrr/cipcadatabase/	url:https://www.keele.ac.uk/mrr/cipcadatabase/
65	THALES	THALES Database	France	ICD-9 CM	ATC	http://www.ncbi.nlm.nih.gov/pubmed/11111209	doi:10.1159/000052402
72	LRGP	Leidsche Rijn GP database (Mondriaan)	Netherlands	ICPC	ATC	https://www.ncbi.nlm.nih.gov/pubmed/15921047	doi:10.1007/s10654-004-5689-2
74	PHARMO GP	PHARMO GP database	Netherlands	ICPC	ATC	https://www.ncbi.nlm.nih.gov/pubmed/27530399	doi:10.1136/bmj.i3903
76	EIDS	Erewash (Integrated) Diabetes Service	UK	Read V2	DM+D	https://onlinelibrary.wiley.com/doi/full/10.1002/pdi.2099	doi:10.1002/pdi.2099
79	Humedica	Humedica NorthStar from Optum	USA	ICD-9 CM	ATC	https://www.nihcollaboratory.org/Pages/OptumInsight-MetaData-Table.aspx	doi:10.2147/DMSO.S130810
80	GE Healthcare	GE Healthcare Database	USA	ICD-9 CM	ATC	https://www.ncbi.nlm.nih.gov/pubmed/29066925	doi:10.2147/DMSO.S130810

[a] doi: Digital object identifier; url: Uniform resource locator
