# Supplementary material for: Approaches for combining primary care electronic health record data from multiple sources: a systematic review of observational studies
Source: BMJ Open. 2020 Oct 14;10(10):e037405. doi: 10.1136/bmjopen-2020-037405 (PMC7559041; doi:10.1136/bmjopen-2020-037405)
Supplement: Author's manuscript [file bmjopen-2020-037405.draft_revisions.pdf]

BMJ Open is committed to open peer review. As part of this commitment we make the peer review history of every article we publish publicly available.

When an article is published we post the peer reviewers' comments and the authors' responses online. We also post the versions of the paper that were used during peer review. These are the versions that the peer review comments apply to.

The versions of the paper that follow are the versions that were submitted during the peer review process. They are not the versions of record or the final published versions. They should not be cited or distributed as the published version of this manuscript.

BMJ Open is an open access journal and the full, final, typeset and author-corrected version of record of the manuscript is available on our site with no access controls, subscription charges or pay-per-view fees (http://bmjopen.bmj.com).

If you have any questions on BMJ Open's open peer review process please email info.bmjopen@bmj.com

BMJ Open

Approaches for combining primary care electronic health record data from multiple sources: a systematic review of observational studies

Journal:	*BMJ Open*
Manuscript ID	bmjopen-2020-037405
Article Type:	Original research
Date Submitted by the Author:	31-Jan-2020
Complete List of Authors:	Dedman, Daniel; Medicines and Healthcare Products Regulatory Agency, Clinical Practice Research Datalink; London School of Hygiene and Tropical Medicine, Faculty of Epidemiology and Population Health<br>Cabecinha, Melissa; University College London, Research Department of Primary Care and Population Health<br>Williams, Rachael; Medicines and Healthcare Products Regulatory Agency, Clinical Practice Research Datalink<br>Evans, Stephen; London School of Hygiene and Tropical Medicine, Faculty of Epidemiology and Population Health<br>Bhaskaran, Krishnan; London School of Hygiene and Tropical Medicine, Faculty of Epidemiology and Population Health<br>Douglas, Ian; London School of Hygiene and Tropical Medicine, Faculty of Epidemiology and Population Health
Keywords:	EPIDEMIOLOGY, STATISTICS & RESEARCH METHODS, PUBLIC HEALTH

SCHOLARONE™
Manuscripts

I, the Submitting Author has the right to grant and does grant on behalf of all authors of the Work (as defined in the below author licence), an exclusive licence and/or a non-exclusive licence for contributions from authors who are: i) UK Crown employees; ii) where BMJ has agreed a CC-BY licence shall apply, and/or iii) in accordance with the terms applicable for US Federal Government officers or employees acting as part of their official duties; on a worldwide, perpetual, irrevocable, royalty-free basis to BMJ Publishing Group Ltd ("BMJ") its licensees and where the relevant Journal is co-owned by BMJ to the co-owners of the Journal, to publish the Work in this journal and any other BMJ products and to exploit all rights, as set out in our licence.

The Submitting Author accepts and understands that any supply made under these terms is made by BMJ to the Submitting Author unless you are acting as an employee on behalf of your employer or a postgraduate student of an affiliated institution which is paying any applicable article publishing charge ("APC") for Open Access articles. Where the Submitting Author wishes to make the Work available on an Open Access basis (and intends to pay the relevant APC), the terms of reuse of such Open Access shall be governed by a Creative Commons licence – details of these licences and which Creative Commons licence will apply to this Work are set out in our licence referred to above.

Other than as permitted in any relevant BMJ Author's Self Archiving Policies, I confirm this Work has not been accepted for publication elsewhere, is not being considered for publication elsewhere and does not duplicate material already published. I confirm all authors consent to publication of this Work and authorise the granting of this licence.

For peer review only

Title: Approaches for combining primary care electronic health record data from multiple sources: a systematic review of observational studies

Authors:

Daniel Dedman[1,2], Melissa Cabecinha[3], Rachael Williams[1], Stephen J W Evans[2], Krishnan Bhaskaran[2], Ian J Douglas[2]

Affiliations:

1. Clinical Practice Research Datalink, Medicines and Healthcare products Regulatory Agency, London, UK.

2. Faculty of Epidemiology and Population Health, London School of Hygiene and Tropical Medicine, London, UK

3. Research Department of Primary Care and Population Health, University College London, London, UK

Corresponding author: Daniel Dedman.

Address: Clinical Practice Research Datalink (CPRD), 10 South Colonnade, Canary Wharf, London E14 4PU, UK.

Telephone: +44 (0)203 080 6703

Email: daniel.dedman@mhra.gov.uk

ORCID iD: 0000-0002-3699-5391

For peer review only

Abstract

Objective

To identify observational studies which used data from more than one primary care electronic health record (EHR) database, and summarise key characteristics including: objective and rationale for using multiple data sources; methods used to manage, analyse, and (where applicable) combine data; and describe the approaches used to assess and report heterogeneity between data sources.

Design

Systematic review of published studies.

Data sources

Pubmed and Embase databases, searched using list of named primary care EHR databases; supplementary hand searches of reference list of studies retained after initial screening.

Study selection

Observational studies published between 2000-2018 which included at least 2 different primary care EHR databases.

Results

6054 studies were identified from database and hand searches, and 109 were included in the final review, the majority published between 2014-2018. Included studies used 38 different primary care EHR data sources.

Forty-seven studies (44%) were descriptive or methodological. Of 62 analytical studies, 22 (36%) presented separate results from each database, with no attempt to combine them; 29 (48%) combined individual patient data in a 1-stage meta-analysis; and 21 (34%) combined estimates from each database using 2-stage meta-analysis. Discussion and exploration of heterogeneity was inconsistent across studies.

Conclusions

Comparing patterns and trends in different populations, or in different primary care EHR databases from the same populations, are important and common objectives for multi-database studies.

When combining results from several databases using meta-analysis, provision of separate results from each database is helpful for interpretation. We found that these were often missing, particularly for studies using 1-stage approaches, which also often lacked details of any statistical adjustment for heterogeneity and/or clustering. For 2 stage meta-analysis a clear rationale should be provided for choice of fixed effect (FE), and/or random effects (RE) or other models.

For peer review only

Article summary

Strengths and limitations of this study

- Our systematic review identified and described the full and increasing range of completed studies which brought together primary care electronic health record data from two or more sources.
- Exploration of between database heterogeneity was reported inconsistently and reporting of methods for 1-stage meta-analysis was often inadequate.
- The findings of this review may not apply to multi-database studies which did not use primary care EHR data sources.

For peer review only

Keywords

Systematic review

Multi-database studies

Electronic health records

Primary care

Word count

3475 words (not including title page, abstract, references, figures, tables or acknowledgments)

Introduction

Multi-database observational studies are increasingly common. They are conducted for two main reasons: to compare results across diverse populations and healthcare settings; or to combine the data to increase statistical power. Primary care electronic health record (EHR) databases are particularly valuable because they provide longitudinal data on individuals, often over many years, and typically contain richer information on a broader range of exposures, risk factors and health outcomes than administrative databases.[1–3] Although individual primary care EHR databases are often relatively small, covering a single region or other national population subset, their growing availability in recent years is likely to further increase the importance of non-interventional studies which combine these databases. Guidelines identifying best practice in this context have yet to be established but would be of clear benefit for researchers working with multiple databases. An important preliminary step is to describe current practice, but there is no comprehensive summary of studies which used two or more primary care EHR databases, and the methods for combining them.

One previous systematic review focussed on multi-database pharmacoepidemiology studies with a pre-planned approach to combine data to evaluate drug-outcome associations.[4] In that review studies were not limited according to the types of databases used, but descriptive studies and those which did not combine results from different databases were excluded. The authors found that for data management arrangements, analysis of heterogeneity, and methods for combining data reporting was often inadequate, making interpretation of study results more challenging. Since the focus of that review was pharmacoepidemiology studies and a wide range of database types, a broader view of combined primary care EHR data for any study purpose remains lacking.

The aim of this systematic review was to identify and describe the full range of completed studies which brought together primary care electronic health record (EHR) data from two or more sources. The specific objectives were: to summarise key study characteristics, including the main reasons or motivations for including data from different EHR databases; to describe the methods used to manage, analyse, and (where applicable) combine data; and describe the approaches used to assess and report heterogeneity between primary care EHR data sources.

Methods

The review considered all multi-database studies, published in English language between 2000-2018, and which included at least two different primary care EHR databases or data sources, irrespective of whether other types of database were also included. Primary care EHR data was defined as data collected by primary care clinicians and related staff for the purpose of diagnosis,

For peer review only

treatment, management, and delivery of care of individual patients, and could include information contributed by other care providers.[5] It excluded data generated primarily for administrative purposes such as health insurance claims data, where the motivation for recording is different. Apart from the specific focus on primary care EHR databases, no other restrictions were applied in terms of populations, geography, study period, exposure, outcome or study design.

A previous systematic review highlighted the challenge of identifying multi-database studies, for which no specific MeSH terms exist.[4] An alternative approach was therefore used, based on a comprehensive list of named primary care EHR databases compiled from two online registers[6,7] and one systematic review of primary care data collection projects.[8] For each named database a keyword search was generated and run on Medline, and the results combined. Abstracts of published studies identified in this search were scanned for additional terms and phrases which might be used to describe the primary care EHR data sources, and from these additional keyword searches were generated. The final search strategy [see Supplementary materials] was used to identify studies in Medline and Embase databases on 29 May 2018.

Titles and abstracts of retrieved studies were screened for eligibility by one reviewer (DD), with a random 20% sample also screened by the second reviewer (MC). Reference lists of papers selected for full review were hand searched for additional studies.

Full text was obtained for all papers selected during the initial screening, and read by two reviewers (DD, MC), who independently completed the final eligibility assessment and data extraction. Each reviewer extracted standardised information from the study publication and online supplementary materials (where available), which was entered into the review database (MS Access) via electronic data collection form developed by one of the reviewers (DD), and pilot tested with 7 studies. Information extracted included data sources used, main objectives, study design, study populations, exposure, comparators and outcomes, data management arrangements, and statistical methods. All discordant results were reviewed, and the final designation agreed by both reviewers. No additional information was sought from investigators.

Studies were classified as analytical if they estimated an exposure-outcome association, or descriptive otherwise. We noted whether and how between-database heterogeneity was assessed, how this informed the decision to combine the data, and whether a 1-stage meta-analysis of pooled individual patient data (IPD) or 2-stage meta-analysis of study specific effect estimates was used, as well as choice of fixed-effects (FE) vs random-effect (RE) models. A clear rationale for using multiple data sources was not always stated, but in some instances could be inferred. For analytical studies where results were combined using 1- or 2-stage meta-analysis, unless stated otherwise the rationale was assumed to be an increase in the statistical precision of the exposure-outcome effect estimate. Three main models for data management and analysis were considered, based on previous reviews:[4,9–11] a fully centralised model for management and analysis of the raw data provided by each contributing database; a fully distributed model where all data

For peer review only

management and analysis was undertaken locally, and only fully aggregated results were shared; and a partially distributed model with local extraction and data management to generate standardised patient level or partially aggregated datasets in standardised format, which were then shared for final centralised analysis. We noted whether studies used a global common data model (CDM) such as OMOP[12,13] or Sentinel,[14,15] and whether they were part of a wider program or initiative for developing database networks and methods for combining results – such as IMI-PROTECT,[16,17] EU-ADR,[18] or ARITMO.[19,20]

The focus of the review was on describing the range of multi-database studies and methods for combining primary care EHR data, rather than evaluating evidence of effectiveness of specific interventions. Given this, and in the absence of consensus or validated reporting guidelines for multi-database studies, no formal assessment of risk of bias or study quality was attempted.

The study protocol, including the final Medline search strategy and details of data items extracted is provided in ***Supplementary Appendix I***.

Patient and public involvement

Patients or the public were not involved in the design, or conduct, or reporting, or dissemination plans of our research.

Results

The Medline and EMBASE searches returned 6049 results, and a further 5 were identified by hand searches. After initial screening of abstracts, 138 papers were selected for full text review, and 109 were included in the final review (***Figure 1***). Summary information on the included studies is provided in ***Supplementary Table S1***.

Included studies used data from 38 different primary care EHR databases. Most studies (98, 89%) used 2 or 3 primary care EHR databases, and 43 studies (39%) also included one or more non-primary care EHR database. All but 3 studies used exclusively European primary care data, and 35 (32%) used data from a single country. Details of the primary care EHR databases used in included studies are summarised in ***Supplementary Table S2***.

The annual number of published studies increased over time (***Supplementary Figure S1***), with fewer than 10 studies per year between 2003 – 2013, while a peak in 2016 (25 studies) included 10 studies published in a special supplement on the IMI-PROTECT research program [16,17].

General characteristics of included studies are shown in **Table 1**. More than half (62, or 57%) were classified as analytical. Most studies (76, 70%) examined safety, effectiveness or utilisation of specific drugs accounted for, while 21% (23 studies) were disease epidemiology or risk prediction studies with no specific focus on pharmacological therapies.

For peer review only

Table 1: General characteristics of included studies, including objective, rationale and study design

	Study Type				
	Analytical	Descriptive	Other(a)	**All**	**%**
**All Studies**	**62**	**45**	**2**	**109**	
Study Objective					
Drug safety	37	5		**42**	**38.5**
Drug utilization	3	24	1	**28**	**25.7**
Disease epidemiology	7	9		**16**	**14.7**
Disease risk prediction	5	2		**7**	**6.4**
Drug comparative effectiveness	6			**6**	**5.5**
Methodology / data quality	2	3	1	**6**	**5.5**
Health services research	2	2		**4**	**3.7**
Main rationale for using multiple data sources (stated or inferred)					
Describe trends and variation between countries or settings	2	28		**30**	**27.5**
Increase study power	24	2		**26**	**23.9**
Examine consistency of findings in different settings (using a standardised approach or common study protocol)	19	3		**22**	**20.2**
Compare availability/quality of data in each source	2	7	2	**11**	**10.1**
Validation of findings in a second data source	5	3		**8**	**7.3**
Not clearly stated	10	2		**12**	**11.0**
Databases per study: Primary care EHR only					
Mean	2.4	2.9	2.5	**2.6**	
Median [range]	2 [2-4]	3 [2-5]	2.5 [2-3]	**2 [2-5]**	
Databases per study: all types					
Mean	3.1	4.3	5	**3.7**	
Median [range]	2 [2-8]	4 [2-8]	5 [2-8]	**3 [2-8]**	
Database setting					
Single country	25	9	1	**35**	**31.8**
Multi-country	37	36	1	**75**	**68.2**
Study design(b)					
Cohort study	33	38	1	**72**	**66.1**
Case-control study	23	0	0	**23**	**21.1**
Cross-sectional	1	6	1	**8**	**7.3**
Self controlled designs	7	0	0	**7**	**6.4**
Other	0	1	1	**2**	**1.8**
Interrupted time series	1	0	0	**1**	**0.9**
Data management and analysis model (stated or inferred)					
Centralised management and analysis: raw data shared	32	11	1	**44**	**40.4**
Distributed management and analysis: aggregated results shared	11	12		**23**	**21.1**
Distributed management + centralised analysis: patient level or partially aggregated data shared	11	8	1	**20**	**18.3**
Not described	8	14		**22**	**20.2**
Study drug (ATC Chapter)					
Nervous system (N)	11	8		**19**	**17.4**
Respiratory system (R)	9	5		**14**	**12.8**
Musculo-skeletal system (M)	9	3		**12**	**11.0**
Multiple categories	7	4		**11**	**10.1**
Alimentary tract and metabolism (A)	6	3	1	**10**	**9.2**
Antiinfectives for systemic use (J)	4	4		**8**	**7.3**

For peer review only

Cardiovascular system (C)	2	2		**4**	**3.7**
Genito urinary system and sex hormones (G)	2	1		**3**	**2.8**
Blood and blood forming organs (B)		2		**2**	**1.8**
Dermatologicals (D)		1		**1**	**0.9**
N/A	12	12	1	25	**22.9**
Study condition (ICD-10 chapter)					
Diseases of the circulatory system (I00-I99)	15	4		**19**	**17.4**
Diseases of the respiratory system (J00-J99)	11	4		**15**	**13.8**
Diseases of the digestive system (K00-K95)	9	4		**13**	**11.9**
Multiple categories	3	7		**10**	**9.2**
Endocrine, nutritional and metabolic diseases (E00-E89)	4	3	2	**9**	**8.3**
Injury, poisoning and certain other consequences of external causes (S00-T88)	6	1		**7**	**6.4**
Neoplasms (C00-D49)	5	1		**6**	**5.5**
Diseases of the musculoskeletal system and connective tissue (M00-M99)	4	1		**5**	**4.6**
Diseases of the nervous system (G00-G99)	2	3		**5**	**4.6**
Pregnancy, childbirth and the puerperium (O00-O9A)	1	3		**4**	**3.7**
Certain infectious and parasitic diseases (A00-B99)		2		**2**	**1.8**
Diseases of the skin and subcutaneous tissue (L00-L99)		2		**2**	**1.8**
Diseases of the blood and blood-forming organs and certain disorders involving the immune mechanism (D50-D89)		1		**1**	**0.9**
Diseases of the genitourinary system (N00-N99)	1			**1**	**0.9**
Health status inc. morbidity and/or mortality	1			**1**	**0.9**
Mental, Behavioral and Neurodevelopmental disorders (F01-F99)		1		**1**	**0.9**
N/A		8		**8**	**7.3**

(a) Other study type category included: case definition validation by chart review [1 study]; prescribing data quality assessment [1 study].

(b) Six studies included multiple designs and were therefore included each relevant category: case-control and cohort [3 studies]; case-crossover (CCX) and self-controlled case-series (SCCS) [2 studies]; cohort study and self-controlled case-series (SCCS) [1 study].

For peer review only

Cohort studies were the most common study design (72 studies, 66%), and the majority of these were descriptive. Six studies included more than 1 study design.

The most common approach for data management and analysis was a fully centralised model (44 studies, 40%). A fully distributed model was used in 23 studies (21%), including 15 studies conducted as part of the IMI-PROTECT program[16,21]. A partially distributed approach was used in 20 studies (18%), including 9 studies from the EU-ADR program,[18] and 5 from the ARITMO project.[19,20]. No studies reported using a common data model.

Methodological aspects of the 62 analytical studies are summarised in **Table 2**. All 23 case-control studies employed individual matching, and used conditional logistic regression to estimate adjusted odds ratios, but for cohort studies a range of statistical approaches was used.

For peer review only

Table 2: Methodological aspects of analytical studies (N=62)

Characteristic	Study design(a)					
	Case-control studies	Cohort studies	Self-controlled studies	Other(b)	All	%
All studies	**23**	**34**	**7**	**2**	**62**	
Statistical Methods(c)						
Logistic regression	23	8	2	1	**34**	**54.8**
Poisson regression		6	6		**12**	**19.4**
Cox regression		18			**18**	**29.0**
Other(d)		9	1	1	**11**	**17.7**
Confounder control(c)						
Multiple regression or Mantel Haenszel	23	32		2	**55**	**88.7**
Matching	23	9		1	**29**	**46.8**
Case only / Self-controlled design			7		**7**	**11.3**
Propensity scores		3			**3**	**4.8**
Instrumental variables		2			**2**	**3.2**
None		1			**1**	**1.6**
Database comparisons/heterogeneity assessment (c)						
Participant characteristics presented for each database	17	24	4	2	**45**	**72.6**
Effect estimates presented for each database	18	19	5	2	**41**	**66.1**
Formal test of effect heterogeneity	10	4	3		**17**	**27.4**
- I2	*(6)*	*(3)*	*(3)*		*(12)*	*(19.4)*
- Cochran's Q	*(2)*	*(1)*			*(3)*	*(4.8)*
- (other or not specified)	*(3)*				*(3)*	*(4.8)*
No database comparisons (combined effect estimates only)	5	11	2		**17**	**27.4**
Method for combining data or results(c)						
Data not combined	3	16	3	2	**22**	**35.5**
Meta-analysis (2-stage):	15	4	3		**21**	**33.9**
- random effects (RE)	*(10)*	*(2)*	*(2)*		*(13)*	*(21.0)*
- fixed effects (FE)	*(7)*	*(3)*	*(2)*		*(13)*	*(21.0)*
- method not specified		*(1)*			*(1)*	*(1.6)*
Pooled analysis (1-stage):	12	15	3		**29**	**46.8**
Multiple: 1-stage and 2-stage	*(7)*	*(1)*	*(2)*		*(10)*	*(16.1)*

(a) Six studies contributed to multiple categories because they included multiple designs: case-control and cohort [3 studies]; case-crossover (CCX) and self-controlled case-series (SCCS) [2 studies]; cohort study and self-controlled case-series (SCCS) [1 study].
(b) 1 cross sectional, 1 interrupted time series
(c) A single study could be included in more than 1 category.
(d) Other statistical methods included: Negative binomial regression (2); Mantel-Haenszel (2); 2-stage IV models (2); 'data-mining methods' (2); generalised linear models (1); univariate tests (1).

For peer review only

In 22 analytical studies (35%) data were not combined, and all results were presented separately for each database – usually in order to describe and assess the consistency of findings in different populations or settings, using a common study protocol and analysis approach. In the remaining 5 studies, risk prediction models were developed in one primary care EHR database, and validated using a second primary care EHR database from the same country (UK).[22–26]

In 40 analytical studies (65%), including the majority (20/23) of case-control studies, and half (18/34) of cohort studies, some form of pooled- or meta-analysis was undertaken. A 1-stage meta-analysis of pooled individual patient data (IPD) or partially aggregated data was undertaken in 29 studies (47%). In 19 of these studies, no assessment or discussion of between-database heterogeneity was provided, and only 4 studies reported any form of analytical adjustment for the clustered nature of the pooled data - in each case by including database as a covariate in a multiple regression model, with 1 study also including interaction terms between database and covariates.[27] Two-stage meta-analysis of database-specific effect estimates was used in 21 studies (34%), of which 14 presented some discussion or formal assessment of heterogeneity. The choice of FE or RE models was not clearly justified in most studies, though in 4 cases model choice was based on formal tests of heterogeneity (results not shown).

In 41 analytical studies (66%), separate effect estimates were reported for each database. Between database heterogeneity was formally assessed (most commonly using the I^2 statistic) in 17 studies (27%) and was discussed but not formally assessed in a further 6 studies (10%). In 17 studies (27%) – all of which used a 1-stage approach - results were presented for the combined dataset only. Only two of these 17 additionally included a 2-stage meta-analysis and reported heterogeneity statistics.

Ten studies reported comparable results (in the main paper or in supplementary materials) using two or more analytic approaches, such as both 1-stage and 2-stage meta-analysis (9 studies), or 2-stage analysis with both FE and RE models (1 study), summarised in **Figure 2**. In most cases 1-stage and 2-stage meta-analysis gave very similar results. Confidence intervals from RE meta-analysis were in almost every case wider than the corresponding FE or 1-stage model estimates. In one study (review database id:117) point estimates from 1-stage and 2-stage analyses differed appreciably, and RE model confidence intervals were wider, partly because some databases with very small numbers of cases were excluded.[28] In one self-controlled case series (SCCS) study examining risk of upper gastrointestinal bleeding with different drugs alone and in combination, the point estimates from 1-stage model (ignoring clustering), and 2-stage RE effects models were very different (review database id:113a-d), and confidence intervals from the 2-stage models were much wider.[29]

For peer review only

Discussion

This systematic review identified 109 multi-database studies which used data from 2 or more primary care EHR data sources, the great majority of which were from European countries. Just under half were descriptive studies undertaken either to compare patterns and trends in different populations or settings, or to assess comparability of different EHR databases from similar populations. In these descriptive studies, and in a third of analytical studies, there was no attempt to combine results from the different databases. Where data was combined, a 1-stage meta-analysis of pooled individual patient data (IPD) was used more often than 2-stage meta-analysis of database specific results. Reporting of statistical methods for 1-stage analyses in particular was sub-optimal: in all but 4 such studies, adjustment for clustering or effect heterogeneity was either not explored or not reported.

Whether and how to combine data is a key consideration in multi-database studies, and our results are consistent with a previous systematic review which found that 16 out of 22 multi-database pharmacoepidemiology studies used a 1-stage approach to combine the data.[4] The use of one-stage meta-analysis of IPD in systematic reviews has increased over the past 2 decades,[30–32] and has a number of advantages which are relevant for multi-database studies.[33] Firstly, it ensures standardisation of the statistical analysis across all data sources. Secondly it provides maximum flexibility to explore dose response patterns, subgroup analyses, and effect modification, all of which may help to account for heterogeneity between data sources. Thirdly, a one-stage approach can incorporate information from smaller databases with sparse data, even where the database specific effect cannot be reliably estimated due to zero cell counts.[28,34,35] However, one-stage meta-analysis of IPD should properly account for the clustered nature of the data from contributing databases,[27,36–39] since not doing so may introduce bias, especially if there is between study heterogeneity in effect estimates. The results of this review suggest that clustering is largely ignored in multi-database studies using primary care EHR data, and this is consistent with findings from other reviews of one-stage meta-analysis,[30,31] and multi-database pharmacoepidemiology studies.[4,27] Barriers to the adoption of methods that properly account for clustering may include the perceived statistical complexity, lack of options in commonly available statistical software, or because they can be computationally intensive.[27,40]

A third of analytical studies in this review used a 2-stage approach to combine database specific effect estimates to produce a pooled estimate. This approach avoids the need to share potentially sensitive individual patient data and may therefore be the only available option in some instances. It can also take advantage of local expertise and knowledge of each database partner, including optimising the use of available covariate information to control for confounding. In addition, a 2-stage meta-analysis is relatively straightforward to implement and interpret. It is also possible to conduct pre-planned subgroup analyses, or examine dose-response effects using a 2-stage approach, although sparse data may limit this, since databases with zero cell counts in one or both

For peer review only

comparison groups will usually have to be excluded, unless some form of continuity correction is used, which can introduce bias.[41]

A second key consideration for multi-database studies is how to assess and interpret results in the presence of heterogeneity between databases. Several approaches for harmonising analyses of multi-database studies have been used. These include development and adoption of consistent and validated case definitions, use of common protocols and statistical analysis plans, and shared data management and analysis routines, all aiming to reduce external sources of variability in results from different databases.[10,11,16,17,19] Nevertheless, in studies incorporating data from different countries with different population characteristics and healthcare systems, these factors may contribute to real differences in the effect estimates. Even within a single country, different primary care EHR software systems are used, which may introduce heterogeneity in the extracted data.[42–47] With 1-stage analysis, both clustering and heterogeneity can be naturally explored in hierarchical models incorporating fixed and/or random effects, though as already noted the tendency has been to ignore this issue in multi-database studies. For 2-stage meta-analysis, we found that fixed effect (FE) and random effects (RE) models were used equally frequently and model choice, where discussed at all, was generally related to the presence or absence of heterogeneity. Relatively few studies justified the choice based on some formal measure or test for heterogeneity, a practice which has been criticised because such tests often have low power. This is a problem especially when the number of studies or databases is small,[48] and can result in a FE model being used even though heterogeneity is present and a RE model may have been more appropriate.[49] Simulation studies have shown that the I^2 statistic can also be unreliable, either under- or over-estimating heterogeneity in certain circumstances, particular when the number of studies or databases is small.[50,51]

Several countries (Italy, Netherlands, Spain, UK) now have two or more primary care EHR databases, and combining sources from the same country may reduce heterogeneity. In such cases, a fixed effect model may be appropriate, especially where supporting analyses demonstrate substantial similarities in patient characteristics. Of 8 single-country studies identified in this review and using 2-stage meta-analysis, all 8 used fixed effects models[52–59] - despite evidence of substantial heterogeneity in some cases, although two studies did also use random effects models for some analyses.[52,53]

When combining primary care EHR data from different countries or settings, a random effects model might seem most appropriate, since these incorporate uncertainty in effect size when heterogeneity is present, yet reduce to a fixed effect model if there is no heterogeneity.[48] However, when the number of estimates being combined is very small (<5) and heterogeneity is present – a common scenario in multi-database studies – conventional random effect models may perform poorly. Simulation studies show that they can produce confidence intervals which are too narrow, thereby increasing type 1 error rates.[60–63] A number of alternatives to conventional

For peer review only - http://bmjopen.bmj.com/site/about/guidelines.xhtml

random effects models have been proposed which partially address these limitations in some circumstances,[60,63–66] nevertheless several authors have urged caution when interpreting results from meta-analysis of very few, heterogenous studies.[60,66,67] For multi-database studies, it may therefore be helpful to present estimates for both fixed and random effects models – or other alternative models, but always along with the results from individual databases.

Despite the differences outlined above, in studies which combined data using more than one method produced similar estimates in most cases. However, in at least one study, 1- and 2-stage methods yielded large differences in both the point estimates and their precision. This may in part be related to the substantial heterogeneity in the database specific estimates (reported I^2 between 86-98% for the estimates shown), but incomplete reporting of statistical methods limits further interpretation of these results. When the same modelling assumptions are used, 1- and 2-stages approaches are expected to give very similar results if the number of studies combined is relatively large [37]. However, few studies have systematically compared performance of 1- and 2-stage approaches for multi-database studies.[27,68]

Limitations

The search strategy for this review included a list of named primary care EHR databases compiled from publicly available registers. This was to circumvent the poor sensitivity and specificity of conceptual searches based on MeSH terms, as reported in a previous review,[4] and confirmed in the current review. Our approach could have missed some eligible studies - if the abstract only mentioned primary care EHR data sources that were not in our list or did not mention the use of health databases or related terms at all. We would also have missed non-English language and abstract only publications. Nevertheless, we expect the number of missed studies to be small, and any such studies are unlikely to have differed systematically from the included studies in terms of key methodological aspects. A more recent inventory of EHR databases in Europe did not identify any additional primary care databases that were not included in our review.[69] A further limitation was that some subjective interpretation was occasionally necessary to classify aspects of certain studies - for example the rationale for combining, or the methods for managing and analysing data. The use of two reviewers helped to achieve some consistency across the included studies.

In conclusion, we found a growing body of literature reporting on studies using two or more sources of primary care EHR data. These addressed a range of research questions, and in many cases the results were presented separately and not combined. When data was combined, a 1-stage meta-analysis was preferred. One-stage methods offer advantages in terms of analytical flexibility but are only possible where data management and governance arrangements allow for sharing of individual patient data. However, in many studies using 1-stage approaches, the clustered nature of data from multi-database studies was frequently ignored, with unknown impact

For peer review only

for interpretation. Two-stage meta-analysis requires only sharing of aggregated results, but there are known limitations with current 2-stage methods when the number of studies is small, especially when some heterogeneity is expected. Irrespective of whether a 1- or 2-stage approach is used, combined results should be accompanied by results from each data source separately. This information, together with clear and complete reporting on methods used to standardise and analyse the data (including the rationale for these decisions), affords a more considered assessment of potential sources of heterogeneity and greatly aids interpretation of the overall results. **Box 1** summarises key recommendations arising from our review.

For peer review only

Box 1: Recommendations

Recommendation	Rationale
Studies should report clearly on all aspects of study design and conduct which impact on harmonisation of analyses across data sources.	Allows assessment of the relative importance of heterogeneity induced by data management and analysis decisions *vs.* heterogeneity inherent in the data.
Participant characteristics and effect estimates (where applicable) should be reported for each data source.	Assessment of heterogeneity is essential for interpretation, but formal methods for quantifying heterogeneity are inefficient and possibly biased in multi-database settings.
Where 1-stage methods are used, studies should report whether and how analyses accounted for clustering and between database heterogeneity.	Interpretation requires understanding of extent to which heterogeneity might influence study results.
Where 2-stage meta-analysis is used, studies should provide a clear rationale for choice of fixed effect (FE), random effects (RE), or other model.	Interpretation requires understanding of extent to which heterogeneity might influence study results.
Sensitivity analyses should include alternative methods for combining data	Comparing the results of 1-stage vs 2-stage analyses, or fixed vs random effects models provides information about potential impact of modelling assumptions.
Further research is needed to compare performance of 1-stage and 2-stage approaches for multi-database studies	Relatively few studies have specifically addressed meta-analysis for multi-database studies.

Figure and table legends

Figure 1 [Mono Image]: Selection and inclusion of studies for systematic review

Table 1: General study characteristics, including objective, rationale and study design

Table 2: Methodological aspects of analytical studies (N=62)

Figure 2 [Mono Image]: Comparison of relative risk (RR) estimates reported in studies using 2 or more methods to combine data from multiple sources

For peer review only

References

1 Strom BL. Overview of Automated Databases in Pharmacoepidemiology. In: *Textbook of Pharmacoepidemiology*. Chichester, UK: : John Wiley & Sons Ltd 2013. 118–22. doi:10.1002/9781118344828.ch08

2 Hall GC, Sauer B, Bourke A, *et al.* Guidelines for good database selection and use in pharmacoepidemiology research. *Pharmacoepidemiol Drug Saf* 2012;**21**:1–10. doi:10.1002/pds.2229

3 Devoe JE, Gold R, McIntire P, *et al.* Electronic health records vs Medicaid claims: completeness of diabetes preventive care data in community health centers. *Ann Fam Med* 2011;**9**:351–8. doi:https://dx.doi.org/10.1370/afm.1279

4 Bazelier MT, Eriksson I, de Vries F, *et al.* Data management and data analysis techniques in pharmacoepidemiological studies using a pre-planned multi-database approach: a systematic literature review. *Pharmacoepidemiol Drug Saf* 2015;**24**:897–905. doi:10.1002/pds.3828

5 Häyrinen K, Saranto K, Nykänen P. Definition, structure, content, use and impacts of electronic health records: a review of the research literature. *Int J Med Inform* 2008;**77**:291–304. doi:10.1016/j.ijmedinf.2007.09.001

6 ENCePP. ENCePP Resource Database. http://www.encepp.eu/encepp/resourcesDatabase.jsp (accessed 18 Jan 2018).

7 B.R.I.D.G.E-TO-DATA. DGI, LLC. https://www.bridgetodata.org/ (accessed 23 Jan 2018).

8 Gentil M-L, Cuggia M, Fiquet L, *et al.* Factors influencing the development of primary care data collection projects from electronic health records: a systematic review of the literature. *BMC Med Inform Decis Mak* 2017;**17**:139. doi:10.1186/s12911-017-0538-x

9 Brown JS, Holmes JH, Shah K, *et al.* Distributed health data networks: a practical and preferred approach to multi-institutional evaluations of comparative effectiveness, safety, and quality of care. *Med Care* 2010;**48**:S45-51. doi:10.1097/MLR.0b013e3181d9919f

10 Gini R, Schuemie M, Brown J, *et al.* Data Extraction And Management In Networks Of Observational Health Care Databases For Scientific Research: A Comparison Among EU-ADR, OMOP, Mini-Sentinel And MATRICE Strategies. *eGEMs (Generating Evid Methods to Improv patient outcomes)* 2016;**4**:2. doi:10.13063/2327-9214.1189

11 Lai EC-C, Stang P, Yang Y-HK, *et al.* International Multi-database Pharmacoepidemiology: Potentials and Pitfalls. *Curr Epidemiol Reports* 2015;**2**:229–38. doi:10.1007/s40471-015-0059-z

12 Schuemie MJ, Gini R, Coloma PM, *et al.* Replication of the OMOP experiment in Europe:

For peer review only

evaluating methods for risk identification in electronic health record databases. *Drug Saf* 2013;**36 Suppl 1**:S159-69. doi:10.1007/s40264-013-0109-8

13 Overhage JM, Ryan PB, Reich CG, *et al.* Validation of a common data model for active safety surveillance research. *J Am Med Inform Assoc* 2012;**19**:54–60. doi:10.1136/amiajnl-2011-000376

14 Toh S, Reichman ME, Houstoun M, *et al.* Comparative risk for angioedema associated with the use of drugs that target the renin-angiotensin-aldosterone system. *Arch Intern Med* 2012;**172**:1582–9. doi:10.1001/2013.jamainternmed.34

15 Platt R, Carnahan RM, Brown JS, *et al.* The U.S. Food and Drug Administration's Mini-Sentinel program: status and direction. *Pharmacoepidemiol Drug Saf* 2012;**21 Suppl 1**:1–8. doi:10.1002/pds.2343

16 Klungel OH, Kurz X, de Groot MCH, *et al.* Multi-centre, multi-database studies with common protocols: lessons learnt from the IMI PROTECT project. *Pharmacoepidemiol Drug Saf* 2016;**25 Suppl 1**:156–65. doi:10.1002/pds.3968

17 Reynolds RF, Kurz X, de Groot MCH, *et al.* The IMI PROTECT project: purpose, organizational structure, and procedures. *Pharmacoepidemiol Drug Saf* 2016;**25 Suppl 1**:5–10. doi:10.1002/pds.3933

18 Coloma PM, Schuemie MJ, Trifirò G, *et al.* Combining electronic healthcare databases in Europe to allow for large-scale drug safety monitoring: the EU-ADR Project. *Pharmacoepidemiol Drug Saf* 2011;**20**:1–11. doi:10.1002/pds.2053

19 Trifiro G, Coloma PM, Rijnbeek PR, *et al.* Combining multiple healthcare databases for postmarketing drug and vaccine safety surveillance: why and how?. *J Intern Med* 2014;**275**:551–61. doi:10.1111/joim.12159

20 Mor A, Frøslev T, Thomsen RW, *et al.* Antibiotic use varies substantially among adults: a cross-national study from five European Countries in the ARITMO project. *Infection* 2015;**43**:453–72. doi:10.1007/s15010-015-0768-8

21 Abbing-Karahagopian V, Kurz X, de Vries F, *et al.* Bridging differences in outcomes of pharmacoepidemiological studies: design and first results of the PROTECT project. *Curr Clin Pharmacol* 2014;**9**:130–8.http://www.ncbi.nlm.nih.gov/pubmed/24218995

22 Hippisley-Cox J, Coupland C. Individualising the risks of statins in men and women in England and Wales: population-based cohort study. *Heart* 2010;**96**:939–47. doi:10.1136/hrt.2010.199034

23 Hippisley-Cox J, Coupland C. Development and validation of risk prediction equations to estimate future risk of blindness and lower limb amputation in patients with diabetes: cohort study. *BMJ* 2015;**351**:h5441. doi:10.1136/bmj.h5441

For peer review only

24 Clegg A, Bates C, Young J, *et al.* Development and validation of an electronic frailty index using routine primary care electronic health record data. *Age Ageing* 2016;**45**:353–60. doi:10.1093/ageing/afw039

25 Hippisley-Cox J, Coupland C. Predicting the risk of chronic Kidney Disease in men and women in England and Wales: prospective derivation and external validation of the QKidney Scores. *BMC Fam Pract* 2010;**11**:49. doi:10.1186/1471-2296-11-49

26 Hippisley-Cox J, Coupland C. Development and validation of risk prediction equations to estimate future risk of heart failure in patients with diabetes: a prospective cohort study. *BMJ Open* 2015;**5**:e008503. doi:10.1136/bmjopen-2015-008503

27 La Gamba F, Corrao G, Romio S, *et al.* Combining evidence from multiple electronic health care databases: performances of one-stage and two-stage meta-analysis in matched case-control studies. *Pharmacoepidemiol Drug Saf* 2017;**26**:1213–9. doi:10.1002/pds.4280

28 Trifirò G, de Ridder M, Sultana J, *et al.* Use of azithromycin and risk of ventricular arrhythmia. *CMAJ* 2017;**189**:E560–8. doi:10.1503/cmaj.160355

29 Masclee GMC, Valkhoff VE, Coloma PM, *et al.* Risk of upper gastrointestinal bleeding from different drug combinations. *Gastroenterology* 2014;**147**:784-792.e9. doi:10.1053/j.gastro.2014.06.007

30 Simmonds M, Stewart G, Stewart L. A decade of individual participant data meta-analyses: A review of current practice. *Contemp Clin Trials* 2015;**45**:76–83. doi:10.1016/j.cct.2015.06.012

31 Simmonds MC, Higgins JPT, Stewart LA, *et al.* Meta-analysis of individual patient data from randomized trials: a review of methods used in practice. *Clin Trials* 2005;**2**:209–17. doi:10.1191/1740774505cn087oa

32 Riley RD, Lambert PC, Abo-Zaid G. Meta-analysis of individual participant data: rationale, conduct, and reporting. *BMJ* 2010;**340**:c221. doi:10.1136/bmj.c221

33 Thomas D, Radji S, Benedetti A. Systematic review of methods for individual patient data meta- analysis with binary outcomes. *BMC Med Res Methodol* 2014;**14**:79. doi:10.1186/1471-2288-14-79

34 Mokhles MM, Trifirò G, Dieleman JP, *et al.* The risk of new onset heart failure associated with dopamine agonist use in Parkinson's disease. *Pharmacol Res* 2012;**65**:358–64. doi:10.1016/j.phrs.2011.11.009

35 Poluzzi E, Diemberger I, De Ridder M, *et al.* Use of antihistamines and risk of ventricular tachyarrhythmia: a nested case-control study in five European countries from the ARITMO project. *Eur J Clin Pharmacol* 2017;**73**:1499–510. doi:10.1007/s00228-017-2317-0

36 Austin PC, Merlo J. Intermediate and advanced topics in multilevel logistic regression

For peer review only

analysis. *Stat Med* 2017;**36**:3257–77. doi:10.1002/sim.7336

37 Burke DL, Ensor J, Riley RD. Meta-analysis using individual participant data: one-stage and two-stage approaches, and why they may differ. *Stat Med* 2017;**36**:855–75. doi:10.1002/sim.7141

38 Debray TPA, Moons KGM, van Valkenhoef G, *et al.* Get real in individual participant data (IPD) meta-analysis: a review of the methodology. *Res Synth Methods* 2015;**6**:293–309. doi:10.1002/jrsm.1160

39 Debray TPA, Moons KGM, Abo-Zaid GMA, *et al.* Individual participant data meta-analysis for a binary outcome: one-stage or two-stage? *PLoS One* 2013;**8**:e60650. doi:10.1371/journal.pone.0060650

40 Stewart GB, Altman DG, Askie LM, *et al.* Statistical analysis of individual participant data meta-analyses: a comparison of methods and recommendations for practice. *PLoS One* 2012;**7**:e46042. doi:10.1371/journal.pone.0046042

41 Sweeting MJ, Sutton AJ, Lambert PC. What to add to nothing? Use and avoidance of continuity corrections in meta-analysis of sparse data. *Stat Med* 2004;**23**:1351–75. doi:10.1002/sim.1761

42 Kumarapeli P, de Lusignan S. Using the computer in the clinical consultation; setting the stage, reviewing, recording, and taking actions: multi-channel video study. *J Am Med Inform Assoc* 2013;**20**:e67-75. doi:10.1136/amiajnl-2012-001081

43 De Clercq E, Van Casteren V, Jonckheer P, *et al.* Electronic Patient Record data as proxy of GPs' thoughts. *Stud Health Technol Inform* 2008;**141**:103–10. doi:10.3233/978-1-58603-922-6-103

44 Tai TW, Anandarajah S, Dhoul N, *et al.* Variation in clinical coding lists in UK general practice: a barrier to consistent data entry? *Inform Prim Care* 2007;**15**:143–50. doi:10.14236/jhi.v15i3.652

45 De Wilde S, Carey IM, Bremner SA, *et al.* A comparison of the recording of 30 common childhood conditions in the Doctor's Independent Network and General Practice Research Databases. *Heal Stat Q* 2004;:21–31.http://www.ncbi.nlm.nih.gov/pubmed/15704391

46 Sollie A, Roskam J, Sijmons RH, *et al.* Do GPs know their patients with cancer? Assessing the quality of cancer registration in Dutch primary care: a cross-sectional validation study. *BMJ Open* 2016;**6**:e012669. doi:10.1136/bmjopen-2016-012669

47 Carey IM, Cook DG, De Wilde S, *et al.* Implications of the problem orientated medical record (POMR) for research using electronic GP databases: a comparison of the Doctors Independent Network Database (DIN) and the General Practice Research Database (GPRD). *BMC Fam Pract* 2003;**4**:14. doi:10.1186/1471-2296-4-14

For peer review only

48 Borenstein M, Hedges L V, Higgins JPT, *et al.* A basic introduction to fixed-effect and random-effects models for meta-analysis. *Res Synth Methods* 2010;**1**:97–111. doi:10.1002/jrsm.12

49 Kontopantelis E, Springate DA, Reeves D. A re-analysis of the Cochrane Library data: the dangers of unobserved heterogeneity in meta-analyses. *PLoS One* 2013;**8**:e69930. doi:10.1371/journal.pone.0069930

50 Thorlund K, Imberger G, Johnston BC, *et al.* Evolution of heterogeneity (I2) estimates and their 95% confidence intervals in large meta-analyses. *PLoS One* 2012;**7**:e39471. doi:10.1371/journal.pone.0039471

51 von Hippel PT. The heterogeneity statistic I(2) can be biased in small meta-analyses. *BMC Med Res Methodol* 2015;**15**:35. doi:10.1186/s12874-015-0024-z

52 Vinogradova Y, Coupland C, Hippisley-Cox J. Use of combined oral contraceptives and risk of venous thromboembolism: nested case-control studies using the QResearch and CPRD databases. *BMJ* 2015;**350**:h2135. doi:10.1136/bmj.h2135

53 Thomas SL, Minassian C, Ganesan V, *et al.* Chickenpox and risk of stroke: A self-controlled case series analysis. *Clin Infect Dis* 2014;**58**:61–8. doi:10.1093/cid/cit659

54 Vinogradova Y, Coupland C, Hippisley-Cox J. Exposure to bisphosphonates and risk of common non-gastrointestinal cancers: series of nested case-control studies using two primary-care databases. *Br J Cancer* 2013;**109**:795–806. doi:10.1038/bjc.2013.383

55 Vinogradova Y, Coupland C, Hippisley-Cox J. Exposure to bisphosphonates and risk of gastrointestinal cancers: series of nested case-control studies with QResearch and CPRD data. *BMJ* 2013;**346**:f114. doi:10.1136/bmj.f114

56 Bremner SA, Carey IM, DeWilde S, *et al.* Infections presenting for clinical care in early life and later risk of hay fever in two UK birth cohorts. *Allergy* 2008;**63**:274–83. doi:10.1111/j.1398-9995.2007.01599.x

57 Bremner SA, Carey IM, DeWilde S, *et al.* Early-life exposure to antibacterials and the subsequent development of hayfever in childhood in the UK: case-control studies using the General Practice Research Database and the Doctors' Independent Network. *Clin Exp Allergy* 2003;**33**:1518–25. doi:10.1046/j.1365-2222.2003.01794.x

58 Bremner SA, Carey IM, DeWilde S, *et al.* Timing of routine immunisations and subsequent hay fever risk. *Arch Dis Child* 2005;**90**:567–73. doi:10.1136/adc.2004.051714

59 Bremner SA, Carey IM, DeWilde S, *et al.* Vaccinations, infections and antibacterials in the first grass pollen season of life and risk of later hayfever. *Clin Exp Allergy* 2007;**37**:512–7. doi:10.1111/j.1365-2222.2007.02697.x

For peer review only

60 Gonnermann A, Framke T, Großhennig A, *et al.* No solution yet for combining two independent studies in the presence of heterogeneity. *Stat Med* 2015;**34**:2476–80. doi:10.1002/sim.6473

61 Röver C, Knapp G, Friede T. Hartung-Knapp-Sidik-Jonkman approach and its modification for random-effects meta-analysis with few studies. *BMC Med Res Methodol* 2015;**15**:99. doi:10.1186/s12874-015-0091-1

62 IntHout J, Ioannidis JPA, Borm GF. The Hartung-Knapp-Sidik-Jonkman method for random effects meta-analysis is straightforward and considerably outperforms the standard DerSimonian-Laird method. *BMC Med Res Methodol* 2014;**14**:25. doi:10.1186/1471-2288-14-25

63 Friede T, Röver C, Wandel S, *et al.* Meta-analysis of two studies in the presence of heterogeneity with applications in rare diseases. *Biom J* 2017;**59**:658–71. doi:10.1002/bimj.201500236

64 Doi SAR, Barendregt JJ, Khan S, *et al.* Advances in the meta-analysis of heterogeneous clinical trials I: The inverse variance heterogeneity model. *Contemp Clin Trials* 2015;**45**:130–8. doi:10.1016/j.cct.2015.05.009

65 Stanley TD, Doucouliagos H. Neither fixed nor random: weighted least squares meta-regression. *Res Synth Methods* 2017;**8**:19–42. doi:10.1002/jrsm.1211

66 Seide SE, Röver C, Friede T. Likelihood-based random-effects meta-analysis with few studies: empirical and simulation studies. *BMC Med Res Methodol* 2019;**19**:16. doi:10.1186/s12874-018-0618-3

67 Friede T, Röver C, Wandel S, *et al.* Meta-analysis of two studies in the presence of heterogeneity with applications in rare diseases. *Biom J* 2017;**59**:658–71. doi:10.1002/bimj.201500236

68 Selmer R, Haglund B, Furu K, *et al.* Individual-based versus aggregate meta-analysis in multi-database studies of pregnancy outcomes: the Nordic example of selective serotonin reuptake inhibitors and venlafaxine in pregnancy. *Pharmacoepidemiol Drug Saf* 2016;**25**:1160–9. doi:10.1002/pds.4033

69 Pacurariu A, Plueschke K, McGettigan P, *et al.* Electronic healthcare databases in Europe: descriptive analysis of characteristics and potential for use in medicines regulation. *BMJ Open* 2018;**8**:e023090. doi:10.1136/bmjopen-2018-023090

For peer review only

Footnotes

Author statement

DD: study concept and design, including search strategy; data extraction proformas and study database design; screening of titles and abstracts, and data extraction; analysis; interpretation of results; drafting of manuscript.

MC: screening of titles and abstracts, and data extraction; interpretation of results, critical review of manuscript, and approval of final version.

RW: study concept and design, including search strategy; interpretation of results, critical review of manuscript, and approval of final version.

SE: interpretation of results, critical review of manuscript, and approval of final version.

KB: study concept and design, including search strategy; interpretation of results, critical review of manuscript, and approval of final version.

ID: study concept and design, including search strategy; interpretation of results, critical review of manuscript, and approval of final version.

Acknowledgements

We would like to thank Russell Burke (Information Services, London School of Hygiene and Tropical Medicine) for his advice on bibliographic search strategies.

Funding

This research was conducted as part of a postgraduate doctoral degree funded by the Clinical Practice Research Datalink. KB holds a Sir Henry Dale Fellowship funded by Wellcome and the Royal Society (grant number 107731/Z/15/Z).

Competing interests

None

Ethics approval

Not required

Patient consent for publication

Not required

Data sharing statement

The study database and data-extraction proformas are available on request from DD.

For peer review only

Figure 1: Selection and inclusion of studies for systematic review

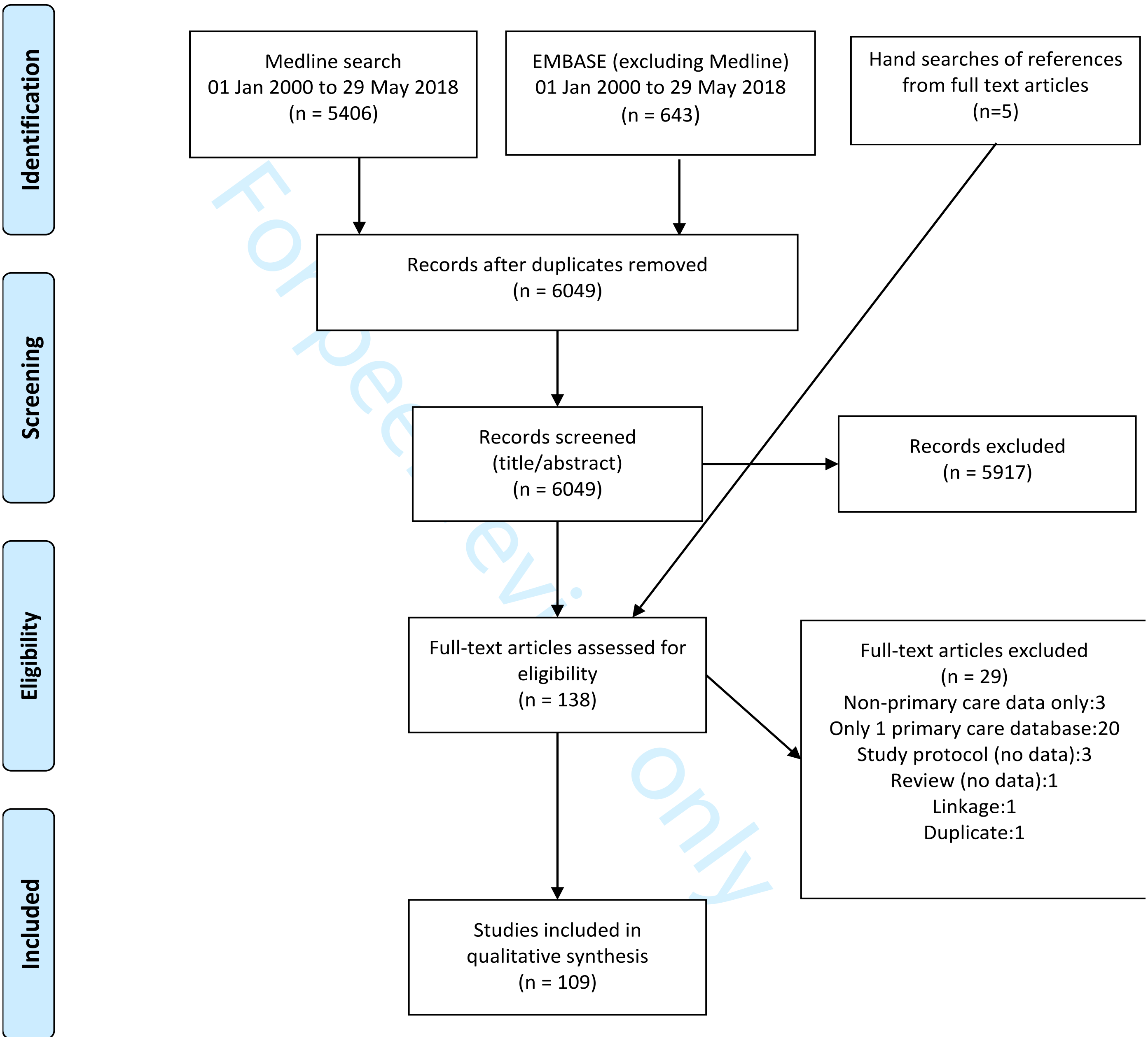

Adapted from: Moher D, Liberati A, Tetzlaff J, Altman DG, The PRISMA Group (2009). *P*referred *R*eporting *I*tems for *S*ystematic Reviews and *M*eta-*A*nalyses: The PRISMA Statement. PLoS Med 6(7): e1000097. doi:10.1371/journal.pmed1000097

For peer review only

Figure 2: Comparison of relative risk (RR) estimates reported in studies using 2 or more methods to combine data from multiple sources

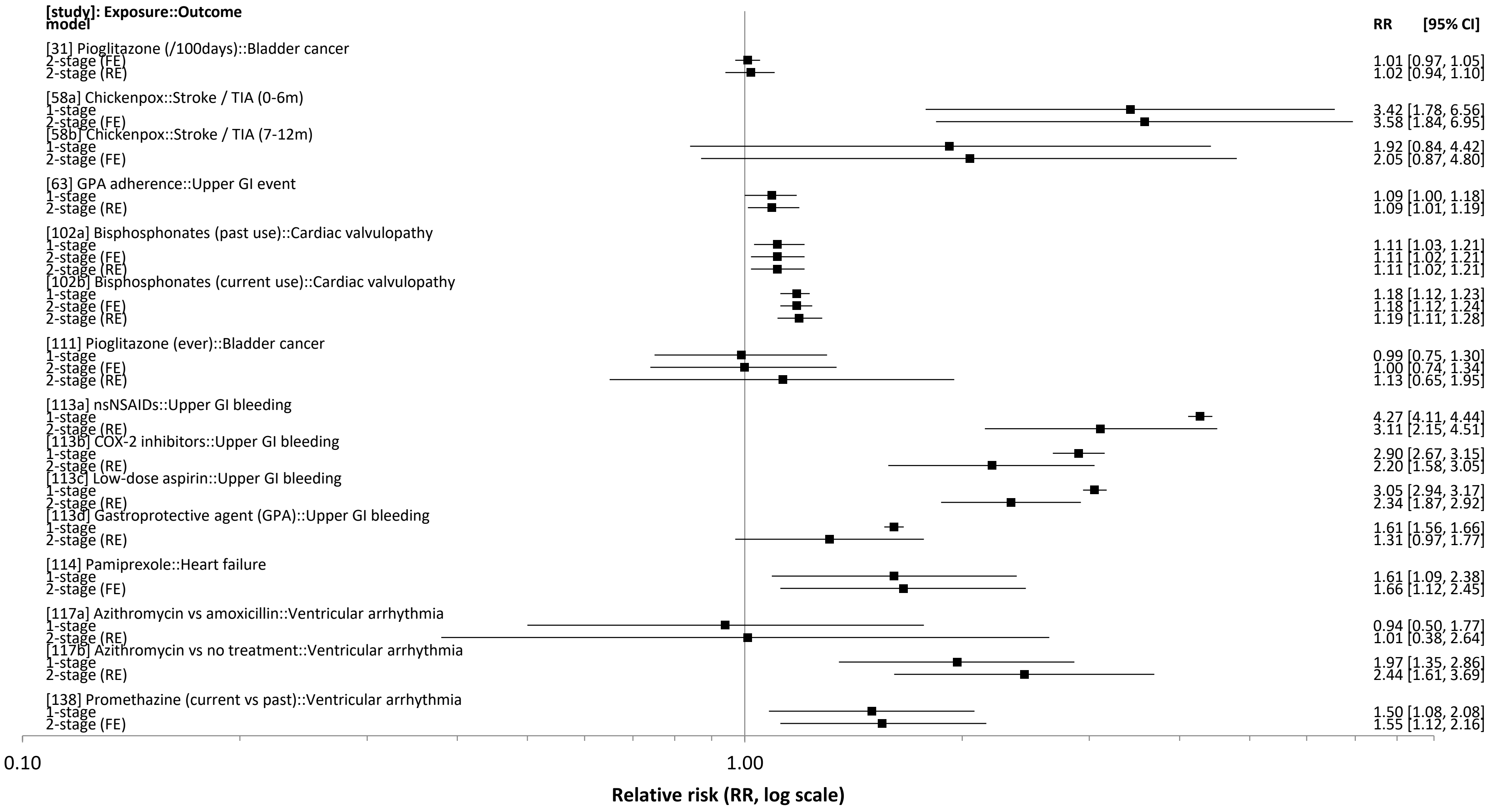

[study] refers to the review database id number (see Supplementary table S1)

Multidatabase Systematic Review: Supplementary Tables and Figures

Figure S1: Number of included studies by year of publication

Table S1: Summary details of 109 studies included in systematic review

Table S2: Summary details of primary care electronic health record (EHR) data sources used in studies included in systematic review

For peer review only

Figure S1: Number of included studies by year of publication

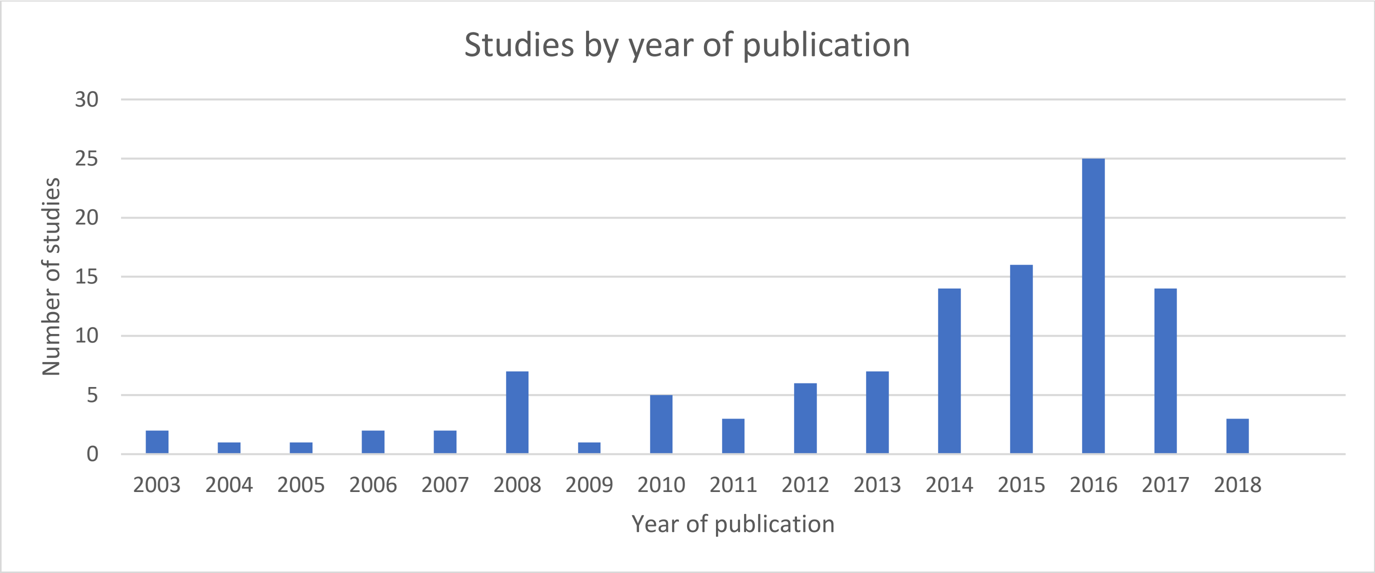

peer review only

Table S1: Summary details of 109 studies included in systematic review.

Study ID[a]	Citation	Resource locator: [doi unless specified otherwise][b]	Study topic	Study type	Study design[c]	Data combining	Primary care EHR data sources[d]	Other sources (if applicable)
1	Lum KJ, Newcomb CW, Roy JA, et al. Evaluation of methods to estimate missing days' supply within pharmacy data of the Clinical Practice Research Datalink (CPRD) and The Health Improvement Network (THIN). Eur J Clin Pharmacol 2017;73:115–23	10.1007/s00228-016-2148-4	Drug utilization	Other	Other	Data not combined	2: CPRD; THIN	
2	de Bie S, Kaguelidou F, Verhamme KMC, et al. Using Prescription Patterns in Primary Care to Derive New Quality Indicators for Childhood Community Antibiotic Prescribing. Pediatr Infect Dis J 2016;35:1317–23	10.1097/INF.0000000000001324	Drug utilization	Descriptive	Cohort	Data not combined	3: IPCI; Pedianet; THIN	
3	Masclee GMC, Coloma PM, Spaander MCW, et al. NSAIDs, statins, low-dose aspirin and PPIs, and the risk of oesophageal adenocarcinoma among patients with Barrett's oesophagus: a population-based Case-control. BMJ Open 2015;5:e006640	10.1136/bmjopen-2014-006640	Drug safety	Analytical	Case-control	Pooled analysis: individual patient data	2: IPCI; THIN	
7	Laforest L, Licaj I, Devouassoux G, et al. Prescribed therapy for asthma: therapeutic ratios and outcomes. BMC Fam Pract 2015;16:49	10.1186/s12875-015-0265-2	Drug comparative effectiveness	Analytical	Cohort	Data not combined	2: LPD (France); THIN	
8	Afonso A, Schmiedl S, Becker C, et al. A methodological comparison of two European primary care databases and replication in a US claims database: inhaled long-acting beta-2-agonists and the risk of acute myocardial infarction. Eur J Clin Pharmacol 2016;72:1105–16	10.1007/s00228-016-2071-8	Drug safety	Analytical	Cohort	Data not combined	2: CPRD; NPCRD (Mondriaan)	1
9	Ali MS, Groenwold RHH, Belitser S V, et al. Methodological comparison of marginal structural model, time-varying Cox regression, and propensity score methods: the example of antidepressant use and the risk of hip fracture. Pharmacoepidemiol Drug Saf 2016;25 Suppl 1:114–21	10.1002/pds.3864	Drug safety	Analytical	Cohort	Data not combined	3: AHC (Mondriaan); BIFAP; NPCRD (Mondriaan)	
10	Bezemer ID, Verhamme KMC, Gini R, et al. Use of oral contraceptives in three European countries: a population-based multi-database study. Eur J Contracept Reprod Health Care 2016;21:81–7	10.3109/13625187.2015.1102220	Drug utilization	Descriptive	Cohort	Data not combined	3: HSD (Italy); IPCI; THIN	1
11	Brauer R, Douglas I, Garcia Rodriguez LA, et al. Risk of acute liver injury associated with use of antibiotics. Comparative cohort and nested case-control studies using two primary care databases in Europe. Pharmacoepidemiol Drug Saf 2016;25 Suppl 1:29–38	10.1002/pds.3861	Drug safety	Analytical	Multiple: case-control; cohort	Data not combined	2: BIFAP; CPRD	
12	Brauer R, Ruigómez A, Downey G, et al. Prevalence of antibiotic use: a comparison across various European health care data sources. Pharmacoepidemiol Drug Saf 2016;25 Suppl 1:11–20	10.1002/pds.3831	Drug utilization	Descriptive	Cross-sectional	Data not combined	5: AHC (Mondriaan); BIFAP; CPRD; NPCRD (Mondriaan); THIN	2
14	Castellsague J, Perez-Gutthann S, Calingaert B, et al. Characterization of new users of cilostazol in the UK, Spain, Sweden, and Germany. Pharmacoepidemiol Drug Saf 2017;26:615–24	10.1002/pds.4167	Drug utilization	Descriptive	Cohort	Data not combined	2: SIDIAP; THIN	3
15	Charlton RA, Pierini A, Klungsøyr K, et al. Asthma medication prescribing before, during and after pregnancy: a study in seven European regions. BMJ Open 2016;6:e009237	10.1136/bmjopen-2015-009237	Drug utilization	Descriptive	Cohort	Data not combined	2: CPRD; SAIL	5
16	Charlton R, Garne E, Wang H, et al. Antiepileptic drug prescribing before, during and after pregnancy: a study in seven European regions. Pharmacoepidemiol Drug Saf 2015;24:1144–54	10.1002/pds.3847	Drug utilization	Descriptive	Cohort	Data not combined	2: CPRD; SAIL	5
17	Chui CSL, Chan EW, Wong AYS, et al. Association between oral fluoroquinolones and seizures: A self-controlled case series study. Neurology 2016;86:1708–15	10.1212/WNL.0000000000002633	Drug safety	Analytical	SCCS	Meta-analysis: random effects	2: CDARS; CPRD	
18	Clegg A, Bates C, Young J, et al. Development and validation of an electronic frailty index using routine primary care electronic health record data. Age Ageing 2016;45:353–60	10.1093/ageing/afw039	Disease risk prediction	Analytical	Cohort	Data not combined	2: ResearchOne; THIN	
19	De Bortoli N, Ripellino C, Cataldo N, et al. Unspecified intestinal malabsorption in patients treated with angiotensin-converting enzyme inhibitors or angiotensin receptor blockers: a retrospective analysis in primary care settings. Expert Opin Drug Saf 2017;16:1221–5	10.1080/14740338.2017.1376647	Drug safety	Descriptive	Cohort	Pooled analysis: individual patient data	2: Disease Analyzer (Germany); HSD (Italy)	

For peer review only

Study ID[a]	Citation	Resource locator: [doi unless specified otherwise][b]	Study topic	Study type	Study design[c]	Data combining	Primary care EHR data sources[d]	Other sources (if applicable)
20	de Groot MCH, Candore G, Uddin MJ, et al. Case-only designs for studying the association of antidepressants and hip or femur fracture. Pharmacoepidemiol Drug Saf 2016;25 Suppl 1:103–13	10.1002/pds.3850	Drug safety	Analytical	Multiple: CCX; SCCS	Data not combined	3: AHC (Mondriaan); NPCRD (Mondriaan); THIN	
24	Ferrajolo C, Verhamme KMC, Trifirò G, et al. Antibiotic-Induced Liver Injury in Paediatric Outpatients: A Case-control in Primary Care Databases. Drug Saf 2017;40:305–15	10.1007/s40264-016-0493-y	Drug safety	Analytical	Case-control	Pooled analysis: individual patient data	3: HSD (Italy); IPCI; Pedianet	
25	Gold R, Esterberg E, Hollombe C, et al. Low Back Imaging When Not Indicated: A Descriptive Cross-System Analysis. Perm J 2016;20:25–33	10.7812/TPP/15-081	Health services research	Analytical	Cohort	Pooled analysis: individual patient data	2: KP Clarity (Epic EHR); OCHIN Clarity (Epic EHR)	
26	Hippisley-Cox J, Coupland C. Development and validation of risk prediction equations to estimate future risk of blindness and lower limb amputation in patients with diabetes: Cohort. BMJ 2015;351:h5441	10.1136/bmj.h5441	Disease risk prediction	Analytical	Cohort	Data not combined	2: CPRD; QResearch	
27	Hippisley-Cox J, Coupland C. Development and validation of risk prediction equations to estimate future risk of heart failure in patients with diabetes: a prospective Cohort. BMJ Open 2015;5:e008503	10.1136/bmjopen-2015-008503	Disease risk prediction	Analytical	Cohort	Data not combined	2: CPRD; QResearch	
28	Israel E, Roche N, Martin RJ, et al. Increased Dose of Inhaled Corticosteroid versus Add-On Long-acting ß-Agonist for Step-Up Therapy in Asthma. Ann Am Thorac Soc 2015;12:798–806	10.1513/AnnalsATS.201412-580OC	Drug comparative effectiveness	Analytical	Cohort	Pooled analysis: individual patient data	2: CPRD; OPCRD	
30	Levi M, Rosselli M, Simonetti M, et al. Epidemiology of iron deficiency anaemia in four European countries: a population-based study in primary care. Eur J Haematol 2016;97:583–93	10.1111/ejh.12776	Disease epidemiology	Descriptive	Cohort	Data not combined	4: HSD (Italy); LPD (Belgium); LPD (Germany); LPD (Spain)	
31	Levin D, Bell S, Sund R, et al. Pioglitazone and bladder cancer risk: a multipopulation pooled, cumulative exposure analysis. Diabetologia 2015;58:493–504	10.1007/s00125-014-3456-9	Drug safety	Analytical	Cohort	Meta-analysis: fixed & random effects	2: CPRD; SIR	4
32	Murray CS, Thomas M, Richardson K, et al. Comparative Effectiveness of Step-up Therapies in Children with Asthma Prescribed Inhaled Corticosteroids: A Historical Cohort. J allergy Clin Immunol Pract 2017;5:1082-1090.e7	10.1016/j.jaip.2016.12.28	Drug comparative effectiveness	Analytical	Cohort	Pooled analysis: individual patient data	2: CPRD; OPCRD	
33	Nyberg F, Horne L, Morlock R, et al. Comorbidity Burden in Trial-Aligned Patients with Established Gout in Germany, UK, US, and France: a Retrospective Analysis. Adv Ther 2016;33:1180–98	10.1007/s12325-016-0346-1	Disease epidemiology	Descriptive	Cohort	Data not combined	3: CPRD; Disease Analyzer (France); Disease Analyzer (Germany)	1
34	Oteri A, Mazzaglia G, Pecchioli S, et al. Prescribing pattern of antipsychotic drugs during the years 1996-2010: a population-based database study in Europe with a focus on torsadogenic drugs. Br J Clin Pharmacol 2016;82:487–97	10.1111/bcp.12955	Drug utilization	Descriptive	Cohort	Data not combined	3: HSD (Italy); IPCI; THIN	4
35	Petersen I, McCrea RL, Sammon CJ, et al. Risks and benefits of psychotropic medication in pregnancy: cohort studies based on UK electronic primary care health records. Health Technol Assess 2016;20:1–176	10.3310/hta20230	Drug safety	Analytical	Cohort	Pooled analysis: individual patient data	2: CPRD; THIN	
36	Petherick ES, Pickett KE, Cullum NA. Can different primary care databases produce comparable estimates of burden of disease: results of a study exploring venous leg ulceration. Fam Pract 2015;32:374–80	10.1093/fampra/cmv013	Disease epidemiology	Descriptive	Cohort	Data not combined	2: CPRD; THIN	
37	Rathmann W, Czech M, Franek E, et al. Regional differences in insulin therapy regimens in five European countries. Int J Clin Pharmacol Ther 2017;55:403–8	10.5414/CP202906	Drug utilization	Descriptive	Cross-sectional	Data not combined	3: Disease Analyzer (France); Disease Analyzer (Germany); Disease Analyzer (UK)	2
38	Requena G, Huerta C, Gardarsdottir H, et al. Hip/femur fractures associated with the use of benzodiazepines (anxiolytics, hypnotics and related drugs): a methodological approach to assess consistencies across databases from the PROTECT-EU project. Pharmacoepidemiol Drug Saf 2016;25 Suppl 1:66–78	10.1002/pds.3816	Drug safety	Analytical	Multiple: case-control; cohort	Meta-analysis: random effects	3: BIFAP; CPRD; NPCRD (Mondriaan)	
39	Requena G, Logie J, Martin E, et al. Do case-only designs yield consistent results across design and different databases? A case study of hip fractures and benzodiazepines. Pharmacoepidemiol Drug Saf 2016;25 Suppl 1:79–87	10.1002/pds.3822	Drug safety	Analytical	Multiple: CCX;SCCS	Data not combined	2: BIFAP; CPRD	

For peer review only - http://bmjopen.bmj.com/site/about/guidelines.xhtml

Study ID[a]	Citation	Resource locator: [doi unless specified otherwise][b]	Study topic	Study type	Study design[c]	Data combining	Primary care EHR data sources[d]	Other sources (if applicable)
40	Saine ME, Carbonari DM, Newcomb CW, et al. Determinants of saxagliptin use among patients with type 2 diabetes mellitus treated with oral anti-diabetic drugs. BMC Pharmacol Toxicol 2015;16:8	10.1186/s40360-015-0007-z	Drug utilization	Analytical	Cross-sectional	Data not combined	2: CPRD; THIN	2
41	Souverein PC, Abbing-Karahagopian V, Martin E, et al. Understanding inconsistency in the results from observational pharmacoepidemiological studies: the case of antidepressant use and risk of hip/femur fractures. Pharmacoepidemiol Drug Saf 2016;25 Suppl 1:88–102	10.1002/pds.3862	Drug safety	Analytical	Multiple: case-control; cohort	Data not combined	4: AHC (Mondriaan); BIFAP; NPCRD (Mondriaan); THIN	
43	Sultana J, Fontana A, Giorgianni F, et al. The Effect of Safety Warnings on Antipsychotic Drug Prescribing in Elderly Persons with Dementia in the United Kingdom and Italy: A Population-Based Study. CNS Drugs 2016;30:1097–109	10.1007/s40263-016-0366-z	Health services research	Analytical	Interrupted time series	Data not combined	2: HSD (Italy); THIN	
44	Turner SW, Richardson K, Burden A, et al. Initial step-up treatment changes in asthmatic children already prescribed inhaled corticosteroids: a historical Cohort. NPJ Prim care Respir Med 2015;25:15041	10.1038/npjpcrm.2015.41	Drug utilization	Descriptive	Cohort	Pooled analysis: individual patient data	2: CPRD; OPCRD	
45	Turner S, Richardson K, Murray C, et al. Long-Acting ß-Agonist in Combination or Separate Inhaler as Step-Up Therapy for Children with Uncontrolled Asthma Receiving Inhaled Corticosteroids. J allergy Clin Immunol Pract 2017;5:99-106.e3	10.1016/j.jaip.2016.06.9	Drug comparative effectiveness	Analytical	Cohort	Pooled analysis: individual patient data	2: CPRD; OPCRD	
46	Tyrstrup M, van der Velden A, Engstrom S, et al. Antibiotic prescribing in relation to diagnoses and consultation rates in Belgium, the Netherlands and Sweden: use of European quality indicators. Scand J Prim Health Care 2017;35:10–8	10.1080/02813432.2017.1288680	Health services research	Descriptive	Cross-sectional	Data not combined	3: Intego; Jonkoping County; Julius (Mondriaan)	
47	Uddin MJ, Groenwold RHH, de Boer A, et al. Evaluating different physician's prescribing preference based instrumental variables in two primary care databases: a study of inhaled long-acting beta2-agonist use and the risk of myocardial infarction. Pharmacoepidemiol Drug Saf 2016;25 Suppl 1:132–41	10.1002/pds.3860	Drug safety	Analytical	Cohort	Pooled analysis: individual patient data	2: CPRD; NPCRD (Mondriaan)	
48	Uddin MJ, Groenwold RHH, de Boer A, et al. Instrumental variables analysis using multiple databases: an example of antidepressant use and risk of hip fracture. Pharmacoepidemiol Drug Saf 2016;25 Suppl 1:122–31	10.1002/pds.3863	Drug safety	Analytical	Cohort	Pooled analysis: individual patient data	3: BIFAP; NPCRD (Mondriaan); THIN	
49	Vinogradova Y, Coupland C, Hippisley-Cox J. Use of combined oral contraceptives and risk of venous thromboembolism: nested case-control studies using the QResearch and CPRD databases. BMJ 2015;350:h2135	10.1136/bmj.h2135	Drug safety	Analytical	Case-control	Meta-analysis: fixed & random effects	2: CPRD; QResearch	
50	Hippisley-Cox J, Coupland C, Brindle P. The performance of seven QPrediction risk scores in an independent external sample of patients from general practice: a validation study. BMJ Open 2014;4:e005809	10.1136/bmjopen-2014-005809	Disease risk prediction	Descriptive	Cohort	Data not combined	2: CPRD; QResearch	
51	Kostev K, Jockwig A, Hallwachs A, et al. Prevalence and risk factors of neuropathy in newly diagnosed type 2 diabetes in primary care practices: a retrospective database analysis in Germany and UK. Prim Care Diabetes 2014;8:250–5	10.1016/j.pcd.2014.01.11	Disease epidemiology	Analytical	Cohort	Data not combined	2: Disease Analyzer (Germany); Disease Analyzer (UK)	
52	Kostev K, Rathmann W. Influence of macro- and microvascular comorbidity on time to insulin initiation in type 2 diabetes patients: a retrospective database analysis in Germany, France, and UK. Prim Care Diabetes 2013;7:167–71	10.1016/j.pcd.2013.02.001	Drug utilization	Analytical	Cohort	Data not combined	3: Disease Analyzer (France); Disease Analyzer (Germany); Disease Analyzer (UK)	
54	Masclee GMC, Coloma PM, de Wilde M, et al. The incidence of Barrett's oesophagus and oesophageal adenocarcinoma in the United Kingdom and The Netherlands is levelling off. Aliment Pharmacol Ther 2014;39:1321–30	10.1111/apt.12759	Disease epidemiology	Descriptive	Cohort	Data not combined	2: IPCI; THIN	
55	Masclee GMC, Valkhoff VE, van Soest EM, et al. Cyclo-oxygenase-2 inhibitors or nonselective NSAIDs plus gastroprotective agents: what to prescribe in daily clinical practice?.Aliment Pharmacol Ther 2013;38:178–89	10.1111/apt.12348	Drug safety	Analytical	Case-control	Multiple: pooled IPD + meta-analysis (RE)	3: CPRD; HSD (Italy); IPCI	
56	de Jonge L, Garne E, Gini R, et al. Improving Information on Maternal Medication Use by Linking Prescription Data to Congenital Anomaly Registers: A EUROmediCAT Study. Drug Saf 2015;38:1083–93	10.1007/s40264-015-0321-9	Disease epidemiology	Descriptive	Cohort	Data not combined	5: AHC (Mondriaan); BIFAP; CPRD; NPCRD (Mondriaan); THIN	2

Study ID[a]	Citation	Resource locator: [doi unless specified otherwise][b]	Study topic	Study type	Study design[c]	Data combining	Primary care EHR data sources[d]	Other sources (if applicable)
57	Ruigómez A, Brauer R, Rodríguez LAG, et al. Ascertainment of acute liver injury in two European primary care databases. Eur J Clin Pharmacol 2014;70:1227–35	10.1007/s00228-014-1721-y	Disease epidemiology	Descriptive	Cohort	Data not combined	2: BIFAP; CPRD	
58	Thomas SL, Minassian C, Ganesan V, et al. Chickenpox and risk of stroke: A self-controlled case series analysis. Clin Infect Dis 2014;58:61–8	10.1093/cid/cit659	Disease epidemiology	Analytical	SCCS	Multiple: pooled IPD + meta-analysis (RE + FE)	4: CPRD; Disease Analyzer (UK); QResearch; THIN	
59	Trifirò G, Mokhles MM, Dieleman JP, et al. Risk of cardiac valve regurgitation with dopamine agonist use in Parkinson's disease and hyperprolactinaemia: a multi-country, nested Case-control. Drug Saf 2012;35:159–71	10.2165/11594940-000000000-00000	Drug safety	Analytical	Case-control	Pooled analysis: individual patient data	3: HSD (Italy); IPCI; THIN	
60	Valkhoff VE, van Soest EM, Masclee GMC, et al. Prescription of nonselective NSAIDs, coxibs and gastroprotective agents in the era of rofecoxib withdrawal - a 617,400-patient study. Aliment Pharmacol Ther 2012;36:790–9	10.1111/apt.12028	Drug utilization	Descriptive	Cohort	Data not combined	3: CPRD; HSD (Italy); IPCI	
61	Valkhoff VE, Coloma PM, Masclee GMC, et al. Validation study in four health-care databases: Upper gastrointestinal bleeding misclassification affects precision but not magnitude of drug-related upper gastrointestinal bleeding risk. J Clin Epidemiol 2014;67:921–31	10.1016/j.jclinepi.2014.02.020	Methodology / data quality	Analytical	Cohort	Pooled analysis: individual patient data	2: HSD (Italy); IPCI	2
62	Valkhoff VE, Schade R, 't Jong GW, et al. Population-based analysis of non-steroidal anti-inflammatory drug use among children in four European countries in the SOS project: what size of data platforms and which study designs do we need to assess safety issues?BMC Pediatr 2013;13:192	10.1186/1471-2431-13-192	Drug utilization	Descriptive	Cohort	Pooled analysis: semi-aggregate data	3: IPCI; Pedianet; THIN	4
63	Valkhoff VE, van Soest EM, Mazzaglia G, et al. Adherence to gastroprotection during cyclooxygenase 2 inhibitor treatment and the risk of upper gastrointestinal tract events: a population-based study. Arthritis Rheum 2012;64:2792–802	10.1002/art.34433	Drug safety	Analytical	Case-control	Multiple: pooled IPD + meta-analysis (RE)	3: CPRD; HSD (Italy); IPCI	
64	Vinogradova Y, Coupland C, Hippisley-Cox J. Exposure to bisphosphonates and risk of common non-gastrointestinal cancers: series of nested case-control studies using two primary-care databases. Br J Cancer 2013;109:795–806	10.1038/bjc.2013.383	Drug safety	Analytical	Case-control	Meta-analysis: fixed effects	2: CPRD; QResearch	
66	Vinogradova Y, Coupland C, Hippisley-Cox J. Exposure to bisphosphonates and risk of gastrointestinal cancers: series of nested case-control studies with QResearch and CPRD data. BMJ 2013;346:f114	10.1136/bmj.f114	Drug safety	Analytical	Case-control	Meta-analysis: fixed effects	2: CPRD; QResearch	
67	Bremner SA, Carey IM, DeWilde S, et al. Infections presenting for clinical care in early life and later risk of hay fever in two UK birth cohorts. Allergy 2008;63:274–83	10.1111/j.1398-9995.2007.01599.x	Disease epidemiology	Analytical	Case-control	Meta-analysis: fixed effects	2: CPRD; DIN	
68	Cooper C, Steinbuch M, Stevenson R, et al. The epidemiology of osteonecrosis: findings from the GPRD and THIN databases in the UK. Osteoporos Int 2010;21:569–77	10.1007/s00198-009-1003-1	Disease epidemiology	Analytical	Case-control	Pooled analysis: individual patient data	2: CPRD; THIN	
69	De Clercq E, Van Casteren V, Jonckheer P, et al. Electronic Patient Record data as proxy of GPs' thoughts. Stud Health Technol Inform 2008;141:103–10	10.3233/978-1-58603-922-6-103	Methodology / data quality	Descriptive	Other	Data not combined	3: Resoprim (x3 networks)	
70	Hippisley-Cox J, Coupland C, Vinogradova Y, et al. Performance of the QRISK cardiovascular risk prediction algorithm in an independent UK sample of patients from general practice: a validation study. Heart 2008;94:34–9	10.1136/hrt.2007.134890	Disease risk prediction	Descriptive	Cohort	Data not combined	2: QResearch; THIN	
71	Hippisley-Cox J, Coupland C. Individualising the risks of statins in men and women in England and Wales: population-based Cohort. Heart 2010;96:939–47	10.1136/hrt.2010.199034	Disease risk prediction	Analytical	Cohort	Data not combined	2: QResearch; THIN	
72	Hippisley-Cox J, Coupland C. Predicting the risk of chronic Kidney Disease in men and women in England and Wales: prospective derivation and external validation of the QKidney Scores. BMC Fam Pract 2010;11:49	10.1186/1471-2296-11-49	Disease risk prediction	Analytical	Cohort	Data not combined	2: QResearch; THIN	
73	Hsia Y, Neubert A, Sturkenboom MCJM, et al. Comparison of antiepileptic drug prescribing in children in three European countries. Epilepsia 2010;51:789–96	10.1111/j.1528-1167.2009.02331.x	Drug utilization	Descriptive	Cohort	Pooled analysis: semi-aggregate data	3: Disease Analyzer (UK); IPCI; Pedianet	
74	Molokhia M, McKeigue P, Curcin V, et al. Statin induced myopathy and myalgia: time trend analysis and comparison of risk associated with statin class from 1991-2006. PLoS One 2008;3:e2522	10.1371/journal.pone.0002522	Drug safety	Analytical	CCX	Data not combined	2: Mediplus; THIN	

For peer review only

Study ID[a]	Citation	Resource locator: [doi unless specified otherwise][b]	Study topic	Study type	Study design[c]	Data combining	Primary care EHR data sources[d]	Other sources (if applicable)
75	Neubert A, Verhamme K, Murray ML, et al. The prescribing of analgesics and non-steroidal anti-inflammatory drugs in paediatric primary care in the UK, Italy and the Netherlands. Pharmacol Res 2010;62:243–8	10.1016/j.phrs.2010.04.006	Drug utilization	Descriptive	Cohort	Data not combined	3: Disease Analyzer (UK); IPCI; Pedianet	
76	Pfeil N, Uhlig U, Kostev K, et al. Antiemetic medications in children with presumed infectious gastroenteritis--pharmacoepidemiology in Europe and Northern America. J Pediatr 2008;153:659–62, 662.e1-3	10.1016/j.jpeds.2008.07.50	Drug utilization	Descriptive	Cohort	Data not combined	3: Disease Analyzer (France); Disease Analyzer (Germany); Disease Analyzer (UK)	2
77	Sturkenboom MCJM, Dieleman JP, Picelli G, et al. Prevalence and treatment of hypertensive patients with multiple concomitant cardiovascular risk factors in The Netherlands and Italy. J Hum Hypertens 2008;22:704–13	10.1038/jhh.2008.82	Disease epidemiology	Descriptive	Cohort	Data not combined	2: HSD (Italy); IPCI	
78	Sturkenboom MCJM, Verhamme KMC, Nicolosi A, et al. Drug use in children: Cohort in three European countries. BMJ 2008;337:a2245–a2245	10.1136/bmj.a2245	Drug utilization	Descriptive	Cohort	Pooled analysis: semi-aggregate data	3: Disease Analyzer (UK); IPCI; Pedianet	
79	van Soest EM, Valkhoff VE, Mazzaglia G, et al. Suboptimal gastroprotective coverage of NSAID use and the risk of upper gastrointestinal bleeding and ulcers: an observational study using three European databases. Gut 2011;60:1650–9	10.1136/gut.2011.239848	Drug safety	Analytical	Case-control	Pooled analysis: individual patient data	3: CPRD; HSD (Italy); IPCI	
80	van Staa TP, Sprafka JM. Study of adverse outcomes in women using testosterone therapy. Maturitas 2009;62:76–80	10.1016/j.maturitas.2008.11.001	Drug safety	Analytical	Cohort	Pooled analysis: individual patient data	2: CPRD; THIN	
81	Brankin E, Walker M, Lynch N, et al. The impact of dosing frequency on compliance and persistence with bisphosphonates among postmenopausal women in the UK: evidence from three databases. Curr Med Res Opin 2006;22:1249–56	10.1185/030079906X112688	Drug utilization	Analytical	Cohort	Data not combined	3: CPRD; DIN; Disease Analyzer (UK)	
82	Bremner SA, Carey IM, DeWilde S, et al. Early-life exposure to antibacterials and the subsequent development of hayfever in childhood in the UK: case-control studies using the General Practice Research Database and the Doctors' Independent Network. Clin Exp Allergy 2003;33:1518–25	10.1046/j.1365-2222.2003.01794.x	Disease epidemiology	Analytical	Case-control	Meta-analysis: fixed effects	2: CPRD; DIN	
83	Bremner SA, Carey IM, DeWilde S, et al. Timing of routine immunisations and subsequent hay fever risk. Arch Dis Child 2005;90:567–73	10.1136/adc.2004.051714	Disease epidemiology	Analytical	Case-control	Meta-analysis: fixed effects	2: CPRD; DIN	
84	Bremner SA, Carey IM, DeWilde S, et al. Vaccinations, infections and antibacterials in the first grass pollen season of life and risk of later hayfever. Clin Exp Allergy 2007;37:512–7	10.1111/j.1365-2222.2007.02697.x	Disease epidemiology	Analytical	Case-control	Meta-analysis: fixed effects	2: CPRD; DIN	
86	Carey IM, Cook DG, De Wilde S, et al. Implications of the problem orientated medical record (POMR) for research using electronic GP databases: a comparison of the Doctors Independent Network Database (DIN) and the General Practice Research Database (GPRD). BMC Fam Pract 2003;4:14	10.1186/1471-2296-4-14	Methodology / data quality	Descriptive	Cohort	Data not combined	2: CPRD; DIN	
88	De Wilde S, Carey IM, Bremner SA, et al. A comparison of the recording of 30 common childhood conditions in the Doctor's Independent Network and General Practice Research Databases. Heal Stat Q 2004;:21–31	pmid:15704391	Methodology / data quality	Descriptive	Cohort	Data not combined	2: CPRD; DIN	
89	Hernández-Díaz S, García Rodríguez LA. Cardioprotective aspirin users and their excess risk of upper gastrointestinal complications. BMC Med 2006;4:22	10.1186/1741-7015-4-22	Drug safety	Descriptive	Cohort	Data not combined	2: BIFAP; CPRD	
90	Jordan K, Clarke AM, Symmons DPM, et al. Measuring disease prevalence: a comparison of musculoskeletal disease using four general practice consultation databases. Br J Gen Pract 2007;57:7–14	pmid:17244418	Disease epidemiology	Descriptive	Cohort	Data not combined	2: CPRD; CiPCA	2
93	Price DB, Gefen E, Gopalan G, et al. Real-life effectiveness and safety of salbutamol Steri-NebTM vs. Ventolin Nebules® for exacerbations in patients with COPD: Historical Cohort. PLoS One 2018;13:e0191404	10.1371/journal.pone.0191404	Drug comparative effectiveness	Analytical	Cohort	Pooled analysis: individual patient data	2: CPRD; OPCRD	
94	Abbing-Karahagopian V, Huerta C, Souverein PC, et al. Antidepressant prescribing in five European countries: application of common definitions to assess the prevalence, clinical observations, and methodological implications. Eur J Clin Pharmacol 2014;70:849–57	10.1007/s00228-014-1676-z	Drug utilization	Descriptive	Cohort	Data not combined	5: AHC (Mondriaan); BIFAP; CPRD; NPCRD (Mondriaan); THIN	2

For peer review only

Study ID[a]	Citation	Resource locator: [doi unless specified otherwise][b]	Study topic	Study type	Study design[c]	Data combining	Primary care EHR data sources[d]	Other sources (if applicable)
101	Charlton RA, Jordan S, Pierini A, et al. Selective serotonin reuptake inhibitor prescribing before, during and after pregnancy: a population-based study in six European regions. BJOG 2015;122:1010–20	10.1111/1471-0528.13143	Drug utilization	Descriptive	Cohort	Data not combined	2: CPRD; SAIL	4
102	Coloma PM, de Ridder M, Bezemer I, et al. Risk of cardiac valvulopathy with use of bisphosphonates: a population-based, multi-country Case-control. Osteoporos Int 2016;27:1857–67	10.1007/s00198-015-3441-2	Drug safety	Analytical	Case-control	Multiple: pooled IPD + meta-analysis (RE + FE)	3: HSD (Italy); IPCI; THIN	3
103	Coloma PM, Schuemie MJ, Trifirò G, et al. Drug-induced acute myocardial infarction: identifying 'prime suspects' from electronic healthcare records-based surveillance system. PLoS One 2013;8:e72148	10.1371/journal.pone.0072148	Methodology / data quality	Analytical	Cohort	Pooled analysis: individual patient data	3: HSD (Italy); IPCI; Pedianet	4
104	Coloma PM, Trifirò G, Schuemie MJ, et al. Electronic healthcare databases for active drug safety surveillance: is there enough leverage?.Pharmacoepidemiol Drug Saf 2012;21:611–21	10.1002/pds.3197	Drug safety	Descriptive	Cohort	Pooled analysis: semi-aggregate data	4: HSD (Italy); IPCI; Pedianet; QResearch	4
105	de Bie S, Coloma PM, Ferrajolo C, et al. The role of electronic healthcare record databases in paediatric drug safety surveillance: a retrospective Cohort. Br J Clin Pharmacol 2015;80:304–14	10.1111/bcp.12610	Drug safety	Descriptive	Cohort	Pooled analysis: semi-aggregate data	3: HSD (Italy); IPCI; Pedianet	4
106	de Groot MCH, Klungel OH, Leufkens HGM, et al. Sources of heterogeneity in case-control studies on associations between statins, ACE-inhibitors, and proton pump inhibitors and risk of pneumonia. Eur J Epidemiol 2014;29:767–75	10.1007/s10654-014-9941-0	Drug safety	Analytical	Case-control	Data not combined	3: AHC (Mondriaan); LRGP; NPCRD (Mondriaan)	2
107	de Groot MCH, Schuerch M, de Vries F, et al. Antiepileptic drug use in seven electronic health record databases in Europe: a methodologic comparison. Epilepsia 2014;55:666–73	10.1111/epi.12557	Drug utilization	Descriptive	Cohort	Data not combined	5: AHC (Mondriaan); BIFAP; CPRD; NPCRD (Mondriaan); THIN	2
108	Ferrajolo C, Coloma PM, Verhamme KMC, et al. Signal detection of potentially drug-induced acute liver injury in children using a multi-country healthcare database network. Drug Saf 2014;37:99–108	10.1007/s40264-013-0132-9	Drug safety	Analytical	Multiple: cohort; SCCS	Pooled analysis: individual patient data	3: HSD (Italy); IPCI; Pedianet	4
110	Huerta C, Abbing-Karahagopian V, Requena G, et al. Exposure to benzodiazepines (anxiolytics, hypnotics and related drugs) in seven European electronic healthcare databases: a cross-national descriptive study from the PROTECT-EU Project. Pharmacoepidemiol Drug Saf 2016;25 Suppl 1:56–65	10.1002/pds.3825	Drug utilization	Descriptive	Cohort	Data not combined	5: AHC (Mondriaan); BIFAP; CPRD; NPCRD (Mondriaan); THIN	2
111	Korhonen P, Heintjes EM, Williams R, et al. Pioglitazone use and risk of bladder cancer in patients with type 2 diabetes: retrospective Cohort using datasets from four European countries. BMJ 2016;354:i3903	10.1136/bmj.i3903	Drug safety	Analytical	Cohort	Multiple: pooled IPD + meta-analysis (RE + FE)	2: CPRD; PHARMO GP	3
112	LoCasale R, Kern DM, Chevalier P, et al. Description of cardiovascular event rates in patients initiating chronic opioid therapy for noncancer pain in observational cohort studies in the US, UK, and Germany. Adv Ther 2014;31:708–23	10.1007/s12325-014-0131-y	Drug safety	Descriptive	Cohort	Data not combined	2: CPRD; Disease Analyzer (Germany)	1
113	Masclee GMC, Valkhoff VE, Coloma PM, et al. Risk of upper gastrointestinal bleeding from different drug combinations. Gastroenterology 2014;147:784-792.e9	10.1053/j.gastro.2014.06.7	Drug safety	Analytical	SCCS	Multiple: pooled IPD + meta-analysis (RE)	3: HSD (Italy); IPCI; Pedianet	4
114	Mokhles MM, Trifirò G, Dieleman JP, et al. The risk of new onset heart failure associated with dopamine agonist use in Parkinson's disease. Pharmacol Res 2012;65:358–64	10.1016/j.phrs.2011.11.009	Drug safety	Analytical	Case-control	Multiple: pooled IPD + meta-analysis (RE + FE)	3: HSD (Italy); IPCI; THIN	1
115	Rottenkolber M, Voogd E, van Dijk L, et al. Seasonal changes in prescribing of long-acting beta-2-agonists-containing drugs. Respir Med 2015;109:828–37	10.1016/j.rmed.2015.01.010	Drug utilization	Descriptive	Cohort	Data not combined	5: AHC (Mondriaan); BIFAP; CPRD; NPCRD	1

For peer review only

Study ID[a]	Citation	Resource locator: [doi unless specified otherwise][b]	Study topic	Study type	Study design[c]	Data combining	Primary care EHR data sources[d]	Other sources (if applicable)
							(Mondriaan); THIN	
116	Rottenkolber M, Voogd E, van Dijk L, et al. Time trends of period prevalence rates of patients with inhaled long-acting beta-2-agonists-containing prescriptions: a European comparative database study. PLoS One 2015;10:e0117628	10.1371/journal.pone.0117628	Drug utilization	Descriptive	Cohort	Data not combined	5: AHC (Mondriaan); BIFAP; CPRD; NPCRD (Mondriaan); THIN	2
117	Trifirò G, de Ridder M, Sultana J, et al. Use of azithromycin and risk of ventricular arrhythmia. CMAJ 2017;189:E560–8	10.1503/cmaj.160355	Drug safety	Analytical	Case-control	Multiple: pooled IPD + meta-analysis (RE)	3: HSD (Italy); IPCI; THIN	4
118	Tyczynski JE, Oleske DM, Klingman D, et al. Safety assessment of an anti-obesity drug (sibutramine): a retrospective Cohort. Drug Saf 2012;35:629–44	10.2165/11599220-000000000-00000	Drug safety	Analytical	Cohort	Data not combined	2: Disease Analyzer (Germany); Disease Analyzer (UK)	
119	Coloma PM, Schuemie MJ, Trifirò G, et al. Combining electronic healthcare databases in Europe to allow for large-scale drug safety monitoring: the EU-ADR Project. Pharmacoepidemiol Drug Saf 2011;20:1–11	10.1002/pds.2053	Drug safety	Analytical	Cohort	Data not combined	4: HSD (Italy); IPCI; Pedianet; QResearch	4
121	Sen EF, Verhamme KMC, Neubert A, et al. Assessment of pediatric asthma drug use in three European countries; a TEDDY study. Eur J Pediatr 2011;170:81–92	10.1007/s00431-010-1275-7	Drug utilization	Descriptive	Cohort	Data not combined	3: Disease Analyzer (UK); IPCI; Pedianet	
122	Cainzos-Achirica M, Varas-Lorenzo C, Pottegård A, et al. Methodological challenges when evaluating potential off-label prescribing of drugs using electronic health care databases: A case study of dabigatran etexilate in Europe. Pharmacoepidemiol Drug Saf 2018;27:713–23	10.1002/pds.4416	Drug utilization	Descriptive	Cross-sectional	Data not combined	2: CPRD; LPD (France)	1
124	Martín-Merino E, Petersen I, Hawley S, et al. Risk of venous thromboembolism among users of different anti-osteoporosis drugs: a population-based cohort analysis including over 200,000 participants from Spain and the UK. Osteoporos Int 2018;29:467–78	10.1007/s00198-017-4308-5	Drug safety	Analytical	Cohort	Data not combined	2: BIFAP; CPRD	
125	Anyanwagu U, Owen K, Mamza J, et al. Demographics, insulin use and clinical targets in type 2 diabetes insulin users: comparison of a local integrated diabetes service vs a UK-wide cohort. Pract Diabetes 2017;34:123–8	10.1002/pdi.2099	Health services research	Descriptive	Cross-sectional	Data not combined	2: EIDS; THIN	
127	Chiquette E, Oral EA, Garg A, et al. Estimating the prevalence of generalized and partial lipodystrophy: findings and challenges. Diabetes Metab Syndr Obes 2017;10:375–83	10.2147/DMSO.S130810	Disease epidemiology	Descriptive	Cross-sectional	Data not combined	2: CPRD; Humedica	2
128	Lo Re V, Carbonari DM, Saine ME, et al. Postauthorization safety study of the DPP-4 inhibitor saxagliptin: a large-scale multinational family of cohort studies of five outcomes. BMJ open diabetes Res care 2017;5:e000400	10.1136/bmjdrc-2017-000400	Drug safety	Analytical	Cohort	Meta-analysis: method not specified	2: CPRD; THIN	2
129	Price D, Thomas V, von Ziegenweidt J, et al. Switching patients from other inhaled corticosteroid devices to the Easyhaler(®): historical, matched-Cohort of real-life asthma patients. J Asthma Allergy 2014;7:31–51	10.2147/JAA.S59386	Drug comparative effectiveness	Analytical	Cohort	Pooled analysis: individual patient data	2: CPRD; OPCRD	
134	Coloma PM, Valkhoff VE, Mazzaglia G, et al. Identification of acute myocardial infarction from electronic healthcare records using different disease coding systems: a validation study in three European countries. BMJ Open 2013;3:e002862	10.1136/bmjopen-2013-002862	Drug safety	Analytical	Cohort	Pooled analysis: individual patient data	2: HSD (Italy); IPCI	1
135	Mor A, Frøslev T, Thomsen RW, et al. Antibiotic use varies substantially among adults: a cross-national study from five European Countries in the ARITMO project. Infection 2015;43:453–72	10.1007/s15010-015-0768-8	Drug utilization	Descriptive	Cohort	Data not combined	2: HSD (Italy); THIN	4
136	Poluzzi E, Diemberger I, De Ridder M, et al. Use of antihistamines and risk of ventricular tachyarrhythmia: a nested Case-control in five European countries from the ARITMO project. Eur J Clin Pharmacol 2017;73:1499–510	10.1007/s00228-017-2317-0	Drug safety	Analytical	Case-control	Multiple: pooled IPD + meta-analysis (RE)	3: HSD (Italy); IPCI; THIN	4
137	Roberto G, Leal I, Sattar N, et al. Identifying Cases of Type 2 Diabetes in Heterogeneous Data Sources: Strategy from the EMIF Project. PLoS One 2016;11:e0160648	10.1371/journal.pone.0160648	Methodology / data quality	Other	Cross-sectional	Data not combined	3: HSD (Italy); IPCI; THIN	5

Study ID[a]	Citation	Resource locator: [doi unless specified otherwise][b]	Study topic	Study type	Study design[c]	Data combining	Primary care EHR data sources[d]	Other sources (if applicable)
138	La Gamba F, Corrao G, Romio S, et al. Combining evidence from multiple electronic health care databases: performances of one-stage and two-stage meta-analysis in matched case-control studies. Pharmacoepidemiol Drug Saf 2017;26:1213–9	10.1002/pds.4280	Drug safety	Analytical	Case-control	Multiple: pooled IPD + meta-analysis (FE)	2: IPCI; THIN	1

[a] Record identifier used in reference management, and different from reference number used in main study text.
[b] If digital object identifier [doi] was not available, Pubmed ID [pmid] was used
[c] SCCS – self controlled case series; CCX – case crossover:
[d] See Table S2 for further details of primary care EHR data sources

For peer review only

Table S2: Summary details of primary care electronic health record (EHR) data sources used in studies included in systematic review.

Data Source ID	Short name	Source Name	Country	Clinical Coding	Drug Coding	Source reference	Resource locator[a]
1	CPRD	Clinical Practice Research Datalink	UK	Read V2	DM+D	https://www.ncbi.nlm.nih.gov/pubmed/26050254	doi: 10.1093/ije/dyv098
2	THIN	The Health Information Network	UK	Read V2	DM+D	https://www.ncbi.nlm.nih.gov/pubmed/22828580	doi: 10.14236/jhi.v19i4.820
3	QResearch	QResearch	UK	Read V2 / SNOMED-CT	DM+D	https://bmjopen.bmj.com/content/5/9/e008503.long	doi:10.1136/bmjopen-2015-008503
4	IPCI	Integrated Primary Care Information database	Netherlands	ICPC	ATC	https://www.ncbi.nlm.nih.gov/pubmed/10805025	doi:10.1055/s-0038-1634402
5	Pedianet	PediaNet	Italy		ATC	https://www.ncbi.nlm.nih.gov/pubmed/15930187	doi:10.1542/peds.2004-0040
7	SAIL	SAIL Databank	UK	Mixed	DM+D	http://www.ncbi.nlm.nih.gov/pubmed/19732426	doi:10.1186/1472-6963-9-157
8	LPD (France)	Cegedim LPD (Longitudinal Patient Data): France	France	ICD-9/10 CM	ATC	https://epidemiologie-france.aviesan.fr/en/ccontent/pdf/(ObjectId)/91221	url:https://epidemiologie-france.aviesan.fr/en/ccontent/pdf/(ObjectId)/91221
9	NPCRD (Mondriaan)	Netherlands Primary Care Research Database (NPCRD)	Netherlands	ICPC/ICD	ATC	http://www.ncbi.nlm.nih.gov/pubmed/25154551	doi:10.1016/s1098-3015(10)74933-0
11	BIFAP	Spanish Base de datos para la Investigación Farmacoepidemiológica en Atención Primaria (BIFAP)	Spain	ICPC	ATC	https://www.ncbi.nlm.nih.gov/pubmed/25066450	doi:10.1007/s00228-012-1386-3
12	HSD (Italy)	Health Search Database (Italy)	Italy	ICD-9 CM	ATC	https://www.ncbi.nlm.nih.gov/pubmed/24816637	doi:10.1371/journal.pone.0095419
15	AHC (Mondriaan)	Mondriaan Almere Healthcare group (AHC) database	Netherlands	ICPC/ICD	ATC	https://www.valueinhealthjournal.com/article/S1098-3015(10)74933-0/abstract	doi:10.1016/s1098-3015(10)74933-0
17	SIDIAP	Sistema de Información para el desarrollo de la Investigación en Atención Primaria	Spain	ICD-10	ATC	http://www.sidiap.org	url:http://www.sidiap.org
25	CDARS	Hong Kong Clinical Data Analysis and Reporting System database	Hong Kong	ICD-9 CM		https://www.ncbi.nlm.nih.gov/pubmed/24833754	doi:10.1093/jac/dku145
26	ResearchOne	ResearchOne	UK	CTV3	DM+D	https://www.ncbi.nlm.nih.gov/pubmed/26944937	url:http://www.researchone.org/
28	Julius (Mondriaan)	Julius Primary Care Network Database	Netherlands		ATC	https://www.ncbi.nlm.nih.gov/pubmed/30253760	doi:10.1016/s1098-3015(10)74933-0
29	NWEH-LDB	NorthWest EHealth linked database	UK	Read V2/SNOMED-CT	DM+D	https://bmcmedinformdecismak.biomedcentral.com/articles/10.1186/s12911-015-0132-z	doi:10.1186/s12911-015-0132-z
30	OCHIN Clarity (Epic EHR)	Oregon Community Health Information Network (OCHIN)	USA	ICD-9 CM	ATC	https://www.ncbi.nlm.nih.gov/pubmed/26934626	doi:10.7812/TPP/15-081
31	KP Clarity (Epic EHR)	Kaiser Permanente Epic EHR Clarity database	USA	ICD-9 CM	ATC	https://www.ncbi.nlm.nih.gov/pubmed/26934626	doi:10.7812/TPP/15-081
32	OPCRD	Optimum Patient Care Research Database (OPCRD)	UK	Read V2	DM+D	https://optimumpatientcare.org/database-overview/	url:https://optimumpatientcare.org/database-overview/
33	LPD (Spain)	IMS Longitudinal Patient Database (Spain)	Spain	ICD-9/10 CM	ATC	https://www.ncbi.nlm.nih.gov/pubmed/27155295	doi:10.1111/ejh.12776
34	LPD (Germany)	IMS Longitudinal Patient Database (Germany)	Germany	ICD-9/10 CM	ATC	https://www.ncbi.nlm.nih.gov/pubmed/27155295	doi:10.1111/ejh.12776
35	LPD (Belgium)	IMS Longitudinal Patient Database (Belgium)	Belgium	ICD-9/10 CM	ATC	https://www.ncbi.nlm.nih.gov/pubmed/27155295	doi:10.1111/ejh.12776
39	SIR	Salford Integrated Record (SIR)	UK	Read V2	DM+D	https://www.ncbi.nlm.nih.gov/pubmed/25481707	doi:10.1007/s00125-014-3456-9
41	Disease Analyzer (Germany)	IMS Disease Analyzer (Germany)	Germany	ICD-10	ATC	https://www.ncbi.nlm.nih.gov/pubmed/19825325	doi:10.5414/cpp47617
42	Disease Analyzer (France)	IMS Disease Analyzer (France)	France	ICD-10	ATC	https://www.ncbi.nlm.nih.gov/pubmed/19825325	doi:10.5414/cpp47617

For peer review only

Data Source ID	Short name	Source Name	Country	Clinical Coding	Drug Coding	Source reference	Resource locator[a]
46	Disease Analyzer (UK)	IMS Disease Analyzer (UK)	UK	ICD-10	ATC	https://bpspubs.onlinelibrary.wiley.com/doi/full/10.1111/j.1365-2125.2005.02450.x	doi:10.1111/j.1365-2125.2005.02450.x
51	DIN	Doctors' Independent Network (DIN)	UK	Read V2	BNF	https://www.ncbi.nlm.nih.gov/pubmed/15171985	doi:10.1016/j.ijmedinf.2004.02.002
52	Intego	Intego Network	Belgium	ICPC	ATC	http://www.ncbi.nlm.nih.gov/pubmed/24906941	doi:10.1186/1472-6947-14-48
53	Jonkoping County	Jonkoping County Primary Care Database	Sweden	ICD-10	ATC	https://www.ncbi.nlm.nih.gov/pubmed/28277045	doi:10.1080/02813432.2017.1288680
58	Resoprim	Resoprim Project	Belgium	ICPC	ATC	https://www.ncbi.nlm.nih.gov/pubmed/18953130	doi:10.3233/978-1-58603-922-6-103
59	Mediplus	IMS Mediplus	UK	Read V2	BNF	https://www.ncbi.nlm.nih.gov/pubmed/16368704	doi:10.1093/fampra/cmi106
62	CiPCA	Consultations in Primary Care Archive	UK	Read V2	BNF	https://www.keele.ac.uk/mrr/cipcadatabase/	url:https://www.keele.ac.uk/mrr/cipcadatabase/
65	THALES	THALES Database	France	ICD-9 CM	ATC	http://www.ncbi.nlm.nih.gov/pubmed/11111209	doi:10.1159/000052402
72	LRGP	Leidsche Rijn GP database (Mondriaan)	Netherlands	ICPC	ATC	https://www.ncbi.nlm.nih.gov/pubmed/15921047	doi:10.1007/s10654-004-5689-2
74	PHARMO GP	PHARMO GP database	Netherlands	ICPC	ATC	https://www.ncbi.nlm.nih.gov/pubmed/27530399	doi:10.1136/bmj.i3903
76	EIDS	Erewash (Integrated) Diabetes Service	UK	Read V2	DM+D	https://onlinelibrary.wiley.com/doi/full/10.1002/pdi.2099	doi:10.1002/pdi.2099
79	Humedica	Humedica NorthStar from Optum	USA	ICD-9 CM	ATC	https://www.nihcollaboratory.org/Pages/OptumInsight-MetaData-Table.aspx	doi:10.2147/DMSO.S130810
80	GE Healthcare	GE Healthcare Database	USA	ICD-9 CM	ATC	https://www.ncbi.nlm.nih.gov/pubmed/29066925	doi:10.2147/DMSO.S130810

[a] doi: Digital object identifier; url: Uniform resource locator

For peer review only

Multidatabase Systematic Review: Supplementary Appendix

Appendix S1: Review Protocol: Approaches for combining primary care EHR data from multiple sources: a systematic review of observational studies

For peer review only

Appendix S1: Review Protocol: Approaches for combining primary care EHR data from multiple sources: a systematic review of observational studies

Contents

1 Introduction 2
2 Rationale and scope 2
2.1 Objectives 2
3 Methods 2
3.1 Eligibility Criteria 2
3.2 Information sources 3
3.3 Search strategy 3
3.3.1 Test sets 3
3.3.2 Conceptual searches 3
3.3.3 Search of named databases and common terms 4
3.4 Study records 4
3.4.1 Data Management 4
3.4.2 Selection process 4
3.4.3 Data extraction 5
4 Data synthesis 6
5 References 7
6 Appendices 8
6.1 Appendix I: OVID Medline Search Strategy for Systematic Review 8
6.2 Appendix 2: Data extraction tables 10

For peer review only

For peer review only - http://bmjopen.bmj.com/site/about/guidelines.xhtml

1 Introduction

A recent systematic review evaluated methods for data management and analysis of multi-database studies in pharmacoepidemiology. The current study adopted a similar approach, but with a specific focus on studies combining primary care EHR databases, while expanding the scope to include all areas of observational epidemiology and healthcare database research.

2 Rationale and scope

The primary aim was to describe the full range of completed studies which brought together primary care electronic health record (EHR) data from two or more sources, and to generate a clear overview of methods used to manage, assess variability in, and analyse the data. The main motivation for the review was to inform a planned study of cancer risk in patients with Huntington's disease combining data from two UK primary care EHR databases.

The review specifically covered 'horizontal' combination of data from different sources, containing data from different sets of individuals (possibly after deduplication). Here the primary purpose of combining might be to increase the number of individuals available for analysis, or increase the range of population settings to which findings can be applied (i.e. increase external validity). The review did not include studies where data sources were only combined 'vertically' i.e. linkage studies whereby data on the same individuals was combined to provide richer (deeper) information about each study participant.

As the focus was on analysis of primary care EHR data, the review was restricted to studies using primary care EHR data from at least two sources. For the purpose of this review, primary care EHR data was defined as data collected by primary care clinicians and related staff for the purpose of diagnosis, treatment, management, and delivery of care of individual patients. It may include information collected or contributed by other care providers (1). It excludes data generated primarily for administrative purposes such as claims data.

The review was conducted following the PRISMA guidelines for reporting in systematic reviews (2,3) [http://www.prisma-statement.org].

2.1 Objectives

1. Identify studies which combined data from two or more sources of primary care EHR data.
2. Summarise key study characteristics, including the main reasons or motivations for combining data from different EHR databases
3. Describe the methods used to manage and analyse data including, where applicable, methods for combining data.
4. Describe the methods used to assess and report heterogeneity between primary care EHR data sources.
5. Describe and summarise any reported differences between different primary care EHR data sources.

Quality and completeness of reporting of methods was assessed using criteria adapted from the STROBE (4) and RECORD (5) guidelines. No formal assessment of quality in terms of risk of bias was attempted, either for individual studies, or for particular methodological approaches.

3 Methods

3.1 Eligibility Criteria

1. Peer reviewed, English language publication of an observational study.

For peer review only - http://bmjopen.bmj.com/site/about/guidelines.xhtml

2. Study participants selected from at least *two* different primary care EHR data sources.
3. Re-analyses of previously reported cohorts were included if they used substantially different methods.

There were no specific eligibility criteria relating to exposures, comparator groups, outcomes, or study design.

3.2 Information sources

The following databases were searched for eligible studies

1. Medline (OVID)
2. EMBASE (OVID)

3.3 Search strategy

The key challenges anticipated when searching for relevant studies were the lack of a specific MeSH concept for multi-database studies (6), and the lack of consensus on terminology for such studies in the published literature. This raised the possibility of having to hand search all database studies.

3.3.1 Test sets

Given the challenges outlined above, the performance of different search strategies was evaluated for their ability to recall results from the following test references sets:

> Test Set 1: 1673 publications using CPRD or GPRD databases were identified using keyword searches in Medline.
>
> Test Set 2: an *ad hoc* sample of 14 records identified from a published systematic review of multi-database pharmacoepidemiology studies (6), plus a small number identified from non-systematic review of the literature. All of these studies used at least one primary care EHR data source.

3.3.2 Conceptual searches

An initial search strategy was defined based on 3 concepts, identified using both MeSH terms and keywords, and the sensitivity of each concept was assessed against Test Sets 1 and 2 :

1. Database studies: this included MeSH terms and keyword searches for databases and related concepts such as Electronic Health Records, and Computerized Medical Records System. This concept was reasonably sensitive for recalling CPRD/GPRD studies (Test Set 1 sensitivity= 1365/1673 = 0.82), and all records in the sample of multi-database studies (Test Set 2 sensitivity 14/14 = 1). However it returned over 395 thousand results.
2. Primary care setting: a combined MeSH term and keyword search returned over 280 thousand records, but had very low sensitivity with both Test Set 1 (526/1673 = 0.31), and only moderate sensitivity with Test Set 2 (10/14 = 0.71).
3. (Observational) epidemiology studies: The InterTASC Information Specialists' Sub-Group Search Filter Resource (7) was accessed to identify potentially suitable and validated search filters. Waffenschmidt *et al* (8) reviewed search strategies to identify epidemiological studies, concluding that there was "no suitable approach to conducting *efficient* systematic searches for epidemiologic publications in bibliographic databases". One filter, from a systematic review of Hepatitis C prevalence in prisons by Larney *et al* (9), was found to be suitably sensitive, recalling almost 96% of their test set of 729 references. This filter had very high sensitivity for CPRD/GPRD studies in Test Set 1 (1593/1673 = 0.95), and also recalled all records in Test Set 2 (sensitivity 14/14 = 1). However it returned over 6 million records.

For peer review only - http://bmjopen.bmj.com/site/about/guidelines.xhtml

A Medline search combining all 3 of the concepts above returned 14309 records and recalled only 10 of the 14 records in Test Set 2 (sensitivity 0.71), with the sensitivity being limited by the 'Primary Care' concept. However any attempt to broaden this concept to improve sensitivity would have increased the number of recalled records beyond what could be feasibly reviewed manually. For example, broadening the Primary Care concept to include 'population-based' studies, allowed recall of 12/14 records from Test Set 2, but increased the total number of records retrieved to 23656.

3.3.3 Search of named databases and common terms

Given the relatively poor performance of the conceptual searches, an alternative strategy was developed using a combination of named databases, and commonly used key words and phrases. Three sources were used to compile a list of candidate primary care EHR (or closely related) databases:

1. ENCePP Resources Database: the European Network of Centres for Pharmacoepidemiology and Pharmacovigilance (ENCePP) maintains a searchable database of research organisations, networks and data sources (10). Despite a strong European focus, the register is not restricted to European data sources. The database was searched to identify 20 registered data sources identified as a 'Routine primary care electronic patient registry'.
2. B.R.I.D.G.E. TO DATA®: a "non-profit online reference describing population healthcare databases for use in epidemiology and health outcomes research" (10). A search interface is provided as a subscription service, however a simple listing of 286 named database resources was downloaded (23 Jan 2018) and searched for candidate primary care databases.
3. A list of 41 databases or primary care research networks identified in a 2017 review by Gentil et al (11) which examined factors associated with successful implementation of initiatives to collect and curate collections of primary care electronic health record data.

Abstracts of published studies identified from the initial named database search were scanned for additional terms and phrases used to describe the primary care EHR data sources, and these were added to the final search. Finally, reference lists of papers selected for full review were searched for additional studies.

The full search strategy used to search the Medline database is included in Appendix I.

This search was able to recall 12/14 records in Test Set 2 (sensitivity = 0.86), and all records in Test Set 1 (by definition - since CPRD/GPRD were among the named databases included in the search).

3.4 Study records

3.4.1 Data Management

All search results were exported from OVID in batches, with copies of export files retained. The references were imported into Mendeley V1.1 (Mendeley, Elsevier, Amsterdam, NL). Details of studies selected for full review were exported into a Microsoft Access database, in which was used to record subsequent inclusion/exclusion decisions and data extraction.

3.4.2 Selection process

Initial screening of all selected titles and abstracts was undertaken by 1 reviewer (DD). A second reviewer (MC) screened a 20% random sample of all abstracts. Full text was reviewed in instances where it was not possible to assess eligibility from the title and abstract alone.

Full text was then obtained for all papers selected during the initial abstract screening, and read by two reviewers, who completed the eligibility assessment before and performing data extraction.

For peer review only - http://bmjopen.bmj.com/site/about/guidelines.xhtml

3.4.3 Data extraction

Each reviewer extracted standardised information via a data collection form into a review database (MS Access). The following information was collected:

Year of publication
Primary care EHR data source details:<br>x number of sources<br>x name(s) of database<br>x country
Other (non-primary care EHR) data source details:<br>x number of sources<br>x name(s) of database<br>x type of database e.g. claims, disease registry<br>x country
Study type or broad objective e.g.:<br>x Descriptive e.g. drug utilisation, disease epidemiology<br>x Comparative or hypothesis testing e.g. comparative treatment effectiveness, drug safety, disease epidemiology<br>x Disease risk prediction<br>x Methodology / data quality assessment<br>x Health service research
Study design e.g.:<br>x Cross sectional<br>x Case-control<br>x Cohort<br>x Case-only designs<br>x Time series
Target population(s) for study:
Main exposure(s) if applicable e.g.:<br>x Drug treatment<br>x Disease risk factor<br>x Other
Main outcome(s) if applicable e.g.:<br>x All cause mortality<br>x Disease<br>x Treatment patterns<br>x Other
Main analysis methods, including confounder control e.g.:<br>x descriptive using summary statistics<br>x incidence or prevalence calculations<br>x multiple regression modelling
Motivation or rationale for using and/or combining data sources e.g.:<br>x increase study power<br>x assess consistency of findings in multiple settings<br>x international comparisons
Assssement of heterogeneity of exposures, outcomes and effect estimates e.g.:<br>x descriptive only (no formal comparisons)<br>x univariate comparisons<br>x formal tests for heterogeneity (Q-test, I-test)
Main approach for combining data sources e.g.:<br>x Data not combined: results presented separately for each source<br>x Meta-analysis of aggregate results from each data source

For peer review only

For peer review only - http://bmjopen.bmj.com/site/about/guidelines.xhtml

x Meta-analysis of semi-aggregated results from each data source<br>x Pooled analysis of individual patient data
Data management and analysis e.g.:<br>x Data managed and analysed separately by each database partner<br>x Use of common protocol<br>x Use of common data model (study specific, or externally defined e.g. OMOP CDM)<br>x Use of common analysis programs<br>x Data management and analysis arrangements (distributed, central, hybrid)<br>x Data sharing model (individual, semi-aggregate, aggregate)

4 Data synthesis

Results will be summarised in tables which will describe .

- basic characteristics of included studies: study design, statistical method
- rationale for combining databases
- methods used to assess heterogeneity
- methods use for combining or synthesising results

Further narrative descriptions will focus on specific subgroups. For example analytical studies which combined two or more databases from the same country.

For peer review only - http://bmjopen.bmj.com/site/about/guidelines.xhtml

5 References

1. Häyrinen K, Saranto K, Nykänen P. Definition, structure, content, use and impacts of electronic health records: a review of the research literature. Int J Med Inform [Internet]. 2008 May;77(5):291–304. Available from: http://www.ncbi.nlm.nih.gov/pubmed/17951106

2. Liberati A, Altman DG, Tetzlaff J, Mulrow C, Gøtzsche PC, Ioannidis JPA, et al. The PRISMA Statement for Reporting Systematic Reviews and Meta-Analyses of Studies That Evaluate Health Care Interventions: Explanation and Elaboration. PLoS Med [Internet]. 2009 Jul 21;6(7):e1000100. Available from: http://dx.plos.org/10.1371/journal.pmed.1000100

3. Moher D, Liberati A, Tetzlaff J, Altman DG, PRISMA Group. Preferred reporting items for systematic reviews and meta-analyses: the PRISMA statement. Ann Intern Med [Internet]. 2009 Aug 18;151(4):264–9, W64. Available from: http://www.ncbi.nlm.nih.gov/pubmed/19622511

4. von Elm E, Altman DG, Egger M, Pocock SJ, Gøtzsche PC, Vandenbroucke JP, et al. The Strengthening the Reporting of Observational Studies in Epidemiology (STROBE) statement: guidelines for reporting observational studies. J Clin Epidemiol [Internet]. 2008 Apr;61(4):344–9. Available from: http://www.ncbi.nlm.nih.gov/pubmed/18313558

5. Benchimol EI, Smeeth L, Guttmann A, Harron K, Moher D, Petersen I, et al. The REporting of studies Conducted using Observational Routinely-collected health Data (RECORD) statement. PLoS Med [Internet]. 2015 Oct;12(10):e1001885. Available from: http://www.ncbi.nlm.nih.gov/pubmed/26440803

6. Bazelier MT, Eriksson I, de Vries F, Schmidt MK, Raitanen J, Haukka J, et al. Data management and data analysis techniques in pharmacoepidemiological studies using a pre-planned multi-database approach: a systematic literature review. Pharmacoepidemiol Drug Saf [Internet]. 2015 Sep [cited 2017 Feb 15];24(9):897–905. Available from: http://doi.wiley.com/10.1002/pds.3828

7. Glanville J, Lefebvre C, Wright K. ISSG Search Filter Resource [Internet] [Internet]. York (UK): The InterTASC Information Specialists' Sub-Group; 2008. 2008 [cited 2018 Mar 12]. Available from: https://sites.google.com/a/york.ac.uk/issg-search-filters-resource/how-to-cite-this-site

8. Waffenschmidt S, Hermanns T, Gerber-Grote A, Mostardt S. No suitable precise or optimized epidemiologic search filters were available for bibliographic databases. J Clin Epidemiol [Internet]. 2017 Feb 1 [cited 2017 Oct 17];82:112–8. Available from: http://www.sciencedirect.com/science/article/pii/S0895435616303663

9. Larney S, Kopinski H, Beckwith CG, Zaller ND, Jarlais D Des, Hagan H, et al. Incidence and prevalence of hepatitis C in prisons and other closed settings: Results of a systematic review and meta-analysis. Hepatology [Internet]. 2013 Oct [cited 2017 Oct 17];58(4):1215–24. Available from: http://www.ncbi.nlm.nih.gov/pubmed/23504650

10. ENCePP. ENCePP Resource Database [Internet]. [cited 2018 Jan 18]. Available from: http://www.encepp.eu/encepp/resourcesDatabase.jsp

11. Gentil M-L, Cuggia M, Fiquet L, Hagenbourger C, Le Berre T, Banâtre A, et al. Factors influencing the development of primary care data collection projects from electronic health records: a systematic review of the literature. BMC Med Inform Decis Mak [Internet]. 2017 Dec 25 [cited 2017 Oct 24];17(1):139. Available from: http://bmcmedinformdecismak.biomedcentral.com/articles/10.1186/s12911-017-0538-x

For peer review only - http://bmjopen.bmj.com/site/about/guidelines.xhtml

6 Appendices

6.1 Appendix I: OVID Medline Search Strategy for Systematic Review

Run on 16 Feb 2018 on Medline to Feb Week 2 2018

Item	Term
1	Lifelink.mp.
2	Disease Analyzer.mp.
3	(OsMed not dysplasia).mp.
4	EpiChron.mp.
5	(Integrated Primary Care Information or IPCI or Interdisciplinary Processing of Clinical Information).mp.
6	PHARMO.mp.
7	(Primary Care Clinical Informatics Unit or PCCIU).mp.
8	(BIFAP or Database for Pharmacoepidemiolog* Research in Primary Care or Base de datos para la Investigacion Farmacoepidemiologica en Atencion Primaria).mp.
9	(SIDIAP or Information System for the Development of Research in Primary Care or (Sistema and Desenvolupament and Atencio Primaria)).mp.
10	((LINH and database) or Netherlands Information Network of General Practice or Landelijk Informatie Netwerk Huisatsenzorg).mp.
11	(NIVEL adj3 database).mp.
12	(CPRD or Clinical Practice Research Data*).mp.
13	(GPRD or General Practice Research Data*).mp.
14	(OPCRD or Optimum Patient Care Research Data*).mp.
15	((THIN adj4 database) or Health Information Network or Health Improvement Network).mp.
16	(QResearch or Q Research).mp.
17	(ResearchOne or (Research One adj4 database*)).mp.
18	(DIN LINK or (DIN adj4 database*) or Doctors Independent Network).mp.
19	((SAIL adj4 Data*) or Secure Anon* Information Link*).mp.
20	(Arianna data* or (Caserta and database)).mp.
21	Pedianet.mp.
22	(Health Search and (Database or Dataset)).mp.
23	Longitudinal Patient Database.mp.
24	(mediplus and database).mp.
25	(centricity and (database* or EMR or electronic medical record*)).mp.
26	OCHIN.mp.
27	PHINEX.mp.
28	Regenstrief Medical Record.mp.
29	(Clalit and database).mp.
30	(Electronic Medical Record Administrative data Linked Database or EMRALD).mp.
31	(Intego or (database* and (general practice or primary care) and Belgi*)).mp.
32	Julius General Practi*.mp.
33	((primary care or primary health care or general practi* or family practi* or ambulatory care) adj4 database*).mp.
34	population database*.mp
35	(healthcare adj2 database*).mp
36	health care database*.mp
37	(electronic health* adj2 database*).mp

For peer review only - http://bmjopen.bmj.com/site/about/guidelines.xhtml

38	(population health* adj2 database*).mp
39	((EHR or electronic health record*) adj2 database*).mp
40	Or/1-39
41	limit 40 to (abstracts and english language and yr="2000 -Current")

For peer review only

For peer review only - http://bmjopen.bmj.com/site/about/guidelines.xhtml

6.2 Appendix 2: Data extraction tables

Table name: StudyInfo1

Description: Basic publication details

Completed for: All studies selected at initial screening round

Name	Type	Size	Description
StudyID	Long Integer	4	UNIQID for study
Authors	Long Text	-	
Title	Long Text	-	
JName	Short Text	255	Name of journal
JVol	Short Text	255	Journal volume
JPage	Short Text	255	Journal pages
YearPub	Long Integer	4	Year of publication

Table name: DataSource1

Description: Key information about each primary care EHR database

Completed for: Each primary care EHR database, plus partial details collected for other databases described in the included studies

Name	Type	Size	Description
DataSourceID	Long Integer	4	UNIQID for datasource
SourceName	Short Text	255	Full or official name of database
Shortname	Short Text	255	Short name for database
Aliases	Short Text	255	Other names used in published papers
IsEHR	Short Text	10	Is it a primary care EHR database
SourceType	Integer	2	What type of database (primary care EHR or some other type)
SourceCountry	Short Text	255	Country of database
ClinicalCoding	Short Text	255	Name of clinical coding scheme (if known)
DrugCoding	Short Text	255	Name of drug coding scheme (if known)
SourceInfo1	Long Text	-	Other relevant information about data source
SourceReference	Long Text	-	Key reference for data source

Table name: Review1

Description: Summary of review process, including whether publication was selected for full review

Completed for: All studies selected at initial screening round

Name	Type	Size	Description
Review1ID	Long Integer	4	record identifier
StudyID	Long Integer	4	ID number of paper being reviewed
ReviewerID	Integer	2	Reviewer: DD or MC (or adjudicated)
IncExc	Integer	2	Inclusion / exclusion with reasons
IncExcComment 1	Long Text	-	Comment on decision to include or exclude
Review1Date	Date With Time	8	Date of completion of review

For peer review only - http://bmjopen.bmj.com/site/about/guidelines.xhtml

Table name: Review2

Description: Full details of study objectives, methods and relevant results

Completed for: All studies included after full paper review

Name	Type	Size	Description
Review2ID	Long Integer	4	record identifier
StudyID	Integer	2	Study ID (FK)
ReviewerID	Short Text	20	Reviewer: DD or MC (or Adjudicated)
Review2Status	Short Text	255	set to "in progress" once data entry is started; user specifies when completed
Review2Date	Date With Time	8	autoset when status is set to completed
Objective	Short Text	50	Short description of study objectives
OBjectiveText	Long Text	-	Further details of study objectives, including quoted text from publication if relevant
TargetPop	Short Text	255	Short description of the target population or patient group for the study
TargetPopText	Long Text	-	Further details of target population, including quoted text from publication if relevant
MainExposures	Short Text	255	Lookup: category for main exposure(s)
MainExposuresText	Long Text	-	Further details of main exposure(s), including quoted text from publication if relevant
MainOutcomes	Short Text	255	Lookup: category for main outcome(s)
MainOutcomesText	Long Text	-	Further details of main outcome(s), including quoted text from publication if relevant
StudyType	Short Text	255	Lookup: type of study e.g drug safety; disease epidemiology;
StudyTypeText	Long Text	-	Further details of study type, including quoted text from publication if relevant
StudyDesign	Short Text	255	Lookup: study design e.g. cohort, case control, etc
StudyDesignText	Long Text	-	Further details of study design, including quoted text from publication if relevant
MainRationale	Short Text	255	Lookup: main reason for combining data from multiple sources
MainRationaleText	Long Text	-	Further details of study rationale, including quoted text from publication if relevant
Stats1	Short Text	255	Lookup: main statistical method or model used
Stats1Text	Long Text	-	Further details of statistical method or model including quoted text from publication if relevant
ExposureTime	Short Text	255	Lookup: main method for modelling exposure
ConfounderControl	Short Text	255	Lookup: main method for confounder adjustment
HeterogeneityAssess	Short Text	255	Lookup: main method for assessing heterogeneity
CombineMethod	Short Text	255	Lookup: main method for combining results
CombineMethodText	Long Text	-	Further details of combination methods including quoted text from publication if relevant

For peer review only - http://bmjopen.bmj.com/site/about/guidelines.xhtml

CompareExposure	Short Text	255	Lookup: main method for comparing exposure variables in each data source
CompareOutcome	Short Text	255	Lookup: main method for comparing outcome variables in each data source
CompareOther	Short Text	255	Lookup: main method for comparing other variables in each data source
CompareText	Long Text	-	Further details of methods used to compare variables including quoted text from publication if relevant
DataManagement	Short Text	255	Lookup: how was data managed e.g. central vs multicentre etc
DataManageText	Long Text	-	Further details of data management approach including quoted text from publication if relevant
Programming	Short Text	255	Lookup: how was programming managed e.g. central vs multicentre etc
ProgrammingText	Long Text	-	Further details of programming approach including quoted text from publication if relevant

For peer review only

Reporting checklist for meta-analysis of observational studies.

Based on the MOOSE guidelines.

Instructions to authors

Complete this checklist by entering the page numbers from your manuscript where readers will find each of the items listed below.

Your article may not currently address all the items on the checklist. Please modify your text to include the missing information. If you are certain that an item does not apply, please write "n/a" and provide a short explanation.

Upload your completed checklist as an extra file when you submit to a journal.

In your methods section, say that you used the MOOSEreporting guidelines, and cite them as:

Stroup DF, Berlin JA, Morton SC, Olkin I, Williamson GD, Rennie D, Moher D, Becker BJ, Sipe TA, Thacker SB. Meta-analysis of observational studies in epidemiology: a proposal for reporting. Meta-analysis Of Observational Studies in Epidemiology (MOOSE) group. JAMA. 2000; 283(15):2008-2012.

		Reporting Item	Page Number
**Title**			
	#1	Identify the study as a meta-analysis of observational research	1
**Abstract**			

For peer review only

	#2	Provide a structured summary including, as applicable: background; objectives; data sources; study eligibility criteria, participants, and interventions; study appraisal and synthesis methods; results; limitations; conclusions and implications of key findings; systematic review registration number (From PRISMA checklist)	2
**Background**			
	#3a	Problem definition	4
	#3b	Hypothesis statement	n/a
	#3c	Description of study outcomes	4
	#3d	Type of exposure or intervention used	4
	#3e	Type of study designs used	4-5
	#3f	Study population	n/a
**Methods**			
Search strategy	#4a	Qualifications of searchers (eg, librarians and investigators)	23
Search strategy	#4b	Search strategy, including time period included in the synthesis and keywords	4
Search strategy	#4c	Effort to include all available studies, including contact with authors	5
Search strategy	#4d	Databases and registries searched	5

For peer review only

Search strategy	#4e	Search software used, name and version, including special features used (eg, explosion)	5
Search strategy	#4f	Use of hand searching (eg, reference lists of obtained articles)	5
Search strategy	#4g	List of citations located and those excluded, including justification	Figure 1
Search strategy	#4h	Method of addressing articles published in languages other than English	n/a
Search strategy	#4i	Method of handling abstracts and unpublished studies	n/a
Search strategy	#4j	Description of any contact with authors	5
	#5a	Description of relevance or appropriateness of studies gathered for assessing the hypothesis to be tested	n/a
	#5b	Rationale for the selection and coding of data (eg, sound clinical principles or convenience)	n/a
	#5c	Documentation of how data were classified and coded (eg, multiple raters, blinding, and interrater reliability)	5
	#5d	Assessment of confounding (eg, comparability of cases and controls in studies where appropriate)	n/a
	#5e	Assessment of study quality, including blinding of quality assessors; stratification or regression on possible predictors of study results	n/a

For peer review only

	#5f	Assessment of heterogeneity	n/a
	#5g	Description of statistical methods (eg, complete description of fixed or random effects models, justification of whether the chosen models account for predictors of study results, dose-response models, or cumulative meta-analysis) in sufficient detail to be replicated	n/a
	#5h	Provision of appropriate tables and graphics	n/a
**Results**			
	#6a	Graphic summarizing individual study estimates and overall estimate	Figure 2
	#6b	Table giving descriptive information for each study included	7-8,10
	#6c	Results of sensitivity testing (eg, subgroup analysis)	n/a
	#6d	Indication of statistical uncertainty of findings	n/a
**Discussion**			
	#7a	Quantitative assessment of bias (eg. publication bias)	n/a
	#7b	Justification for exclusion (eg, exclusion of non–English-language citations)	n/a
	#7c	Assessment of quality of included studies	5
**Conclusion**			
	#8a	Consideration of alternative explanations for observed results	n/a
	#8b	Generalization of the conclusions (ie, appropriate for the data presented and within the domain of the literature review)	14

For peer review only

#8c	Guidelines for future research	15
#8d	Disclosure of funding source	23

None Reproduced with permission from JAMA. 2000. 283(15):2008-2012. Copyright © 2000 American Medical Association. All rights reserved.This checklist can be completed online using https://www.goodreports.org/, a tool made by the EQUATOR Network in collaboration with Penelope.ai

For peer review only

BMJ Open

Approaches for combining primary care electronic health record data from multiple sources: a systematic review of observational studies

Journal:	*BMJ Open*
Manuscript ID	bmjopen-2020-037405.R1
Article Type:	Original research
Date Submitted by the Author:	15-Jun-2020

Complete List of Authors:	Dedman, Daniel; Medicines and Healthcare Products Regulatory Agency, Clinical Practice Research Datalink; London School of Hygiene and Tropical Medicine, Faculty of Epidemiology and Population Health<br>Cabecinha, Melissa; University College London, Research Department of Primary Care and Population Health<br>Williams, Rachael; Medicines and Healthcare Products Regulatory Agency, Clinical Practice Research Datalink<br>Evans, Stephen; London School of Hygiene and Tropical Medicine, Faculty of Epidemiology and Population Health<br>Bhaskaran, Krishnan; London School of Hygiene and Tropical Medicine, Faculty of Epidemiology and Population Health<br>Douglas, Ian; London School of Hygiene and Tropical Medicine, Faculty of Epidemiology and Population Health

**Primary Subject Heading**:	Epidemiology
Secondary Subject Heading:	Public health
Keywords:	EPIDEMIOLOGY, STATISTICS & RESEARCH METHODS, PUBLIC HEALTH

SCHOLARONE™ Manuscripts

I, the Submitting Author has the right to grant and does grant on behalf of all authors of the Work (as defined in the below author licence), an exclusive licence and/or a non-exclusive licence for contributions from authors who are: i) UK Crown employees; ii) where BMJ has agreed a CC-BY licence shall apply, and/or iii) in accordance with the terms applicable for US Federal Government officers or employees acting as part of their official duties; on a worldwide, perpetual, irrevocable, royalty-free basis to BMJ Publishing Group Ltd ("BMJ") its licensees and where the relevant Journal is co-owned by BMJ to the co-owners of the Journal, to publish the Work in this journal and any other BMJ products and to exploit all rights, as set out in our licence.

The Submitting Author accepts and understands that any supply made under these terms is made by BMJ to the Submitting Author unless you are acting as an employee on behalf of your employer or a postgraduate student of an affiliated institution which is paying any applicable article publishing charge ("APC") for Open Access articles. Where the Submitting Author wishes to make the Work available on an Open Access basis (and intends to pay the relevant APC), the terms of reuse of such Open Access shall be governed by a Creative Commons licence – details of these licences and which Creative Commons licence will apply to this Work are set out in our licence referred to above.

Other than as permitted in any relevant BMJ Author's Self Archiving Policies, I confirm this Work has not been accepted for publication elsewhere, is not being considered for publication elsewhere and does not duplicate material already published. I confirm all authors consent to publication of this Work and authorise the granting of this licence.

For peer review only

Title: Approaches for combining primary care electronic health record data from multiple sources: a systematic review of observational studies

Authors:

Daniel Dedman[1,2], Melissa Cabecinha[3], Rachael Williams[1], Stephen J W Evans[2], Krishnan Bhaskaran[2], Ian J Douglas[2]

Affiliations:

1. Clinical Practice Research Datalink, Medicines and Healthcare products Regulatory Agency, London, UK.

2. Faculty of Epidemiology and Population Health, London School of Hygiene and Tropical Medicine, London, UK

3. Research Department of Primary Care and Population Health, University College London, London, UK

Corresponding author: Daniel Dedman.

Address: Clinical Practice Research Datalink (CPRD), 10 South Colonnade, Canary Wharf, London E14 4PU, UK.

Telephone: +44 (0)203 080 6703

Email: daniel.dedman@mhra.gov.uk

ORCID iD: 0000-0002-3699-5391

For peer review only

Abstract

Objective

To identify observational studies which used data from more than one primary care electronic health record (EHR) database, and summarise key characteristics including: objective and rationale for using multiple data sources; methods used to manage, analyse, and (where applicable) combine data; and describe the approaches used to assess and report heterogeneity between data sources.

Design

Systematic review of published studies.

Data sources

Pubmed and Embase databases, searched using list of named primary care EHR databases; supplementary hand searches of reference list of studies retained after initial screening.

Study selection

Observational studies published between January 2000 – May 2018 which included at least 2 different primary care EHR databases.

Results

6054 studies were identified from database and hand searches, and 109 were included in the final review, the majority published between 2014-2018. Included studies used 38 different primary care EHR data sources.

Forty-seven studies (44%) were descriptive or methodological. Of 62 analytical studies, 22 (36%) presented separate results from each database, with no attempt to combine them; 29 (48%) combined individual patient data in a 1-stage meta-analysis; and 21 (34%) combined estimates from each database using 2-stage meta-analysis. Discussion and exploration of heterogeneity was inconsistent across studies.

Conclusions

Comparing patterns and trends in different populations, or in different primary care EHR databases from the same populations, are important and common objectives for multi-database studies.

When combining results from several databases using meta-analysis, provision of separate results from each database is helpful for interpretation. We found that these were often missing, particularly for studies using 1-stage approaches, which also often lacked details of any statistical adjustment for heterogeneity and/or clustering. For 2 stage meta-analysis a clear rationale should be provided for choice of fixed effect (FE), and/or random effects (RE) or other models.

For peer review only

Article summary

Strengths and limitations of this study

- Our systematic review identified the increasing number of published observational studies which specifically used primary care electronic health record (EHR) data from two or more sources.
- There were no restrictions on study design, exposures or outcomes.
- In the absence of relevant Medical Subject Heading (MeSH) terms, the search strategy relied on an extensive list of named primary care EHR databases to achieve as comprehensive coverage as possible.
- The selected publications were independently reviewed by 2 researchers.
- The findings of this review may not apply to multi-database studies which did not use primary care EHR data sources

Keywords

Systematic review

Multi-database studies

Electronic health records

Primary care

Word count

3717 words (not including title page, abstract, references, figures, tables or acknowledgments)

For peer review only

Introduction

Multi-database observational studies are increasingly common. They are conducted for two main reasons: to compare results across diverse populations and healthcare settings; or to combine the data to increase statistical power. Primary care electronic health record (EHR) databases are particularly valuable because they provide longitudinal data on individuals, often over many years, and typically contain richer information on a broader range of exposures, risk factors and health outcomes than administrative databases.[1–3] Although individual primary care EHR databases are often relatively small, covering a single region or other national population subset, their growing availability in recent years is likely to further increase the importance of non-interventional studies which combine these databases. Guidelines identifying best practice in this context have yet to be established but would be of clear benefit for researchers working with multiple databases. An important preliminary step is to describe current practice, but there is no comprehensive summary of studies which used two or more primary care EHR databases, and the methods for combining them.

One previous systematic review focussed on multi-database pharmacoepidemiology studies with a pre-planned approach to combine data to evaluate drug-outcome associations.[4] In that review studies were not limited according to the types of databases used, but descriptive studies and those which did not combine results from different databases were excluded. The authors found that for data management arrangements, analysis of heterogeneity, and methods for combining data reporting was often inadequate, making interpretation of study results more challenging. Since the focus of that review was pharmacoepidemiology studies and a wide range of database types, a broader view of combined primary care EHR data for any study purpose remains lacking.

The aim of this systematic review was to identify and describe the full range of completed studies which brought together primary care electronic health record (EHR) data from two or more sources. The specific objectives were: to summarise key study characteristics, including the main reasons or motivations for including data from different EHR databases; to describe the methods used to manage, analyse, and (where applicable) combine data; and describe the approaches used to assess and report heterogeneity between primary care EHR data sources.

Methods

The review considered all multi-database studies, published in English language between 2000-2018, and which included at least two different primary care EHR databases or data sources, irrespective of whether other types of database were also included Primary care EHR data was defined as data collected by primary care clinicians and related staff for the purpose of diagnosis,

For peer review only - http://bmjopen.bmj.com/site/about/guidelines.xhtml

treatment, management, and delivery of care of individual patients, and could include information contributed by other care providers.[5] It excluded data generated primarily for administrative purposes such as health insurance claims data, where the motivation for recording is different. , Primary care EHR databases were considered irrespective of whether they were “vertically” linked (i.e. linked at the individual patient level) to another data source such as a disease registry or dispensing database. Each “vertically” linked primary care EHR database was treated as a single data source. Apart from the specific focus on primary care EHR databases, no other restrictions were applied in terms of populations, geography, study period, exposure, outcome or study design.

A previous systematic review highlighted the challenge of identifying multi-database studies, for which no specific MeSH terms exist.[4] An alternative approach was therefore used, based on a comprehensive list of named primary care EHR databases compiled from two online registers[6,7] and one systematic review of primary care data collection projects.[8] For each named database a keyword search was generated and run on Medline, and the results combined. Abstracts of published studies identified in this search were scanned for additional terms and phrases which might be used to describe the primary care EHR data sources, and from these additional keyword searches were generated. The final search strategy [see Supplementary materials] was used to identify studies in Medline and Embase databases published between January 2000 and May 2018.

Titles and abstracts of all retrieved studies were screened for eligibility by one reviewer (DD). A random 20% sample was also screened by a second reviewer (MC) and showed very good agreement between the two. Reference lists of papers selected for full review were hand searched for additional studies.

Full text was obtained for all papers selected during the initial screening, and read by two reviewers (DD, MC), who independently completed the final eligibility assessment and data extraction. Each reviewer extracted standardised information from the study publication and online supplementary materials (where available), which was entered into the review database (MS Access) via electronic data collection form developed by one of the reviewers (DD), and pilot tested with 7 studies. Information extracted included data sources used, main objectives, study design, study populations, exposure, comparators and outcomes, data management arrangements, and statistical methods. All discordant results were reviewed, and the final designation agreed by both reviewers. No additional information was sought from investigators.

Studies were classified as analytical if they estimated an exposure-outcome association, or descriptive otherwise. We noted whether and how between-database heterogeneity was assessed, how this informed the decision to combine the data, and whether a 1-stage meta-analysis of pooled individual patient data or 2-stage meta-analysis of study specific effect estimates was used, as well as choice of fixed-effects (FE) vs random-effect (RE) models. A clear rationale for using multiple data sources was not always stated, but in some instances could be inferred. For

For peer review only

analytical studies where results were combined using 1- or 2-stage meta-analysis, unless stated otherwise the rationale was assumed to be an increase in the statistical precision of the exposure-outcome effect estimate. Three main models for data management and analysis were considered, based on previous reviews:[4,9–11] a fully centralised model for management and analysis of the raw data provided by each contributing database; a fully distributed model where all data management and analysis was undertaken locally, and only fully aggregated results were shared; and a partially distributed model with local extraction and data management to generate standardised patient level or partially aggregated datasets in standardised format, which were then shared for final centralised analysis. Partially (or semi-) aggregated data summarises information on more than one individual (thereby enhancing privacy protection) while still allowing the pooling of data across databases for further analysis, including 1-stage meta-analysis. Examples include total person time and event counts for groups of patients sharing the same characteristics."

We noted whether studies used a global common data model (CDM) such as OMOP[12,13] or Sentinel,[14,15] and whether they were part of a wider program or initiative for developing database networks and methods for combining results – such as IMI-PROTECT,[16,17] EU-ADR,[18] or ARITMO.[19,20]

The focus of the review was on describing the range of multi-database studies and methods for combining primary care EHR data, rather than evaluating evidence of effectiveness of specific interventions. Given this, and in the absence of consensus or validated reporting guidelines for multi-database studies, no formal assessment of risk of bias or study quality was attempted.

The study protocol, including the final Medline search strategy and details of data items extracted is provided in ***Supplementary Appendix I***.

Patient and public involvement

Patients or the public were not involved in the design, or conduct, or reporting, or dissemination plans of our research.

Results

The Medline and EMBASE searches returned 6049 results, and a further 5 were identified by hand searches. After initial screening of abstracts, 138 papers were selected for full text review, and 109 were included in the final review (***Figure 1***). Summary information on the included studies is provided in ***Supplementary Table S1***.

Included studies used data from 38 different primary care EHR databases. Most studies (98, 89%) used 2 or 3 primary care EHR databases, and 43 studies (39%) also included one or more non-primary care EHR database. All but 3 studies used exclusively European primary care data, and 35 (32%) used data from a single country. Details of the primary care EHR databases used in included studies are summarised in ***Supplementary Table S2***.

For peer review only

The annual number of published studies increased over time (***Supplementary Figure S1***), with fewer than 10 studies per year between 2003 – 2013, while a peak in 2016 (25 studies) included 10 studies published in a special supplement on the IMI-PROTECT research program [16,17].

General characteristics of included studies are shown in **Table 1**. More than half (62, or 57%) were classified as analytical. Most studies (76, 70%) examined safety, effectiveness or utilisation of specific drugs accounted for, while 21% (23 studies) were disease epidemiology or risk prediction studies with no specific focus on pharmacological therapies.

For peer review only

Table 1: General characteristics of included studies, including objective, rationale and study design

	Study Type				
	Analytical	Descriptive	Other(a)	**All**	%
**All Studies**	**62**	**45**	**2**	**109**	
Study Objective					
Drug safety	37	5		**42**	**38.5**
Drug utilization	3	24	1	**28**	**25.7**
Disease epidemiology	7	9		**16**	**14.7**
Disease risk prediction	5	2		**7**	**6.4**
Drug comparative effectiveness	6			**6**	**5.5**
Methodology / data quality	2	3	1	**6**	**5.5**
Health services research	2	2		**4**	**3.7**
Main rationale for using multiple data sources (stated or inferred)					
Describe trends and variation between countries or settings	2	28		**30**	**27.5**
Increase study power	24	2		**26**	**23.9**
Examine consistency of findings in different settings (using a standardised approach or common study protocol)	19	3		**22**	**20.2**
Compare availability/quality of data in each source	2	7	2	**11**	**10.1**
Validation of findings in a second data source	5	3		**8**	**7.3**
Not clearly stated	10	2		**12**	**11.0**
Databases per study: Primary care EHR only					
Mean	2.4	2.9	2.5	**2.6**	
Median [range]	2 [2-4]	3 [2-5]	2.5 [2-3]	**2 [2-5]**	
Databases per study: all types					
Mean	3.1	4.3	5	**3.7**	
Median [range]	2 [2-8]	4 [2-8]	5 [2-8]	**3 [2-8]**	
Database setting					
Single country	25	9	1	**35**	**31.8**
Multi-country	37	36	1	**75**	**68.2**
Study design(b)					
Cohort study	33	38	1	**72**	**66.1**
Case-control study	23	0	0	**23**	**21.1**
Cross-sectional	1	6	1	**8**	**7.3**
Self controlled designs	7	0	0	**7**	**6.4**
Other	0	1	1	**2**	**1.8**
Interrupted time series	1	0	0	**1**	**0.9**
Data management and analysis model (stated or inferred)					
Centralised management and analysis: raw data shared	32	11	1	**44**	**40.4**
Distributed management and analysis: aggregated results shared	11	12		**23**	**21.1**
Distributed management + centralised analysis: patient level or partially aggregated data shared	11	8	1	**20**	**18.3**
Not described	8	14		**22**	**20.2**
Study drug (ATC Chapter)					
Nervous system (N)	11	8		**19**	**17.4**
Respiratory system (R)	9	5		**14**	**12.8**
Musculo-skeletal system (M)	9	3		**12**	**11.0**
Multiple categories	7	4		**11**	**10.1**
Alimentary tract and metabolism (A)	6	3	1	**10**	**9.2**
Antiinfectives for systemic use (J)	4	4		**8**	**7.3**

For peer review only

Cardiovascular system (C)	2	2		**4**	**3.7**
Genito urinary system and sex hormones (G)	2	1		**3**	**2.8**
Blood and blood forming organs (B)		2		**2**	**1.8**
Dermatologicals (D)		1		**1**	**0.9**
N/A	12	12	1	25	**22.9**
Study condition (ICD-10 chapter)					
Diseases of the circulatory system (I00-I99)	15	4		**19**	**17.4**
Diseases of the respiratory system (J00-J99)	11	4		**15**	**13.8**
Diseases of the digestive system (K00-K95)	9	4		**13**	**11.9**
Multiple categories	3	7		**10**	**9.2**
Endocrine, nutritional and metabolic diseases (E00-E89)	4	3	2	**9**	**8.3**
Injury, poisoning and certain other consequences of external causes (S00-T88)	6	1		**7**	**6.4**
Neoplasms (C00-D49)	5	1		**6**	**5.5**
Diseases of the musculoskeletal system and connective tissue (M00-M99)	4	1		**5**	**4.6**
Diseases of the nervous system (G00-G99)	2	3		**5**	**4.6**
Pregnancy, childbirth and the puerperium (O00-O9A)	1	3		**4**	**3.7**
Certain infectious and parasitic diseases (A00-B99)		2		**2**	**1.8**
Diseases of the skin and subcutaneous tissue (L00-L99)		2		**2**	**1.8**
Diseases of the blood and blood-forming organs and certain disorders involving the immune mechanism (D50-D89)		1		**1**	**0.9**
Diseases of the genitourinary system (N00-N99)	1			**1**	**0.9**
Health status inc. morbidity and/or mortality	1			**1**	**0.9**
Mental, Behavioral and Neurodevelopmental disorders (F01-F99)		1		**1**	**0.9**
N/A		8		**8**	**7.3**

(a) Other study type category included: case definition validation by chart review [1 study]; prescribing data quality assessment [1 study].

(b) Six studies included multiple designs and were therefore included each relevant category: case-control and cohort [3 studies]; case-crossover (CCX) and self-controlled case-series (SCCS) [2 studies]; cohort study and self-controlled case-series (SCCS) [1 study].

For peer review only

Cohort studies were the most common study design (72 studies, 66%), and the majority of these were descriptive. Six studies included more than 1 study design.

The most common approach for data management and analysis was a fully centralised model (44 studies, 40%). A fully distributed model was used in 23 studies (21%), including 15 studies conducted as part of the IMI-PROTECT program[16,21]. A partially distributed approach was used in 20 studies (18%), including 9 studies from the EU-ADR program,[18] and 5 from the ARITMO project.[19,20]. No studies reported using a common data model.

Methodological aspects of the 62 analytical studies are summarised in **Table 2**. All 23 case-control studies employed individual matching, and used conditional logistic regression to estimate adjusted odds ratios, but for cohort studies a range of statistical approaches was used.

For peer review only

Table 2: Methodological aspects of analytical studies (N=62)

Characteristic	Study design(a)					
	Case-control studies	Cohort studies	Self-controlled studies	Other(b)	All	%
All studies	**23**	**34**	**7**	**2**	**62**	
Statistical Methods(c)						
Logistic regression	23	8	2	1	**34**	**54.8**
Poisson regression		6	6		**12**	**19.4**
Cox regression		18			**18**	**29.0**
Other(d)		9	1	1	**11**	**17.7**
Confounder control(c)						
Multiple regression or Mantel Haenszel	23	32		2	**55**	**88.7**
Matching	23	9		1	**29**	**46.8**
Case only / Self-controlled design			7		**7**	**11.3**
Propensity scores		3			**3**	**4.8**
Instrumental variables		2			**2**	**3.2**
None		1			**1**	**1.6**
Database comparisons/heterogeneity assessment (c)						
Participant characteristics presented for each database	17	24	4	2	**45**	**72.6**
Effect estimates presented for each database	18	19	5	2	**41**	**66.1**
Formal test of effect heterogeneity	10	4	3		**17**	**27.4**
- I2	*(6)*	*(3)*	*(3)*		*(12)*	*(19.4)*
- Cochran's Q	*(2)*	*(1)*			*(3)*	*(4.8)*
- (other or not specified)	*(3)*				*(3)*	*(4.8)*
No database comparisons (combined effect estimates only)	5	11	2		**17**	**27.4**
Method for combining data or results(c)						
Data not combined	3	16	3	2	**22**	**35.5**
Meta-analysis (2-stage):	15	4	3		**21**	**33.9**
- random effects (RE)	*(10)*	*(2)*	*(2)*		*(13)*	*(21.0)*
- fixed effects (FE)	*(7)*	*(3)*	*(2)*		*(13)*	*(21.0)*
- method not specified		*(1)*			*(1)*	*(1.6)*
Pooled analysis (1-stage):	12	15	3		**29**	**46.8**
Multiple: 1-stage and 2-stage	*(7)*	*(1)*	*(2)*		*(10)*	*(16.1)*

(a) Six studies contributed to multiple categories because they included multiple designs: case-control and cohort [3 studies]; case-crossover (CCX) and self-controlled case-series (SCCS) [2 studies]; cohort study and self-controlled case-series (SCCS) [1 study].

(b) 1 cross sectional, 1 interrupted time series

(c) A single study could be included in more than 1 category.

(d) Other statistical methods included: Negative binomial regression (2); Mantel-Haenszel (2); 2-stage IV models (2); 'data-mining methods' (2); generalised linear models (1); univariate tests (1).

For peer review only

In 22 analytical studies (35%) data were not combined, and all results were presented separately for each database – usually in order to describe and assess the consistency of findings in different populations or settings, using a common study protocol and analysis approach. In the remaining 5 studies, risk prediction models were developed in one primary care EHR database, and validated using a second primary care EHR database from the same country (UK).[22–26]

In 40 analytical studies (65%), including the majority (20/23) of case-control studies, and half (18/34) of cohort studies, some form of pooled- or meta-analysis was undertaken. A 1-stage meta-analysis of pooled individual patient data (IPD) or partially aggregated data was undertaken in 29 studies (47%). In 19 of these studies, no assessment or discussion of between-database heterogeneity was provided, and only 4 studies reported any form of analytical adjustment for the clustered nature of the pooled data - in each case by including database as a covariate in a multiple regression model, with 1 study also including interaction terms between database and covariates.[27] Two-stage meta-analysis of database-specific effect estimates was used in 21 studies (34%), of which 14 presented some discussion or formal assessment of heterogeneity. The choice of FE or RE models was not clearly justified in most studies, though in 4 cases model choice was based on formal tests of heterogeneity (results not shown).

In 41 analytical studies (66%), separate effect estimates were reported for each database. Between database heterogeneity was formally assessed (most commonly using the I^2 statistic) in 17 studies (27%) and was discussed but not formally assessed in a further 6 studies (10%). In 17 studies (27%) – all of which used a 1-stage approach - results were presented for the combined dataset only. Only two of these 17 additionally included a 2-stage meta-analysis and reported heterogeneity statistics.

Ten studies reported comparable results (in the main paper or in supplementary materials) using two or more analytic approaches, such as both 1-stage and 2-stage meta-analysis (9 studies), or 2-stage analysis with both FE and RE models (1 study), summarised in **Figure 2**. In most cases 1-stage and 2-stage meta-analysis gave very similar results. Confidence intervals from RE meta-analysis were in almost every case wider than the corresponding FE or 1-stage model estimates. In one study (review database id:117) point estimates from 1-stage and 2-stage analyses differed appreciably, and RE model confidence intervals were wider, partly because some databases with very small numbers of cases were excluded.[28] In one self-controlled case series (SCCS) study examining risk of upper gastrointestinal bleeding with different drugs alone and in combination, the point estimates from 1-stage model (ignoring clustering), and 2-stage RE effects models were very different (review database id:113a-d), and confidence intervals from the 2-stage models were much wider.[29]

For peer review only

Discussion

This systematic review identified 109 multi-database studies which used data from 2 or more primary care EHR data sources, the great majority of which were from European countries. Just under half were descriptive studies undertaken either to compare patterns and trends in different populations or settings, or to assess comparability of different EHR databases from similar populations. In these descriptive studies, and in a third of analytical studies, there was no attempt to combine results from the different databases. Where data was combined, a 1-stage meta-analysis of pooled individual patient data was used more often than 2-stage meta-analysis of database specific results. Reporting of statistical methods for 1-stage analyses in particular was sub-optimal: in all but 4 such studies, adjustment for clustering or effect heterogeneity was either not explored or not reported.

Whether and how to combine data is a key consideration in multi-database studies, and our results are consistent with a previous systematic review which found that 16 out of 22 multi-database pharmacoepidemiology studies used a 1-stage approach to combine the data.[4]

One-stage meta-analysis approaches have gained popularity over the past 2 decades as a technique for combining individual *participant* data from randomised controlled trials and other clinical studies that collect primary data, identified in systematic reviews.[30–32] One-stage meta-analysis has a number of advantages relevant to multi-database studies which combine individual *patient* data from secondary data sources.[33] Firstly, it ensures standardisation of the statistical analysis across all data sources. Secondly it provides maximum flexibility to explore dose response patterns, subgroup analyses, and effect modification, all of which may help to account for heterogeneity between data sources. Thirdly, a one-stage approach can incorporate information from smaller databases with sparse data, even where the database specific effect cannot be reliably estimated due to zero cell counts.[28,34,35] However, one-stage meta-analysis of individual patient data should properly account for the clustered nature of the data from contributing databases,[27,36–39] since not doing so may introduce bias, especially if there is between study heterogeneity in effect estimates. The results of this review suggest that clustering is largely ignored in multi-database studies using primary care EHR data, and this is consistent with findings from other reviews of one-stage meta-analysis in systematic reviews,[30,31] and in multi-database pharmacoepidemiology studies.[4,27] Barriers to the adoption of methods that properly account for clustering may include the perceived statistical complexity, lack of options in commonly available statistical software, or because they can be computationally intensive.[27,40]

A third of analytical studies in this review used a 2-stage approach to combine database specific effect estimates to produce a pooled estimate. This approach avoids the need to share potentially sensitive individual patient data and may therefore be the only available option in some instances. It can also take advantage of local expertise and knowledge of each database partner, including optimising the use of available covariate information to control for confounding. In addition, a 2-

For peer review only

stage meta-analysis is relatively straightforward to implement and interpret. It is also possible to conduct pre-planned subgroup analyses, or examine dose-response effects using a 2-stage approach, although sparse data may limit this, since databases with zero cell counts in one or both comparison groups will usually have to be excluded, unless some form of continuity correction is used, which can introduce bias.[41]

As for meta-analysis of randomised and prospective studies, a second key consideration for multi-database studies is how to assess and interpret results in the presence of heterogeneity. Limitations associated with using secondary data not collected for the specific study question impose an additional challenge in this context.[1,2] Several approaches for harmonising analyses of multi-database studies have been used. These include development and adoption of consistent and validated case definitions, use of common protocols and statistical analysis plans, and shared data management and analysis routines, all aiming to reduce external sources of variability in results from different databases.[10,11,16,17,19] Nevertheless, in studies incorporating data from different countries with different population characteristics and healthcare systems, these factors may contribute to real differences in the effect estimates. Even within a single country, different primary care EHR software systems are used, which may introduce heterogeneity in the extracted data.[42–47] With 1-stage analysis, both clustering and heterogeneity can be naturally explored in hierarchical models incorporating fixed and/or random effects, though as already noted the tendency has been to ignore this issue in multi-database studies. For 2-stage meta-analysis, we found that fixed effect (FE) and random effects (RE) models were used equally frequently and model choice, where discussed at all, was generally related to the presence or absence of heterogeneity. Relatively few studies justified the choice based on some formal measure or test for heterogeneity, a practice which has been criticised because such tests often have low power. This is a problem especially when the number of studies or databases is small,[48] and can result in a FE model being used even though heterogeneity is present and a RE model may have been more appropriate.[49] Simulation studies have shown that the I^2 statistic can also be unreliable, either under- or over-estimating heterogeneity in certain circumstances, particular when the number of studies or databases is small.[50,51]

Several countries (Italy, Netherlands, Spain, UK) now have two or more primary care EHR databases, and combining sources from the same country may reduce heterogeneity. In such cases, a fixed effect model may be appropriate, especially where supporting analyses demonstrate substantial similarities in patient characteristics. Of 8 single-country studies identified in this review and using 2-stage meta-analysis, all 8 used fixed effects models[52–59] - despite evidence of substantial heterogeneity in some cases, although two studies did also use random effects models for some analyses.[52,53]

When combining primary care EHR data from different countries or settings, a random effects model might seem most appropriate, since these incorporate uncertainty in effect size when

For peer review only

heterogeneity is present, yet reduce to a fixed effect model if there is no heterogeneity.[48] However, when the number of estimates being combined is very small (<5) and heterogeneity is present – a common scenario in multi-database studies – conventional random effect models may perform poorly. Simulation studies show that they can produce confidence intervals which are too narrow, thereby increasing type 1 error rates.[60–63] A number of alternatives to conventional random effects models have been proposed which partially address these limitations in some circumstances,[60,63–66] nevertheless several authors have urged caution when interpreting results from meta-analysis of very few, heterogenous studies.[60,66,67] For multi-database studies, it may therefore be helpful to present estimates for both fixed and random effects models – or other alternative models, but always along with the results from individual databases.

Despite the differences outlined above, where studies combined data using more than one method they produced similar estimates in most cases. However, in at least one study, 1- and 2-stage methods yielded large differences in both the point estimates and their precision. This may in part be related to the substantial heterogeneity in the database specific estimates (reported I^2 between 86-98% for the estimates shown), but incomplete reporting of statistical methods limits further interpretation of these results. When the same modelling assumptions are used, 1- and 2-stages approaches are expected to give very similar results if the number of studies combined is relatively large [37]. However, few studies have systematically compared performance of 1- and 2-stage approaches for multi-database studies.[27,68]

Limitations

The search strategy for this review included a list of named primary care EHR databases compiled from publicly available registers. This was to circumvent the poor sensitivity and specificity of conceptual searches based on MeSH terms, as reported in a previous review,[4] and confirmed in the current review. Our approach could have missed some eligible studies - if the abstract only mentioned primary care EHR data sources that were not in our list or did not mention the use of health databases or related terms at all. We would also have missed non-English language and abstract only publications. Nevertheless, we expect the number of missed studies to be small, and any such studies are unlikely to have differed systematically from the included studies in terms of key methodological aspects. A more recent inventory of EHR databases in Europe did not identify any additional primary care databases that were not included in our review.[69] A further limitation was that some subjective interpretation was occasionally necessary to classify aspects of certain studies - for example the rationale for combining, or the methods for managing and analysing data. The use of two reviewers helped to achieve some consistency across the included studies.

In conclusion, we found a growing body of literature reporting on studies using two or more sources of primary care EHR data. These addressed a range of research questions, and in many

For peer review only

cases the results were presented separately and not combined. When data was combined, a 1-stage meta-analysis was preferred. One-stage methods offer advantages in terms of analytical flexibility but are only possible where data management and governance arrangements allow for sharing of individual patient data. However, in many studies using 1-stage approaches, the clustered nature of data from multi-database studies was frequently ignored, with unknown impact for interpretation. Two-stage meta-analysis requires only sharing of aggregated results, but there are known limitations with current 2-stage methods when the number of studies is small, especially when some heterogeneity is expected. Irrespective of whether a 1- or 2-stage approach is used, combined results should be accompanied by results from each data source separately. This information, together with clear and complete reporting on methods used to standardise and analyse the data (including the rationale for these decisions), affords a more considered assessment of potential sources of heterogeneity and greatly aids interpretation of the overall results. These considerations are relevant more widely to multi-database studies irrespective of the types of EHR or administrative databases being used, particularly where the number of databases being combined is relatively small, as was generally the case in our sample. Further research is required to understand the impact of analysis methods and other design aspects on overall study quality, and the development of reporting guidelines for multi-database studies, or extension of the existing RECORD guidance,[70] might be an important first step. **Box 1** summarises key recommendations arising from our review.

For peer review only

Box 1: Recommendations

Recommendation	Rationale
Studies should report clearly on all aspects of study design and conduct which impact on harmonisation of analyses across data sources.	Allows assessment of the relative importance of heterogeneity induced by data management and analysis decisions *vs.* heterogeneity inherent in the data.
Participant characteristics and effect estimates (where applicable) should be reported for each data source.	Assessment of heterogeneity is essential for interpretation, but formal methods for quantifying heterogeneity are inefficient and possibly biased in multi-database settings.
Where 1-stage methods are used, studies should report whether and how analyses accounted for clustering and between database heterogeneity.	Interpretation requires understanding of extent to which heterogeneity might influence study results.
Where 2-stage meta-analysis is used, studies should provide a clear rationale for choice of fixed effect (FE), random effects (RE), or other model.	Interpretation requires understanding of extent to which heterogeneity might influence study results.
Sensitivity analyses should include alternative methods for combining data	Comparing the results of 1-stage vs 2-stage analyses, or fixed vs random effects models provides information about potential impact of modelling assumptions.
Further research is needed to compare performance of 1-stage and 2-stage approaches for multi-database studies	Relatively few studies have specifically addressed meta-analysis for multi-database studies.

Figure and table legends

Figure 1 [Mono Image]: Selection and inclusion of studies for systematic review

Table 1: General study characteristics, including objective, rationale and study design

Table 2: Methodological aspects of analytical studies (N=62)

Figure 2 [Mono Image]: Comparison of relative risk (RR) estimates reported in studies using 2 or more methods to combine data from multiple sources

For peer review only

References

1 Strom BL. Overview of Automated Databases in Pharmacoepidemiology. In: *Textbook of Pharmacoepidemiology*. Chichester, UK: : John Wiley & Sons Ltd 2013. 118–22. doi:10.1002/9781118344828.ch08

2 Hall GC, Sauer B, Bourke A, *et al.* Guidelines for good database selection and use in pharmacoepidemiology research. *Pharmacoepidemiol Drug Saf* 2012;**21**:1–10. doi:10.1002/pds.2229

3 Devoe JE, Gold R, McIntire P, *et al.* Electronic health records vs Medicaid claims: completeness of diabetes preventive care data in community health centers. *Ann Fam Med* 2011;**9**:351–8. doi:https://dx.doi.org/10.1370/afm.1279

4 Bazelier MT, Eriksson I, de Vries F, *et al.* Data management and data analysis techniques in pharmacoepidemiological studies using a pre-planned multi-database approach: a systematic literature review. *Pharmacoepidemiol Drug Saf* 2015;**24**:897–905. doi:10.1002/pds.3828

5 Häyrinen K, Saranto K, Nykänen P. Definition, structure, content, use and impacts of electronic health records: a review of the research literature. *Int J Med Inform* 2008;**77**:291–304. doi:10.1016/j.ijmedinf.2007.09.001

6 ENCePP. ENCePP Resource Database. http://www.encepp.eu/encepp/resourcesDatabase.jsp (accessed 18 Jan 2018).

7 B.R.I.D.G.E-TO-DATA. DGI, LLC. https://www.bridgetodata.org/ (accessed 23 Jan 2018).

8 Gentil M-L, Cuggia M, Fiquet L, *et al.* Factors influencing the development of primary care data collection projects from electronic health records: a systematic review of the literature. *BMC Med Inform Decis Mak* 2017;**17**:139. doi:10.1186/s12911-017-0538-x

9 Brown JS, Holmes JH, Shah K, *et al.* Distributed health data networks: a practical and preferred approach to multi-institutional evaluations of comparative effectiveness, safety, and quality of care. *Med Care* 2010;**48**:S45-51. doi:10.1097/MLR.0b013e3181d9919f

10 Gini R, Schuemie M, Brown J, *et al.* Data Extraction And Management In Networks Of Observational Health Care Databases For Scientific Research: A Comparison Among EU-ADR, OMOP, Mini-Sentinel And MATRICE Strategies. *eGEMs (Generating Evid Methods to Improv patient outcomes)* 2016;**4**:2. doi:10.13063/2327-9214.1189

11 Lai EC-C, Stang P, Yang Y-HK, *et al.* International Multi-database Pharmacoepidemiology: Potentials and Pitfalls. *Curr Epidemiol Reports* 2015;**2**:229–38. doi:10.1007/s40471-015-0059-z

12 Schuemie MJ, Gini R, Coloma PM, *et al.* Replication of the OMOP experiment in Europe:

For peer review only

evaluating methods for risk identification in electronic health record databases. *Drug Saf* 2013;**36 Suppl 1**:S159-69. doi:10.1007/s40264-013-0109-8

13 Overhage JM, Ryan PB, Reich CG, *et al.* Validation of a common data model for active safety surveillance research. *J Am Med Inform Assoc* 2012;**19**:54–60. doi:10.1136/amiajnl-2011-000376

14 Toh S, Reichman ME, Houstoun M, *et al.* Comparative risk for angioedema associated with the use of drugs that target the renin-angiotensin-aldosterone system. *Arch Intern Med* 2012;**172**:1582–9. doi:10.1001/2013.jamainternmed.34

15 Platt R, Carnahan RM, Brown JS, *et al.* The U.S. Food and Drug Administration's Mini-Sentinel program: status and direction. *Pharmacoepidemiol Drug Saf* 2012;**21 Suppl 1**:1–8. doi:10.1002/pds.2343

16 Klungel OH, Kurz X, de Groot MCH, *et al.* Multi-centre, multi-database studies with common protocols: lessons learnt from the IMI PROTECT project. *Pharmacoepidemiol Drug Saf* 2016;**25 Suppl 1**:156–65. doi:10.1002/pds.3968

17 Reynolds RF, Kurz X, de Groot MCH, *et al.* The IMI PROTECT project: purpose, organizational structure, and procedures. *Pharmacoepidemiol Drug Saf* 2016;**25 Suppl 1**:5–10. doi:10.1002/pds.3933

18 Coloma PM, Schuemie MJ, Trifirò G, *et al.* Combining electronic healthcare databases in Europe to allow for large-scale drug safety monitoring: the EU-ADR Project. *Pharmacoepidemiol Drug Saf* 2011;**20**:1–11. doi:10.1002/pds.2053

19 Trifiro G, Coloma PM, Rijnbeek PR, *et al.* Combining multiple healthcare databases for postmarketing drug and vaccine safety surveillance: why and how?. *J Intern Med* 2014;**275**:551–61. doi:10.1111/joim.12159

20 Mor A, Frøslev T, Thomsen RW, *et al.* Antibiotic use varies substantially among adults: a cross-national study from five European Countries in the ARITMO project. *Infection* 2015;**43**:453–72. doi:10.1007/s15010-015-0768-8

21 Abbing-Karahagopian V, Kurz X, de Vries F, *et al.* Bridging differences in outcomes of pharmacoepidemiological studies: design and first results of the PROTECT project. *Curr Clin Pharmacol* 2014;**9**:130–8.http://www.ncbi.nlm.nih.gov/pubmed/24218995

22 Hippisley-Cox J, Coupland C. Individualising the risks of statins in men and women in England and Wales: population-based cohort study. *Heart* 2010;**96**:939–47. doi:10.1136/hrt.2010.199034

23 Hippisley-Cox J, Coupland C. Development and validation of risk prediction equations to estimate future risk of blindness and lower limb amputation in patients with diabetes: cohort study. *BMJ* 2015;**351**:h5441. doi:10.1136/bmj.h5441

For peer review only

24 Clegg A, Bates C, Young J, *et al.* Development and validation of an electronic frailty index using routine primary care electronic health record data. *Age Ageing* 2016;**45**:353–60. doi:10.1093/ageing/afw039

25 Hippisley-Cox J, Coupland C. Predicting the risk of chronic Kidney Disease in men and women in England and Wales: prospective derivation and external validation of the QKidney Scores. *BMC Fam Pract* 2010;**11**:49. doi:10.1186/1471-2296-11-49

26 Hippisley-Cox J, Coupland C. Development and validation of risk prediction equations to estimate future risk of heart failure in patients with diabetes: a prospective cohort study. *BMJ Open* 2015;**5**:e008503. doi:10.1136/bmjopen-2015-008503

27 La Gamba F, Corrao G, Romio S, *et al.* Combining evidence from multiple electronic health care databases: performances of one-stage and two-stage meta-analysis in matched case-control studies. *Pharmacoepidemiol Drug Saf* 2017;**26**:1213–9. doi:10.1002/pds.4280

28 Trifirò G, de Ridder M, Sultana J, *et al.* Use of azithromycin and risk of ventricular arrhythmia. *CMAJ* 2017;**189**:E560–8. doi:10.1503/cmaj.160355

29 Masclee GMC, Valkhoff VE, Coloma PM, *et al.* Risk of upper gastrointestinal bleeding from different drug combinations. *Gastroenterology* 2014;**147**:784-792.e9. doi:10.1053/j.gastro.2014.06.007

30 Simmonds M, Stewart G, Stewart L. A decade of individual participant data meta-analyses: A review of current practice. *Contemp Clin Trials* 2015;**45**:76–83. doi:10.1016/j.cct.2015.06.012

31 Simmonds MC, Higgins JPT, Stewart LA, *et al.* Meta-analysis of individual patient data from randomized trials: a review of methods used in practice. *Clin Trials* 2005;**2**:209–17. doi:10.1191/1740774505cn087oa

32 Riley RD, Lambert PC, Abo-Zaid G. Meta-analysis of individual participant data: rationale, conduct, and reporting. *BMJ* 2010;**340**:c221. doi:10.1136/bmj.c221

33 Thomas D, Radji S, Benedetti A. Systematic review of methods for individual patient data meta- analysis with binary outcomes. *BMC Med Res Methodol* 2014;**14**:79. doi:10.1186/1471-2288-14-79

34 Mokhles MM, Trifirò G, Dieleman JP, *et al.* The risk of new onset heart failure associated with dopamine agonist use in Parkinson's disease. *Pharmacol Res* 2012;**65**:358–64. doi:10.1016/j.phrs.2011.11.009

35 Poluzzi E, Diemberger I, De Ridder M, *et al.* Use of antihistamines and risk of ventricular tachyarrhythmia: a nested case-control study in five European countries from the ARITMO project. *Eur J Clin Pharmacol* 2017;**73**:1499–510. doi:10.1007/s00228-017-2317-0

For peer review only

36 Austin PC, Merlo J. Intermediate and advanced topics in multilevel logistic regression analysis. *Stat Med* 2017;**36**:3257–77. doi:10.1002/sim.7336

37 Burke DL, Ensor J, Riley RD. Meta-analysis using individual participant data: one-stage and two-stage approaches, and why they may differ. *Stat Med* 2017;**36**:855–75. doi:10.1002/sim.7141

38 Debray TPA, Moons KGM, van Valkenhoef G, *et al.* Get real in individual participant data (IPD) meta-analysis: a review of the methodology. *Res Synth Methods* 2015;**6**:293–309. doi:10.1002/jrsm.1160

39 Debray TPA, Moons KGM, Abo-Zaid GMA, *et al.* Individual participant data meta-analysis for a binary outcome: one-stage or two-stage? *PLoS One* 2013;**8**:e60650. doi:10.1371/journal.pone.0060650

40 Stewart GB, Altman DG, Askie LM, *et al.* Statistical analysis of individual participant data meta-analyses: a comparison of methods and recommendations for practice. *PLoS One* 2012;**7**:e46042. doi:10.1371/journal.pone.0046042

41 Sweeting MJ, Sutton AJ, Lambert PC. What to add to nothing? Use and avoidance of continuity corrections in meta-analysis of sparse data. *Stat Med* 2004;**23**:1351–75. doi:10.1002/sim.1761

42 Kumarapeli P, de Lusignan S. Using the computer in the clinical consultation; setting the stage, reviewing, recording, and taking actions: multi-channel video study. *J Am Med Inform Assoc* 2013;**20**:e67-75. doi:10.1136/amiajnl-2012-001081

43 De Clercq E, Van Casteren V, Jonckheer P, *et al.* Electronic Patient Record data as proxy of GPs' thoughts. *Stud Health Technol Inform* 2008;**141**:103–10. doi:10.3233/978-1-58603-922-6-103

44 Tai TW, Anandarajah S, Dhoul N, *et al.* Variation in clinical coding lists in UK general practice: a barrier to consistent data entry? *Inform Prim Care* 2007;**15**:143–50. doi:10.14236/jhi.v15i3.652

45 De Wilde S, Carey IM, Bremner SA, *et al.* A comparison of the recording of 30 common childhood conditions in the Doctor's Independent Network and General Practice Research Databases. *Heal Stat Q* 2004;:21–31.http://www.ncbi.nlm.nih.gov/pubmed/15704391

46 Sollie A, Roskam J, Sijmons RH, *et al.* Do GPs know their patients with cancer? Assessing the quality of cancer registration in Dutch primary care: a cross-sectional validation study. *BMJ Open* 2016;**6**:e012669. doi:10.1136/bmjopen-2016-012669

47 Carey IM, Cook DG, De Wilde S, *et al.* Implications of the problem orientated medical record (POMR) for research using electronic GP databases: a comparison of the Doctors Independent Network Database (DIN) and the General Practice Research Database

For peer review only

(GPRD). *BMC Fam Pract* 2003;**4**:14. doi:10.1186/1471-2296-4-14

48 Borenstein M, Hedges L V, Higgins JPT, *et al.* A basic introduction to fixed-effect and random-effects models for meta-analysis. *Res Synth Methods* 2010;**1**:97–111. doi:10.1002/jrsm.12

49 Kontopantelis E, Springate DA, Reeves D. A re-analysis of the Cochrane Library data: the dangers of unobserved heterogeneity in meta-analyses. *PLoS One* 2013;**8**:e69930. doi:10.1371/journal.pone.0069930

50 Thorlund K, Imberger G, Johnston BC, *et al.* Evolution of heterogeneity (I2) estimates and their 95% confidence intervals in large meta-analyses. *PLoS One* 2012;**7**:e39471. doi:10.1371/journal.pone.0039471

51 von Hippel PT. The heterogeneity statistic I(2) can be biased in small meta-analyses. *BMC Med Res Methodol* 2015;**15**:35. doi:10.1186/s12874-015-0024-z

52 Vinogradova Y, Coupland C, Hippisley-Cox J. Use of combined oral contraceptives and risk of venous thromboembolism: nested case-control studies using the QResearch and CPRD databases. *BMJ* 2015;**350**:h2135. doi:10.1136/bmj.h2135

53 Thomas SL, Minassian C, Ganesan V, *et al.* Chickenpox and risk of stroke: A self-controlled case series analysis. *Clin Infect Dis* 2014;**58**:61–8. doi:10.1093/cid/cit659

54 Vinogradova Y, Coupland C, Hippisley-Cox J. Exposure to bisphosphonates and risk of common non-gastrointestinal cancers: series of nested case-control studies using two primary-care databases. *Br J Cancer* 2013;**109**:795–806. doi:10.1038/bjc.2013.383

55 Vinogradova Y, Coupland C, Hippisley-Cox J. Exposure to bisphosphonates and risk of gastrointestinal cancers: series of nested case-control studies with QResearch and CPRD data. *BMJ* 2013;**346**:f114. doi:10.1136/bmj.f114

56 Bremner SA, Carey IM, DeWilde S, *et al.* Infections presenting for clinical care in early life and later risk of hay fever in two UK birth cohorts. *Allergy* 2008;**63**:274–83. doi:10.1111/j.1398-9995.2007.01599.x

57 Bremner SA, Carey IM, DeWilde S, *et al.* Early-life exposure to antibacterials and the subsequent development of hayfever in childhood in the UK: case-control studies using the General Practice Research Database and the Doctors' Independent Network. *Clin Exp Allergy* 2003;**33**:1518–25. doi:10.1046/j.1365-2222.2003.01794.x

58 Bremner SA, Carey IM, DeWilde S, *et al.* Timing of routine immunisations and subsequent hay fever risk. *Arch Dis Child* 2005;**90**:567–73. doi:10.1136/adc.2004.051714

59 Bremner SA, Carey IM, DeWilde S, *et al.* Vaccinations, infections and antibacterials in the first grass pollen season of life and risk of later hayfever. *Clin Exp Allergy* 2007;**37**:512–7.

For peer review only

doi:10.1111/j.1365-2222.2007.02697.x

60 Gonnermann A, Framke T, Großhennig A, *et al.* No solution yet for combining two independent studies in the presence of heterogeneity. *Stat Med* 2015;**34**:2476–80. doi:10.1002/sim.6473

61 Röver C, Knapp G, Friede T. Hartung-Knapp-Sidik-Jonkman approach and its modification for random-effects meta-analysis with few studies. *BMC Med Res Methodol* 2015;**15**:99. doi:10.1186/s12874-015-0091-1

62 IntHout J, Ioannidis JPA, Borm GF. The Hartung-Knapp-Sidik-Jonkman method for random effects meta-analysis is straightforward and considerably outperforms the standard DerSimonian-Laird method. *BMC Med Res Methodol* 2014;**14**:25. doi:10.1186/1471-2288-14-25

63 Friede T, Röver C, Wandel S, *et al.* Meta-analysis of two studies in the presence of heterogeneity with applications in rare diseases. *Biom J* 2017;**59**:658–71. doi:10.1002/bimj.201500236

64 Doi SAR, Barendregt JJ, Khan S, *et al.* Advances in the meta-analysis of heterogeneous clinical trials I: The inverse variance heterogeneity model. *Contemp Clin Trials* 2015;**45**:130–8. doi:10.1016/j.cct.2015.05.009

65 Stanley TD, Doucouliagos H. Neither fixed nor random: weighted least squares meta-regression. *Res Synth Methods* 2017;**8**:19–42. doi:10.1002/jrsm.1211

66 Seide SE, Röver C, Friede T. Likelihood-based random-effects meta-analysis with few studies: empirical and simulation studies. *BMC Med Res Methodol* 2019;**19**:16. doi:10.1186/s12874-018-0618-3

67 Friede T, Röver C, Wandel S, *et al.* Meta-analysis of two studies in the presence of heterogeneity with applications in rare diseases. *Biom J* 2017;**59**:658–71. doi:10.1002/bimj.201500236

68 Selmer R, Haglund B, Furu K, *et al.* Individual-based versus aggregate meta-analysis in multi-database studies of pregnancy outcomes: the Nordic example of selective serotonin reuptake inhibitors and venlafaxine in pregnancy. *Pharmacoepidemiol Drug Saf* 2016;**25**:1160–9. doi:10.1002/pds.4033

69 Pacurariu A, Plueschke K, McGettigan P, *et al.* Electronic healthcare databases in Europe: descriptive analysis of characteristics and potential for use in medicines regulation. *BMJ Open* 2018;**8**:e023090. doi:10.1136/bmjopen-2018-023090

70 Benchimol EI, Smeeth L, Guttmann A, *et al.* The REporting of studies Conducted using Observational Routinely-collected health Data (RECORD) statement. *PLoS Med* 2015;**12**:e1001885. doi:10.1371/journal.pmed.1001885

For peer review only

For peer review only

Footnotes

Author statement

DD: study concept and design, including search strategy; data extraction proformas and study database design; screening of titles and abstracts, and data extraction; analysis; interpretation of results; drafting of manuscript.

MC: screening of titles and abstracts, and data extraction; interpretation of results, critical review of manuscript, and approval of final version.

RW: study concept and design, including search strategy; interpretation of results, critical review of manuscript, and approval of final version.

SE: interpretation of results, critical review of manuscript, and approval of final version.

KB: study concept and design, including search strategy; interpretation of results, critical review of manuscript, and approval of final version.

ID: study concept and design, including search strategy; interpretation of results, critical review of manuscript, and approval of final version.

Acknowledgements

We would like to thank Russell Burke (Information Services, London School of Hygiene and Tropical Medicine) for his advice on bibliographic search strategies.

Funding

This research was conducted as part of a postgraduate doctoral degree funded by the Clinical Practice Research Datalink. KB holds a Sir Henry Dale Fellowship funded by Wellcome and the Royal Society (grant number 107731/Z/15/Z).

Competing interests

None

Ethics approval

Not required

Patient consent for publication

Not required

Data sharing statement

The study database and data-extraction proformas are available on request from DD.

For peer review only

Figure 1: Selection and inclusion of studies for systematic review

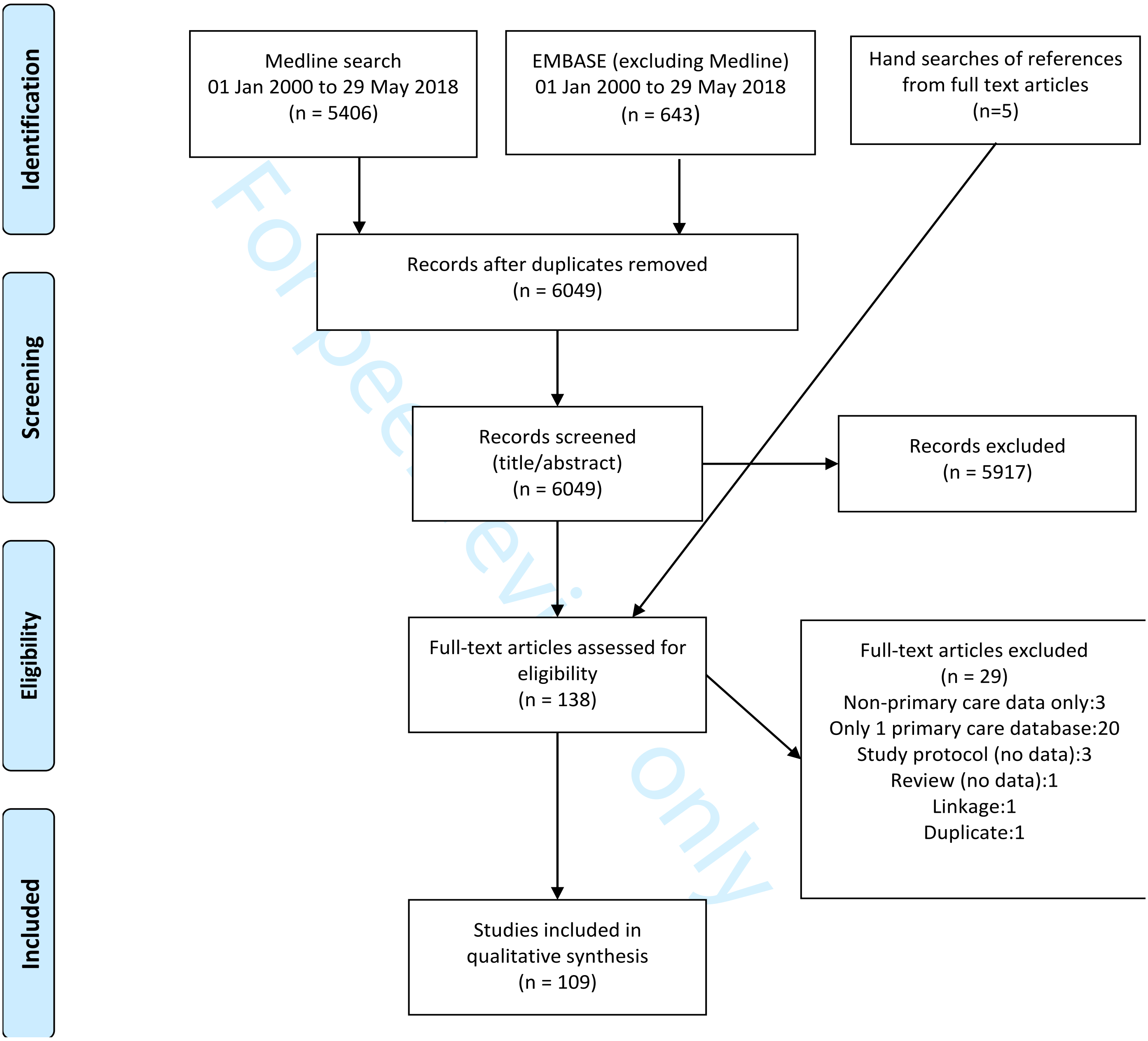

Adapted from: Moher D, Liberati A, Tetzlaff J, Altman DG, The PRISMA Group (2009). *P*referred *R*eporting *I*tems for *S*ystematic Reviews and *M*eta-*A*nalyses: The PRISMA Statement. PLoS Med 6(7): e1000097. doi:10.1371/journal.pmed1000097

For peer review only

Figure 2: Comparison of relative risk (RR) estimates reported in studies using 2 or more methods to combine data from multiple sources

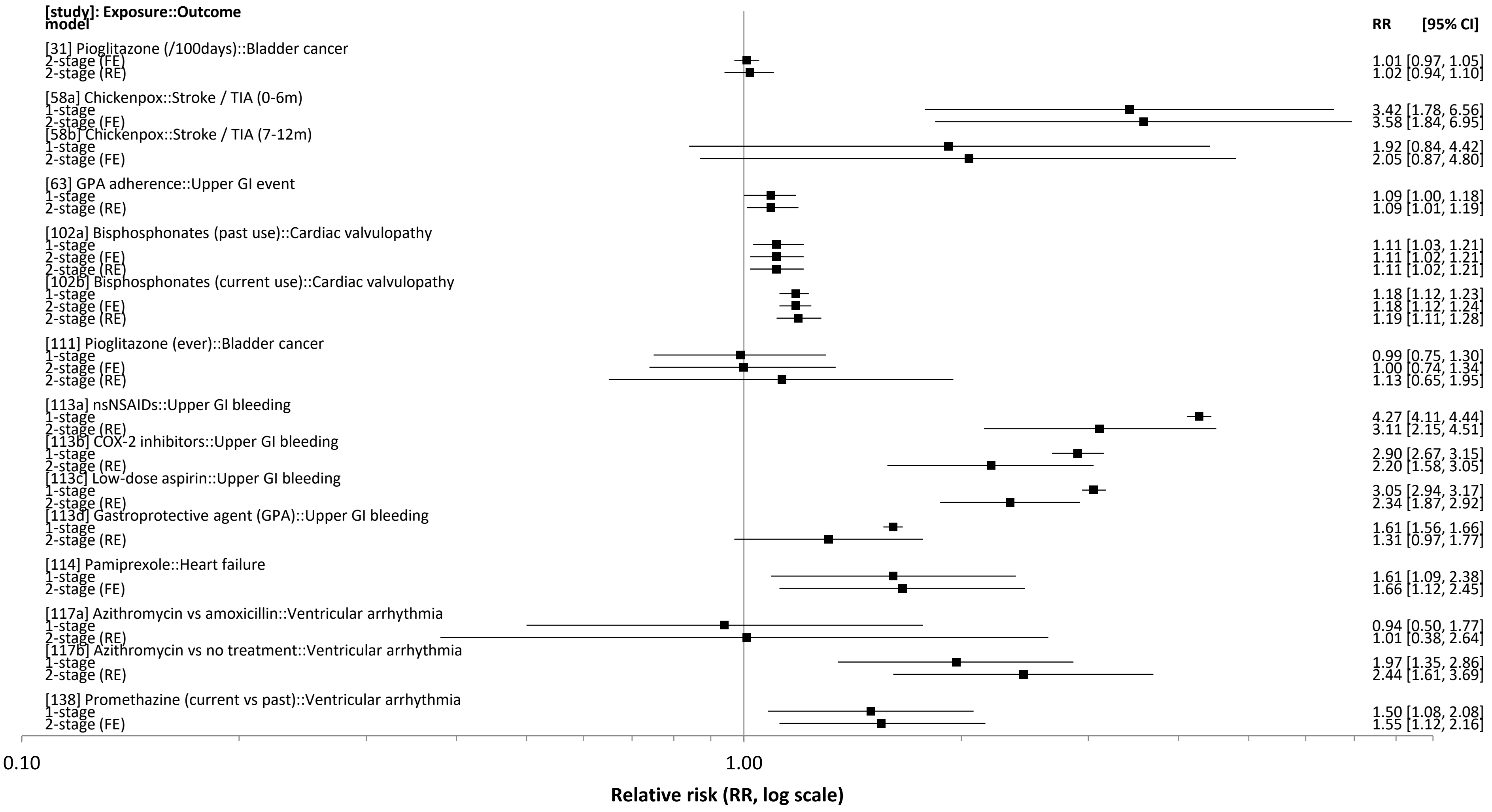

[study] refers to the review database id number (see Supplementary table S1)

Multidatabase Systematic Review: Supplementary Tables and Figures

Figure S1: Number of included studies by year of publication

Table S1: Summary details of 109 studies included in systematic review

Table S2: Summary details of primary care electronic health record (EHR) data sources used in studies included in systematic review

For peer review only

Figure S1: Number of included studies by year of publication

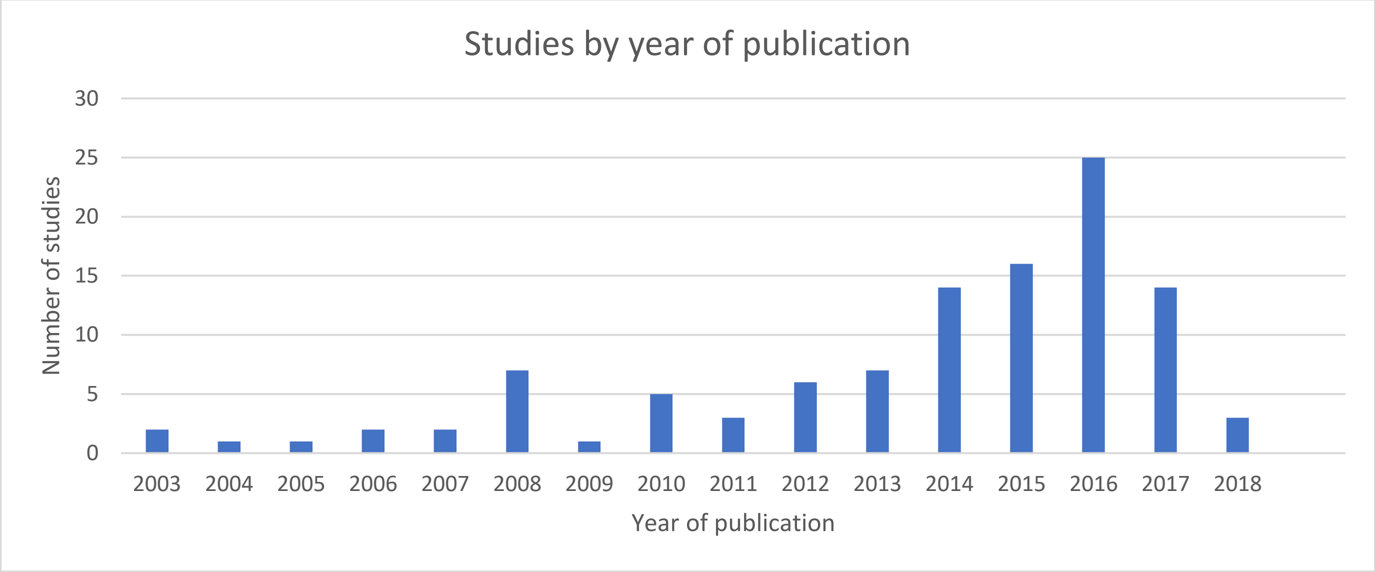

peer review only

Table S1: Summary details of 109 studies included in systematic review.

Study ID[a]	Citation	Resource locator: [doi unless specified otherwise][b]	Study topic	Study type	Study design[c]	Data combining	Primary care EHR data sources[d]	Other sources (if applicable)
1	Lum KJ, Newcomb CW, Roy JA, et al. Evaluation of methods to estimate missing days' supply within pharmacy data of the Clinical Practice Research Datalink (CPRD) and The Health Improvement Network (THIN). Eur J Clin Pharmacol 2017;73:115–23	10.1007/s00228-016-2148-4	Drug utilization	Other	Other	Data not combined	2: CPRD; THIN	
2	de Bie S, Kaguelidou F, Verhamme KMC, et al. Using Prescription Patterns in Primary Care to Derive New Quality Indicators for Childhood Community Antibiotic Prescribing. Pediatr Infect Dis J 2016;35:1317–23	10.1097/INF.0000000000001324	Drug utilization	Descriptive	Cohort	Data not combined	3: IPCI; Pedianet; THIN	
3	Masclee GMC, Coloma PM, Spaander MCW, et al. NSAIDs, statins, low-dose aspirin and PPIs, and the risk of oesophageal adenocarcinoma among patients with Barrett's oesophagus: a population-based Case-control. BMJ Open 2015;5:e006640	10.1136/bmjopen-2014-006640	Drug safety	Analytical	Case-control	Pooled analysis: individual patient data	2: IPCI; THIN	
7	Laforest L, Licaj I, Devouassoux G, et al. Prescribed therapy for asthma: therapeutic ratios and outcomes. BMC Fam Pract 2015;16:49	10.1186/s12875-015-0265-2	Drug comparative effectiveness	Analytical	Cohort	Data not combined	2: LPD (France); THIN	
8	Afonso A, Schmiedl S, Becker C, et al. A methodological comparison of two European primary care databases and replication in a US claims database: inhaled long-acting beta-2-agonists and the risk of acute myocardial infarction. Eur J Clin Pharmacol 2016;72:1105–16	10.1007/s00228-016-2071-8	Drug safety	Analytical	Cohort	Data not combined	2: CPRD; NPCRD (Mondriaan)	1
9	Ali MS, Groenwold RHH, Belitser S V, et al. Methodological comparison of marginal structural model, time-varying Cox regression, and propensity score methods: the example of antidepressant use and the risk of hip fracture. Pharmacoepidemiol Drug Saf 2016;25 Suppl 1:114–21	10.1002/pds.3864	Drug safety	Analytical	Cohort	Data not combined	3: AHC (Mondriaan); BIFAP; NPCRD (Mondriaan)	
10	Bezemer ID, Verhamme KMC, Gini R, et al. Use of oral contraceptives in three European countries: a population-based multi-database study. Eur J Contracept Reprod Health Care 2016;21:81–7	10.3109/13625187.2015.1102220	Drug utilization	Descriptive	Cohort	Data not combined	3: HSD (Italy); IPCI; THIN	1
11	Brauer R, Douglas I, Garcia Rodriguez LA, et al. Risk of acute liver injury associated with use of antibiotics. Comparative cohort and nested case-control studies using two primary care databases in Europe. Pharmacoepidemiol Drug Saf 2016;25 Suppl 1:29–38	10.1002/pds.3861	Drug safety	Analytical	Multiple: case-control; cohort	Data not combined	2: BIFAP; CPRD	
12	Brauer R, Ruigómez A, Downey G, et al. Prevalence of antibiotic use: a comparison across various European health care data sources. Pharmacoepidemiol Drug Saf 2016;25 Suppl 1:11–20	10.1002/pds.3831	Drug utilization	Descriptive	Cross-sectional	Data not combined	5: AHC (Mondriaan); BIFAP; CPRD; NPCRD (Mondriaan); THIN	2
14	Castellsague J, Perez-Gutthann S, Calingaert B, et al. Characterization of new users of cilostazol in the UK, Spain, Sweden, and Germany. Pharmacoepidemiol Drug Saf 2017;26:615–24	10.1002/pds.4167	Drug utilization	Descriptive	Cohort	Data not combined	2: SIDIAP; THIN	3
15	Charlton RA, Pierini A, Klungsøyr K, et al. Asthma medication prescribing before, during and after pregnancy: a study in seven European regions. BMJ Open 2016;6:e009237	10.1136/bmjopen-2015-009237	Drug utilization	Descriptive	Cohort	Data not combined	2: CPRD; SAIL	5
16	Charlton R, Garne E, Wang H, et al. Antiepileptic drug prescribing before, during and after pregnancy: a study in seven European regions. Pharmacoepidemiol Drug Saf 2015;24:1144–54	10.1002/pds.3847	Drug utilization	Descriptive	Cohort	Data not combined	2: CPRD; SAIL	5
17	Chui CSL, Chan EW, Wong AYS, et al. Association between oral fluoroquinolones and seizures: A self-controlled case series study. Neurology 2016;86:1708–15	10.1212/WNL.0000000000002633	Drug safety	Analytical	SCCS	Meta-analysis: random effects	2: CDARS; CPRD	
18	Clegg A, Bates C, Young J, et al. Development and validation of an electronic frailty index using routine primary care electronic health record data. Age Ageing 2016;45:353–60	10.1093/ageing/afw039	Disease risk prediction	Analytical	Cohort	Data not combined	2: ResearchOne; THIN	
19	De Bortoli N, Ripellino C, Cataldo N, et al. Unspecified intestinal malabsorption in patients treated with angiotensin-converting enzyme inhibitors or angiotensin receptor blockers: a retrospective analysis in primary care settings. Expert Opin Drug Saf 2017;16:1221–5	10.1080/14740338.2017.1376647	Drug safety	Descriptive	Cohort	Pooled analysis: individual patient data	2: Disease Analyzer (Germany); HSD (Italy)	

For peer review only - http://bmjopen.bmj.com/site/about/guidelines.xhtml

Study ID[a]	Citation	Resource locator: [doi unless specified otherwise][b]	Study topic	Study type	Study design[c]	Data combining	Primary care EHR data sources[d]	Other sources (if applicable)
20	de Groot MCH, Candore G, Uddin MJ, et al. Case-only designs for studying the association of antidepressants and hip or femur fracture. Pharmacoepidemiol Drug Saf 2016;25 Suppl 1:103–13	10.1002/pds.3850	Drug safety	Analytical	Multiple: CCX; SCCS	Data not combined	3: AHC (Mondriaan); NPCRD (Mondriaan); THIN	
24	Ferrajolo C, Verhamme KMC, Trifirò G, et al. Antibiotic-Induced Liver Injury in Paediatric Outpatients: A Case-control in Primary Care Databases. Drug Saf 2017;40:305–15	10.1007/s40264-016-0493-y	Drug safety	Analytical	Case-control	Pooled analysis: individual patient data	3: HSD (Italy); IPCI; Pedianet	
25	Gold R, Esterberg E, Hollombe C, et al. Low Back Imaging When Not Indicated: A Descriptive Cross-System Analysis. Perm J 2016;20:25–33	10.7812/TPP/15-081	Health services research	Analytical	Cohort	Pooled analysis: individual patient data	2: KP Clarity (Epic EHR); OCHIN Clarity (Epic EHR)	
26	Hippisley-Cox J, Coupland C. Development and validation of risk prediction equations to estimate future risk of blindness and lower limb amputation in patients with diabetes: Cohort. BMJ 2015;351:h5441	10.1136/bmj.h5441	Disease risk prediction	Analytical	Cohort	Data not combined	2: CPRD; QResearch	
27	Hippisley-Cox J, Coupland C. Development and validation of risk prediction equations to estimate future risk of heart failure in patients with diabetes: a prospective Cohort. BMJ Open 2015;5:e008503	10.1136/bmjopen-2015-008503	Disease risk prediction	Analytical	Cohort	Data not combined	2: CPRD; QResearch	
28	Israel E, Roche N, Martin RJ, et al. Increased Dose of Inhaled Corticosteroid versus Add-On Long-acting ß-Agonist for Step-Up Therapy in Asthma. Ann Am Thorac Soc 2015;12:798–806	10.1513/AnnalsATS.201412-580OC	Drug comparative effectiveness	Analytical	Cohort	Pooled analysis: individual patient data	2: CPRD; OPCRD	
30	Levi M, Rosselli M, Simonetti M, et al. Epidemiology of iron deficiency anaemia in four European countries: a population-based study in primary care. Eur J Haematol 2016;97:583–93	10.1111/ejh.12776	Disease epidemiology	Descriptive	Cohort	Data not combined	4: HSD (Italy); LPD (Belgium); LPD (Germany); LPD (Spain)	
31	Levin D, Bell S, Sund R, et al. Pioglitazone and bladder cancer risk: a multipopulation pooled, cumulative exposure analysis. Diabetologia 2015;58:493–504	10.1007/s00125-014-3456-9	Drug safety	Analytical	Cohort	Meta-analysis: fixed & random effects	2: CPRD; SIR	4
32	Murray CS, Thomas M, Richardson K, et al. Comparative Effectiveness of Step-up Therapies in Children with Asthma Prescribed Inhaled Corticosteroids: A Historical Cohort. J allergy Clin Immunol Pract 2017;5:1082-1090.e7	10.1016/j.jaip.2016.12.28	Drug comparative effectiveness	Analytical	Cohort	Pooled analysis: individual patient data	2: CPRD; OPCRD	
33	Nyberg F, Horne L, Morlock R, et al. Comorbidity Burden in Trial-Aligned Patients with Established Gout in Germany, UK, US, and France: a Retrospective Analysis. Adv Ther 2016;33:1180–98	10.1007/s12325-016-0346-1	Disease epidemiology	Descriptive	Cohort	Data not combined	3: CPRD; Disease Analyzer (France); Disease Analyzer (Germany)	1
34	Oteri A, Mazzaglia G, Pecchioli S, et al. Prescribing pattern of antipsychotic drugs during the years 1996-2010: a population-based database study in Europe with a focus on torsadogenic drugs. Br J Clin Pharmacol 2016;82:487–97	10.1111/bcp.12955	Drug utilization	Descriptive	Cohort	Data not combined	3: HSD (Italy); IPCI; THIN	4
35	Petersen I, McCrea RL, Sammon CJ, et al. Risks and benefits of psychotropic medication in pregnancy: cohort studies based on UK electronic primary care health records. Health Technol Assess 2016;20:1–176	10.3310/hta20230	Drug safety	Analytical	Cohort	Pooled analysis: individual patient data	2: CPRD; THIN	
36	Petherick ES, Pickett KE, Cullum NA. Can different primary care databases produce comparable estimates of burden of disease: results of a study exploring venous leg ulceration. Fam Pract 2015;32:374–80	10.1093/fampra/cmv013	Disease epidemiology	Descriptive	Cohort	Data not combined	2: CPRD; THIN	
37	Rathmann W, Czech M, Franek E, et al. Regional differences in insulin therapy regimens in five European countries. Int J Clin Pharmacol Ther 2017;55:403–8	10.5414/CP202906	Drug utilization	Descriptive	Cross-sectional	Data not combined	3: Disease Analyzer (France); Disease Analyzer (Germany); Disease Analyzer (UK)	2
38	Requena G, Huerta C, Gardarsdottir H, et al. Hip/femur fractures associated with the use of benzodiazepines (anxiolytics, hypnotics and related drugs): a methodological approach to assess consistencies across databases from the PROTECT-EU project. Pharmacoepidemiol Drug Saf 2016;25 Suppl 1:66–78	10.1002/pds.3816	Drug safety	Analytical	Multiple: case-control; cohort	Meta-analysis: random effects	3: BIFAP; CPRD; NPCRD (Mondriaan)	
39	Requena G, Logie J, Martin E, et al. Do case-only designs yield consistent results across design and different databases? A case study of hip fractures and benzodiazepines. Pharmacoepidemiol Drug Saf 2016;25 Suppl 1:79–87	10.1002/pds.3822	Drug safety	Analytical	Multiple: CCX;SCCS	Data not combined	2: BIFAP; CPRD	

For peer review only

Study ID[a]	Citation	Resource locator: [doi unless specified otherwise][b]	Study topic	Study type	Study design[c]	Data combining	Primary care EHR data sources[d]	Other sources (if applicable)
40	Saine ME, Carbonari DM, Newcomb CW, et al. Determinants of saxagliptin use among patients with type 2 diabetes mellitus treated with oral anti-diabetic drugs. BMC Pharmacol Toxicol 2015;16:8	10.1186/s40360-015-0007-z	Drug utilization	Analytical	Cross-sectional	Data not combined	2: CPRD; THIN	2
41	Souverein PC, Abbing-Karahagopian V, Martin E, et al. Understanding inconsistency in the results from observational pharmacoepidemiological studies: the case of antidepressant use and risk of hip/femur fractures. Pharmacoepidemiol Drug Saf 2016;25 Suppl 1:88–102	10.1002/pds.3862	Drug safety	Analytical	Multiple: case-control; cohort	Data not combined	4: AHC (Mondriaan); BIFAP; NPCRD (Mondriaan); THIN	
43	Sultana J, Fontana A, Giorgianni F, et al. The Effect of Safety Warnings on Antipsychotic Drug Prescribing in Elderly Persons with Dementia in the United Kingdom and Italy: A Population-Based Study. CNS Drugs 2016;30:1097–109	10.1007/s40263-016-0366-z	Health services research	Analytical	Interrupted time series	Data not combined	2: HSD (Italy); THIN	
44	Turner SW, Richardson K, Burden A, et al. Initial step-up treatment changes in asthmatic children already prescribed inhaled corticosteroids: a historical Cohort. NPJ Prim care Respir Med 2015;25:15041	10.1038/npjpcrm.2015.41	Drug utilization	Descriptive	Cohort	Pooled analysis: individual patient data	2: CPRD; OPCRD	
45	Turner S, Richardson K, Murray C, et al. Long-Acting ß-Agonist in Combination or Separate Inhaler as Step-Up Therapy for Children with Uncontrolled Asthma Receiving Inhaled Corticosteroids. J allergy Clin Immunol Pract 2017;5:99-106.e3	10.1016/j.jaip.2016.06.9	Drug comparative effectiveness	Analytical	Cohort	Pooled analysis: individual patient data	2: CPRD; OPCRD	
46	Tyrstrup M, van der Velden A, Engstrom S, et al. Antibiotic prescribing in relation to diagnoses and consultation rates in Belgium, the Netherlands and Sweden: use of European quality indicators. Scand J Prim Health Care 2017;35:10–8	10.1080/02813432.2017.1288680	Health services research	Descriptive	Cross-sectional	Data not combined	3: Intego; Jonkoping County; Julius (Mondriaan)	
47	Uddin MJ, Groenwold RHH, de Boer A, et al. Evaluating different physician's prescribing preference based instrumental variables in two primary care databases: a study of inhaled long-acting beta2-agonist use and the risk of myocardial infarction. Pharmacoepidemiol Drug Saf 2016;25 Suppl 1:132–41	10.1002/pds.3860	Drug safety	Analytical	Cohort	Pooled analysis: individual patient data	2: CPRD; NPCRD (Mondriaan)	
48	Uddin MJ, Groenwold RHH, de Boer A, et al. Instrumental variables analysis using multiple databases: an example of antidepressant use and risk of hip fracture. Pharmacoepidemiol Drug Saf 2016;25 Suppl 1:122–31	10.1002/pds.3863	Drug safety	Analytical	Cohort	Pooled analysis: individual patient data	3: BIFAP; NPCRD (Mondriaan); THIN	
49	Vinogradova Y, Coupland C, Hippisley-Cox J. Use of combined oral contraceptives and risk of venous thromboembolism: nested case-control studies using the QResearch and CPRD databases. BMJ 2015;350:h2135	10.1136/bmj.h2135	Drug safety	Analytical	Case-control	Meta-analysis: fixed & random effects	2: CPRD; QResearch	
50	Hippisley-Cox J, Coupland C, Brindle P. The performance of seven QPrediction risk scores in an independent external sample of patients from general practice: a validation study. BMJ Open 2014;4:e005809	10.1136/bmjopen-2014-005809	Disease risk prediction	Descriptive	Cohort	Data not combined	2: CPRD; QResearch	
51	Kostev K, Jockwig A, Hallwachs A, et al. Prevalence and risk factors of neuropathy in newly diagnosed type 2 diabetes in primary care practices: a retrospective database analysis in Germany and UK. Prim Care Diabetes 2014;8:250–5	10.1016/j.pcd.2014.01.11	Disease epidemiology	Analytical	Cohort	Data not combined	2: Disease Analyzer (Germany); Disease Analyzer (UK)	
52	Kostev K, Rathmann W. Influence of macro- and microvascular comorbidity on time to insulin initiation in type 2 diabetes patients: a retrospective database analysis in Germany, France, and UK. Prim Care Diabetes 2013;7:167–71	10.1016/j.pcd.2013.02.001	Drug utilization	Analytical	Cohort	Data not combined	3: Disease Analyzer (France); Disease Analyzer (Germany); Disease Analyzer (UK)	
54	Masclee GMC, Coloma PM, de Wilde M, et al. The incidence of Barrett's oesophagus and oesophageal adenocarcinoma in the United Kingdom and The Netherlands is levelling off. Aliment Pharmacol Ther 2014;39:1321–30	10.1111/apt.12759	Disease epidemiology	Descriptive	Cohort	Data not combined	2: IPCI; THIN	
55	Masclee GMC, Valkhoff VE, van Soest EM, et al. Cyclo-oxygenase-2 inhibitors or nonselective NSAIDs plus gastroprotective agents: what to prescribe in daily clinical practice?.Aliment Pharmacol Ther 2013;38:178–89	10.1111/apt.12348	Drug safety	Analytical	Case-control	Multiple: pooled IPD + meta-analysis (RE)	3: CPRD; HSD (Italy); IPCI	
56	de Jonge L, Garne E, Gini R, et al. Improving Information on Maternal Medication Use by Linking Prescription Data to Congenital Anomaly Registers: A EUROmediCAT Study. Drug Saf 2015;38:1083–93	10.1007/s40264-015-0321-9	Disease epidemiology	Descriptive	Cohort	Data not combined	5: AHC (Mondriaan); BIFAP; CPRD; NPCRD (Mondriaan); THIN	2

For peer review only

Study ID[a]	Citation	Resource locator: [doi unless specified otherwise][b]	Study topic	Study type	Study design[c]	Data combining	Primary care EHR data sources[d]	Other sources (if applicable)
57	Ruigómez A, Brauer R, Rodríguez LAG, et al. Ascertainment of acute liver injury in two European primary care databases. Eur J Clin Pharmacol 2014;70:1227–35	10.1007/s00228-014-1721-y	Disease epidemiology	Descriptive	Cohort	Data not combined	2: BIFAP; CPRD	
58	Thomas SL, Minassian C, Ganesan V, et al. Chickenpox and risk of stroke: A self-controlled case series analysis. Clin Infect Dis 2014;58:61–8	10.1093/cid/cit659	Disease epidemiology	Analytical	SCCS	Multiple: pooled IPD + meta-analysis (RE + FE)	4: CPRD; Disease Analyzer (UK); QResearch; THIN	
59	Trifirò G, Mokhles MM, Dieleman JP, et al. Risk of cardiac valve regurgitation with dopamine agonist use in Parkinson's disease and hyperprolactinaemia: a multi-country, nested Case-control. Drug Saf 2012;35:159–71	10.2165/11594940-000000000-00000	Drug safety	Analytical	Case-control	Pooled analysis: individual patient data	3: HSD (Italy); IPCI; THIN	
60	Valkhoff VE, van Soest EM, Masclee GMC, et al. Prescription of nonselective NSAIDs, coxibs and gastroprotective agents in the era of rofecoxib withdrawal - a 617,400-patient study. Aliment Pharmacol Ther 2012;36:790–9	10.1111/apt.12028	Drug utilization	Descriptive	Cohort	Data not combined	3: CPRD; HSD (Italy); IPCI	
61	Valkhoff VE, Coloma PM, Masclee GMC, et al. Validation study in four health-care databases: Upper gastrointestinal bleeding misclassification affects precision but not magnitude of drug-related upper gastrointestinal bleeding risk. J Clin Epidemiol 2014;67:921–31	10.1016/j.jclinepi.2014.02.020	Methodology / data quality	Analytical	Cohort	Pooled analysis: individual patient data	2: HSD (Italy); IPCI	2
62	Valkhoff VE, Schade R, 't Jong GW, et al. Population-based analysis of non-steroidal anti-inflammatory drug use among children in four European countries in the SOS project: what size of data platforms and which study designs do we need to assess safety issues?BMC Pediatr 2013;13:192	10.1186/1471-2431-13-192	Drug utilization	Descriptive	Cohort	Pooled analysis: semi-aggregate data	3: IPCI; Pedianet; THIN	4
63	Valkhoff VE, van Soest EM, Mazzaglia G, et al. Adherence to gastroprotection during cyclooxygenase 2 inhibitor treatment and the risk of upper gastrointestinal tract events: a population-based study. Arthritis Rheum 2012;64:2792–802	10.1002/art.34433	Drug safety	Analytical	Case-control	Multiple: pooled IPD + meta-analysis (RE)	3: CPRD; HSD (Italy); IPCI	
64	Vinogradova Y, Coupland C, Hippisley-Cox J. Exposure to bisphosphonates and risk of common non-gastrointestinal cancers: series of nested case-control studies using two primary-care databases. Br J Cancer 2013;109:795–806	10.1038/bjc.2013.383	Drug safety	Analytical	Case-control	Meta-analysis: fixed effects	2: CPRD; QResearch	
66	Vinogradova Y, Coupland C, Hippisley-Cox J. Exposure to bisphosphonates and risk of gastrointestinal cancers: series of nested case-control studies with QResearch and CPRD data. BMJ 2013;346:f114	10.1136/bmj.f114	Drug safety	Analytical	Case-control	Meta-analysis: fixed effects	2: CPRD; QResearch	
67	Bremner SA, Carey IM, DeWilde S, et al. Infections presenting for clinical care in early life and later risk of hay fever in two UK birth cohorts. Allergy 2008;63:274–83	10.1111/j.1398-9995.2007.01599.x	Disease epidemiology	Analytical	Case-control	Meta-analysis: fixed effects	2: CPRD; DIN	
68	Cooper C, Steinbuch M, Stevenson R, et al. The epidemiology of osteonecrosis: findings from the GPRD and THIN databases in the UK. Osteoporos Int 2010;21:569–77	10.1007/s00198-009-1003-1	Disease epidemiology	Analytical	Case-control	Pooled analysis: individual patient data	2: CPRD; THIN	
69	De Clercq E, Van Casteren V, Jonckheer P, et al. Electronic Patient Record data as proxy of GPs' thoughts. Stud Health Technol Inform 2008;141:103–10	10.3233/978-1-58603-922-6-103	Methodology / data quality	Descriptive	Other	Data not combined	3: Resoprim (x3 networks)	
70	Hippisley-Cox J, Coupland C, Vinogradova Y, et al. Performance of the QRISK cardiovascular risk prediction algorithm in an independent UK sample of patients from general practice: a validation study. Heart 2008;94:34–9	10.1136/hrt.2007.134890	Disease risk prediction	Descriptive	Cohort	Data not combined	2: QResearch; THIN	
71	Hippisley-Cox J, Coupland C. Individualising the risks of statins in men and women in England and Wales: population-based Cohort. Heart 2010;96:939–47	10.1136/hrt.2010.199034	Disease risk prediction	Analytical	Cohort	Data not combined	2: QResearch; THIN	
72	Hippisley-Cox J, Coupland C. Predicting the risk of chronic Kidney Disease in men and women in England and Wales: prospective derivation and external validation of the QKidney Scores. BMC Fam Pract 2010;11:49	10.1186/1471-2296-11-49	Disease risk prediction	Analytical	Cohort	Data not combined	2: QResearch; THIN	
73	Hsia Y, Neubert A, Sturkenboom MCJM, et al. Comparison of antiepileptic drug prescribing in children in three European countries. Epilepsia 2010;51:789–96	10.1111/j.1528-1167.2009.02331.x	Drug utilization	Descriptive	Cohort	Pooled analysis: semi-aggregate data	3: Disease Analyzer (UK); IPCI; Pedianet	
74	Molokhia M, McKeigue P, Curcin V, et al. Statin induced myopathy and myalgia: time trend analysis and comparison of risk associated with statin class from 1991-2006. PLoS One 2008;3:e2522	10.1371/journal.pone.0002522	Drug safety	Analytical	CCX	Data not combined	2: Mediplus; THIN	

For peer review only

Study ID[a]	Citation	Resource locator: [doi unless specified otherwise][b]	Study topic	Study type	Study design[c]	Data combining	Primary care EHR data sources[d]	Other sources (if applicable)
75	Neubert A, Verhamme K, Murray ML, et al. The prescribing of analgesics and non-steroidal anti-inflammatory drugs in paediatric primary care in the UK, Italy and the Netherlands. Pharmacol Res 2010;62:243–8	10.1016/j.phrs.2010.04.006	Drug utilization	Descriptive	Cohort	Data not combined	3: Disease Analyzer (UK); IPCI; Pedianet	
76	Pfeil N, Uhlig U, Kostev K, et al. Antiemetic medications in children with presumed infectious gastroenteritis--pharmacoepidemiology in Europe and Northern America. J Pediatr 2008;153:659–62, 662.e1-3	10.1016/j.jpeds.2008.07.50	Drug utilization	Descriptive	Cohort	Data not combined	3: Disease Analyzer (France); Disease Analyzer (Germany); Disease Analyzer (UK)	2
77	Sturkenboom MCJM, Dieleman JP, Picelli G, et al. Prevalence and treatment of hypertensive patients with multiple concomitant cardiovascular risk factors in The Netherlands and Italy. J Hum Hypertens 2008;22:704–13	10.1038/jhh.2008.82	Disease epidemiology	Descriptive	Cohort	Data not combined	2: HSD (Italy); IPCI	
78	Sturkenboom MCJM, Verhamme KMC, Nicolosi A, et al. Drug use in children: Cohort in three European countries. BMJ 2008;337:a2245–a2245	10.1136/bmj.a2245	Drug utilization	Descriptive	Cohort	Pooled analysis: semi-aggregate data	3: Disease Analyzer (UK); IPCI; Pedianet	
79	van Soest EM, Valkhoff VE, Mazzaglia G, et al. Suboptimal gastroprotective coverage of NSAID use and the risk of upper gastrointestinal bleeding and ulcers: an observational study using three European databases. Gut 2011;60:1650–9	10.1136/gut.2011.239848	Drug safety	Analytical	Case-control	Pooled analysis: individual patient data	3: CPRD; HSD (Italy); IPCI	
80	van Staa TP, Sprafka JM. Study of adverse outcomes in women using testosterone therapy. Maturitas 2009;62:76–80	10.1016/j.maturitas.2008.11.001	Drug safety	Analytical	Cohort	Pooled analysis: individual patient data	2: CPRD; THIN	
81	Brankin E, Walker M, Lynch N, et al. The impact of dosing frequency on compliance and persistence with bisphosphonates among postmenopausal women in the UK: evidence from three databases. Curr Med Res Opin 2006;22:1249–56	10.1185/030079906X112688	Drug utilization	Analytical	Cohort	Data not combined	3: CPRD; DIN; Disease Analyzer (UK)	
82	Bremner SA, Carey IM, DeWilde S, et al. Early-life exposure to antibacterials and the subsequent development of hayfever in childhood in the UK: case-control studies using the General Practice Research Database and the Doctors' Independent Network. Clin Exp Allergy 2003;33:1518–25	10.1046/j.1365-2222.2003.01794.x	Disease epidemiology	Analytical	Case-control	Meta-analysis: fixed effects	2: CPRD; DIN	
83	Bremner SA, Carey IM, DeWilde S, et al. Timing of routine immunisations and subsequent hay fever risk. Arch Dis Child 2005;90:567–73	10.1136/adc.2004.051714	Disease epidemiology	Analytical	Case-control	Meta-analysis: fixed effects	2: CPRD; DIN	
84	Bremner SA, Carey IM, DeWilde S, et al. Vaccinations, infections and antibacterials in the first grass pollen season of life and risk of later hayfever. Clin Exp Allergy 2007;37:512–7	10.1111/j.1365-2222.2007.02697.x	Disease epidemiology	Analytical	Case-control	Meta-analysis: fixed effects	2: CPRD; DIN	
86	Carey IM, Cook DG, De Wilde S, et al. Implications of the problem orientated medical record (POMR) for research using electronic GP databases: a comparison of the Doctors Independent Network Database (DIN) and the General Practice Research Database (GPRD). BMC Fam Pract 2003;4:14	10.1186/1471-2296-4-14	Methodology / data quality	Descriptive	Cohort	Data not combined	2: CPRD; DIN	
88	De Wilde S, Carey IM, Bremner SA, et al. A comparison of the recording of 30 common childhood conditions in the Doctor's Independent Network and General Practice Research Databases. Heal Stat Q 2004;:21–31	pmid:15704391	Methodology / data quality	Descriptive	Cohort	Data not combined	2: CPRD; DIN	
89	Hernández-Díaz S, García Rodríguez LA. Cardioprotective aspirin users and their excess risk of upper gastrointestinal complications. BMC Med 2006;4:22	10.1186/1741-7015-4-22	Drug safety	Descriptive	Cohort	Data not combined	2: BIFAP; CPRD	
90	Jordan K, Clarke AM, Symmons DPM, et al. Measuring disease prevalence: a comparison of musculoskeletal disease using four general practice consultation databases. Br J Gen Pract 2007;57:7–14	pmid:17244418	Disease epidemiology	Descriptive	Cohort	Data not combined	2: CPRD; CiPCA	2
93	Price DB, Gefen E, Gopalan G, et al. Real-life effectiveness and safety of salbutamol Steri-NebTM vs. Ventolin Nebules® for exacerbations in patients with COPD: Historical Cohort. PLoS One 2018;13:e0191404	10.1371/journal.pone.0191404	Drug comparative effectiveness	Analytical	Cohort	Pooled analysis: individual patient data	2: CPRD; OPCRD	
94	Abbing-Karahagopian V, Huerta C, Souverein PC, et al. Antidepressant prescribing in five European countries: application of common definitions to assess the prevalence, clinical observations, and methodological implications. Eur J Clin Pharmacol 2014;70:849–57	10.1007/s00228-014-1676-z	Drug utilization	Descriptive	Cohort	Data not combined	5: AHC (Mondriaan); BIFAP; CPRD; NPCRD (Mondriaan); THIN	2

For peer review only

Study ID[a]	Citation	Resource locator: [doi unless specified otherwise][b]	Study topic	Study type	Study design[c]	Data combining	Primary care EHR data sources[d]	Other sources (if applicable)
101	Charlton RA, Jordan S, Pierini A, et al. Selective serotonin reuptake inhibitor prescribing before, during and after pregnancy: a population-based study in six European regions. BJOG 2015;122:1010–20	10.1111/1471-0528.13143	Drug utilization	Descriptive	Cohort	Data not combined	2: CPRD; SAIL	4
102	Coloma PM, de Ridder M, Bezemer I, et al. Risk of cardiac valvulopathy with use of bisphosphonates: a population-based, multi-country Case-control. Osteoporos Int 2016;27:1857–67	10.1007/s00198-015-3441-2	Drug safety	Analytical	Case-control	Multiple: pooled IPD + meta-analysis (RE + FE)	3: HSD (Italy); IPCI; THIN	3
103	Coloma PM, Schuemie MJ, Trifirò G, et al. Drug-induced acute myocardial infarction: identifying 'prime suspects' from electronic healthcare records-based surveillance system. PLoS One 2013;8:e72148	10.1371/journal.pone.0072148	Methodology / data quality	Analytical	Cohort	Pooled analysis: individual patient data	3: HSD (Italy); IPCI; Pedianet	4
104	Coloma PM, Trifirò G, Schuemie MJ, et al. Electronic healthcare databases for active drug safety surveillance: is there enough leverage?.Pharmacoepidemiol Drug Saf 2012;21:611–21	10.1002/pds.3197	Drug safety	Descriptive	Cohort	Pooled analysis: semi-aggregate data	4: HSD (Italy); IPCI; Pedianet; QResearch	4
105	de Bie S, Coloma PM, Ferrajolo C, et al. The role of electronic healthcare record databases in paediatric drug safety surveillance: a retrospective Cohort. Br J Clin Pharmacol 2015;80:304–14	10.1111/bcp.12610	Drug safety	Descriptive	Cohort	Pooled analysis: semi-aggregate data	3: HSD (Italy); IPCI; Pedianet	4
106	de Groot MCH, Klungel OH, Leufkens HGM, et al. Sources of heterogeneity in case-control studies on associations between statins, ACE-inhibitors, and proton pump inhibitors and risk of pneumonia. Eur J Epidemiol 2014;29:767–75	10.1007/s10654-014-9941-0	Drug safety	Analytical	Case-control	Data not combined	3: AHC (Mondriaan); LRGP; NPCRD (Mondriaan)	2
107	de Groot MCH, Schuerch M, de Vries F, et al. Antiepileptic drug use in seven electronic health record databases in Europe: a methodologic comparison. Epilepsia 2014;55:666–73	10.1111/epi.12557	Drug utilization	Descriptive	Cohort	Data not combined	5: AHC (Mondriaan); BIFAP; CPRD; NPCRD (Mondriaan); THIN	2
108	Ferrajolo C, Coloma PM, Verhamme KMC, et al. Signal detection of potentially drug-induced acute liver injury in children using a multi-country healthcare database network. Drug Saf 2014;37:99–108	10.1007/s40264-013-0132-9	Drug safety	Analytical	Multiple: cohort; SCCS	Pooled analysis: individual patient data	3: HSD (Italy); IPCI; Pedianet	4
110	Huerta C, Abbing-Karahagopian V, Requena G, et al. Exposure to benzodiazepines (anxiolytics, hypnotics and related drugs) in seven European electronic healthcare databases: a cross-national descriptive study from the PROTECT-EU Project. Pharmacoepidemiol Drug Saf 2016;25 Suppl 1:56–65	10.1002/pds.3825	Drug utilization	Descriptive	Cohort	Data not combined	5: AHC (Mondriaan); BIFAP; CPRD; NPCRD (Mondriaan); THIN	2
111	Korhonen P, Heintjes EM, Williams R, et al. Pioglitazone use and risk of bladder cancer in patients with type 2 diabetes: retrospective Cohort using datasets from four European countries. BMJ 2016;354:i3903	10.1136/bmj.i3903	Drug safety	Analytical	Cohort	Multiple: pooled IPD + meta-analysis (RE + FE)	2: CPRD; PHARMO GP	3
112	LoCasale R, Kern DM, Chevalier P, et al. Description of cardiovascular event rates in patients initiating chronic opioid therapy for noncancer pain in observational cohort studies in the US, UK, and Germany. Adv Ther 2014;31:708–23	10.1007/s12325-014-0131-y	Drug safety	Descriptive	Cohort	Data not combined	2: CPRD; Disease Analyzer (Germany)	1
113	Masclee GMC, Valkhoff VE, Coloma PM, et al. Risk of upper gastrointestinal bleeding from different drug combinations. Gastroenterology 2014;147:784-792.e9	10.1053/j.gastro.2014.06.7	Drug safety	Analytical	SCCS	Multiple: pooled IPD + meta-analysis (RE)	3: HSD (Italy); IPCI; Pedianet	4
114	Mokhles MM, Trifirò G, Dieleman JP, et al. The risk of new onset heart failure associated with dopamine agonist use in Parkinson's disease. Pharmacol Res 2012;65:358–64	10.1016/j.phrs.2011.11.009	Drug safety	Analytical	Case-control	Multiple: pooled IPD + meta-analysis (RE + FE)	3: HSD (Italy); IPCI; THIN	1
115	Rottenkolber M, Voogd E, van Dijk L, et al. Seasonal changes in prescribing of long-acting beta-2-agonists-containing drugs. Respir Med 2015;109:828–37	10.1016/j.rmed.2015.01.010	Drug utilization	Descriptive	Cohort	Data not combined	5: AHC (Mondriaan); BIFAP; CPRD; NPCRD	1

For peer review only - http://bmjopen.bmj.com/site/about/guidelines.xhtml

Study ID[a]	Citation	Resource locator: [doi unless specified otherwise][b]	Study topic	Study type	Study design[c]	Data combining	Primary care EHR data sources[d]	Other sources (if applicable)
							(Mondriaan); THIN	
116	Rottenkolber M, Voogd E, van Dijk L, et al. Time trends of period prevalence rates of patients with inhaled long-acting beta-2-agonists-containing prescriptions: a European comparative database study. PLoS One 2015;10:e0117628	10.1371/journal.pone.0117628	Drug utilization	Descriptive	Cohort	Data not combined	5: AHC (Mondriaan); BIFAP; CPRD; NPCRD (Mondriaan); THIN	2
117	Trifirò G, de Ridder M, Sultana J, et al. Use of azithromycin and risk of ventricular arrhythmia. CMAJ 2017;189:E560–8	10.1503/cmaj.160355	Drug safety	Analytical	Case-control	Multiple: pooled IPD + meta-analysis (RE)	3: HSD (Italy); IPCI; THIN	4
118	Tyczynski JE, Oleske DM, Klingman D, et al. Safety assessment of an anti-obesity drug (sibutramine): a retrospective Cohort. Drug Saf 2012;35:629–44	10.2165/11599220-000000000-00000	Drug safety	Analytical	Cohort	Data not combined	2: Disease Analyzer (Germany); Disease Analyzer (UK)	
119	Coloma PM, Schuemie MJ, Trifirò G, et al. Combining electronic healthcare databases in Europe to allow for large-scale drug safety monitoring: the EU-ADR Project. Pharmacoepidemiol Drug Saf 2011;20:1–11	10.1002/pds.2053	Drug safety	Analytical	Cohort	Data not combined	4: HSD (Italy); IPCI; Pedianet; QResearch	4
121	Sen EF, Verhamme KMC, Neubert A, et al. Assessment of pediatric asthma drug use in three European countries; a TEDDY study. Eur J Pediatr 2011;170:81–92	10.1007/s00431-010-1275-7	Drug utilization	Descriptive	Cohort	Data not combined	3: Disease Analyzer (UK); IPCI; Pedianet	
122	Cainzos-Achirica M, Varas-Lorenzo C, Pottegård A, et al. Methodological challenges when evaluating potential off-label prescribing of drugs using electronic health care databases: A case study of dabigatran etexilate in Europe. Pharmacoepidemiol Drug Saf 2018;27:713–23	10.1002/pds.4416	Drug utilization	Descriptive	Cross-sectional	Data not combined	2: CPRD; LPD (France)	1
124	Martín-Merino E, Petersen I, Hawley S, et al. Risk of venous thromboembolism among users of different anti-osteoporosis drugs: a population-based cohort analysis including over 200,000 participants from Spain and the UK. Osteoporos Int 2018;29:467–78	10.1007/s00198-017-4308-5	Drug safety	Analytical	Cohort	Data not combined	2: BIFAP; CPRD	
125	Anyanwagu U, Owen K, Mamza J, et al. Demographics, insulin use and clinical targets in type 2 diabetes insulin users: comparison of a local integrated diabetes service vs a UK-wide cohort. Pract Diabetes 2017;34:123–8	10.1002/pdi.2099	Health services research	Descriptive	Cross-sectional	Data not combined	2: EIDS; THIN	
127	Chiquette E, Oral EA, Garg A, et al. Estimating the prevalence of generalized and partial lipodystrophy: findings and challenges. Diabetes Metab Syndr Obes 2017;10:375–83	10.2147/DMSO.S130810	Disease epidemiology	Descriptive	Cross-sectional	Data not combined	2: CPRD; Humedica	2
128	Lo Re V, Carbonari DM, Saine ME, et al. Postauthorization safety study of the DPP-4 inhibitor saxagliptin: a large-scale multinational family of cohort studies of five outcomes. BMJ open diabetes Res care 2017;5:e000400	10.1136/bmjdrc-2017-000400	Drug safety	Analytical	Cohort	Meta-analysis: method not specified	2: CPRD; THIN	2
129	Price D, Thomas V, von Ziegenweidt J, et al. Switching patients from other inhaled corticosteroid devices to the Easyhaler(®): historical, matched-Cohort of real-life asthma patients. J Asthma Allergy 2014;7:31–51	10.2147/JAA.S59386	Drug comparative effectiveness	Analytical	Cohort	Pooled analysis: individual patient data	2: CPRD; OPCRD	
134	Coloma PM, Valkhoff VE, Mazzaglia G, et al. Identification of acute myocardial infarction from electronic healthcare records using different disease coding systems: a validation study in three European countries. BMJ Open 2013;3:e002862	10.1136/bmjopen-2013-002862	Drug safety	Analytical	Cohort	Pooled analysis: individual patient data	2: HSD (Italy); IPCI	1
135	Mor A, Frøslev T, Thomsen RW, et al. Antibiotic use varies substantially among adults: a cross-national study from five European Countries in the ARITMO project. Infection 2015;43:453–72	10.1007/s15010-015-0768-8	Drug utilization	Descriptive	Cohort	Data not combined	2: HSD (Italy); THIN	4
136	Poluzzi E, Diemberger I, De Ridder M, et al. Use of antihistamines and risk of ventricular tachyarrhythmia: a nested Case-control in five European countries from the ARITMO project. Eur J Clin Pharmacol 2017;73:1499–510	10.1007/s00228-017-2317-0	Drug safety	Analytical	Case-control	Multiple: pooled IPD + meta-analysis (RE)	3: HSD (Italy); IPCI; THIN	4
137	Roberto G, Leal I, Sattar N, et al. Identifying Cases of Type 2 Diabetes in Heterogeneous Data Sources: Strategy from the EMIF Project. PLoS One 2016;11:e0160648	10.1371/journal.pone.0160648	Methodology / data quality	Other	Cross-sectional	Data not combined	3: HSD (Italy); IPCI; THIN	5

For peer review only

Study ID[a]	Citation	Resource locator: [doi unless specified otherwise][b]	Study topic	Study type	Study design[c]	Data combining	Primary care EHR data sources[d]	Other sources (if applicable)
138	La Gamba F, Corrao G, Romio S, et al. Combining evidence from multiple electronic health care databases: performances of one-stage and two-stage meta-analysis in matched case-control studies. Pharmacoepidemiol Drug Saf 2017;26:1213–9	10.1002/pds.4280	Drug safety	Analytical	Case-control	Multiple: pooled IPD + meta-analysis (FE)	2: IPCI; THIN	1

[a] Record identifier used in reference management, and different from reference number used in main study text.
[b] If digital object identifier [doi] was not available, Pubmed ID [pmid] was used
[c] SCCS – self controlled case series; CCX – case crossover:
[d] See Table S2 for further details of primary care EHR data sources

For peer review only

Table S2: Summary details of primary care electronic health record (EHR) data sources used in studies included in systematic review.

Data Source ID	Short name	Source Name	Country	Clinical Coding	Drug Coding	Source reference	Resource locator[a]
1	CPRD	Clinical Practice Research Datalink	UK	Read V2	DM+D	https://www.ncbi.nlm.nih.gov/pubmed/26050254	doi: 10.1093/ije/dyv098
2	THIN	The Health Information Network	UK	Read V2	DM+D	https://www.ncbi.nlm.nih.gov/pubmed/22828580	doi: 10.14236/jhi.v19i4.820
3	QResearch	QResearch	UK	Read V2 / SNOMED-CT	DM+D	https://bmjopen.bmj.com/content/5/9/e008503.long	doi:10.1136/bmjopen-2015-008503
4	IPCI	Integrated Primary Care Information database	Netherlands	ICPC	ATC	https://www.ncbi.nlm.nih.gov/pubmed/10805025	doi:10.1055/s-0038-1634402
5	Pedianet	PediaNet	Italy		ATC	https://www.ncbi.nlm.nih.gov/pubmed/15930187	doi:10.1542/peds.2004-0040
7	SAIL	SAIL Databank	UK	Mixed	DM+D	http://www.ncbi.nlm.nih.gov/pubmed/19732426	doi:10.1186/1472-6963-9-157
8	LPD (France)	Cegedim LPD (Longitudinal Patient Data): France	France	ICD-9/10 CM	ATC	https://epidemiologie-france.aviesan.fr/en/ccontent/pdf/(ObjectId)/91221	url:https://epidemiologie-france.aviesan.fr/en/ccontent/pdf/(ObjectId)/91221
9	NPCRD (Mondriaan)	Netherlands Primary Care Research Database (NPCRD)	Netherlands	ICPC/ICD	ATC	http://www.ncbi.nlm.nih.gov/pubmed/25154551	doi:10.1016/s1098-3015(10)74933-0
11	BIFAP	Spanish Base de datos para la Investigación Farmacoepidemiológica en Atención Primaria (BIFAP)	Spain	ICPC	ATC	https://www.ncbi.nlm.nih.gov/pubmed/25066450	doi:10.1007/s00228-012-1386-3
12	HSD (Italy)	Health Search Database (Italy)	Italy	ICD-9 CM	ATC	https://www.ncbi.nlm.nih.gov/pubmed/24816637	doi:10.1371/journal.pone.0095419
15	AHC (Mondriaan)	Mondriaan Almere Healthcare group (AHC) database	Netherlands	ICPC/ICD	ATC	https://www.valueinhealthjournal.com/article/S1098-3015(10)74933-0/abstract	doi:10.1016/s1098-3015(10)74933-0
17	SIDIAP	Sistema de Información para el desarrollo de la Investigación en Atención Primaria	Spain	ICD-10	ATC	http://www.sidiap.org	url:http://www.sidiap.org
25	CDARS	Hong Kong Clinical Data Analysis and Reporting System database	Hong Kong	ICD-9 CM		https://www.ncbi.nlm.nih.gov/pubmed/24833754	doi:10.1093/jac/dku145
26	ResearchOne	ResearchOne	UK	CTV3	DM+D	https://www.ncbi.nlm.nih.gov/pubmed/26944937	url:http://www.researchone.org/
28	Julius (Mondriaan)	Julius Primary Care Network Database	Netherlands		ATC	https://www.ncbi.nlm.nih.gov/pubmed/30253760	doi:10.1016/s1098-3015(10)74933-0
29	NWEH-LDB	NorthWest EHealth linked database	UK	Read V2/SNOMED-CT	DM+D	https://bmcmedinformdecismak.biomedcentral.com/articles/10.1186/s12911-015-0132-z	doi:10.1186/s12911-015-0132-z
30	OCHIN Clarity (Epic EHR)	Oregon Community Health Information Network (OCHIN)	USA	ICD-9 CM	ATC	https://www.ncbi.nlm.nih.gov/pubmed/26934626	doi:10.7812/TPP/15-081
31	KP Clarity (Epic EHR)	Kaiser Permanente Epic EHR Clarity database	USA	ICD-9 CM	ATC	https://www.ncbi.nlm.nih.gov/pubmed/26934626	doi:10.7812/TPP/15-081
32	OPCRD	Optimum Patient Care Research Database (OPCRD)	UK	Read V2	DM+D	https://optimumpatientcare.org/database-overview/	url:https://optimumpatientcare.org/database-overview/
33	LPD (Spain)	IMS Longitudinal Patient Database (Spain)	Spain	ICD-9/10 CM	ATC	https://www.ncbi.nlm.nih.gov/pubmed/27155295	doi:10.1111/ejh.12776
34	LPD (Germany)	IMS Longitudinal Patient Database (Germany)	Germany	ICD-9/10 CM	ATC	https://www.ncbi.nlm.nih.gov/pubmed/27155295	doi:10.1111/ejh.12776
35	LPD (Belgium)	IMS Longitudinal Patient Database (Belgium)	Belgium	ICD-9/10 CM	ATC	https://www.ncbi.nlm.nih.gov/pubmed/27155295	doi:10.1111/ejh.12776
39	SIR	Salford Integrated Record (SIR)	UK	Read V2	DM+D	https://www.ncbi.nlm.nih.gov/pubmed/25481707	doi:10.1007/s00125-014-3456-9
41	Disease Analyzer (Germany)	IMS Disease Analyzer (Germany)	Germany	ICD-10	ATC	https://www.ncbi.nlm.nih.gov/pubmed/19825325	doi:10.5414/cpp47617
42	Disease Analyzer (France)	IMS Disease Analyzer (France)	France	ICD-10	ATC	https://www.ncbi.nlm.nih.gov/pubmed/19825325	doi:10.5414/cpp47617

For peer review only

Data Source ID	Short name	Source Name	Country	Clinical Coding	Drug Coding	Source reference	Resource locator[a]
46	Disease Analyzer (UK)	IMS Disease Analyzer (UK)	UK	ICD-10	ATC	https://bpspubs.onlinelibrary.wiley.com/doi/full/10.1111/j.1365-2125.2005.02450.x	doi:10.1111/j.1365-2125.2005.02450.x
51	DIN	Doctors' Independent Network (DIN)	UK	Read V2	BNF	https://www.ncbi.nlm.nih.gov/pubmed/15171985	doi:10.1016/j.ijmedinf.2004.02.002
52	Intego	Intego Network	Belgium	ICPC	ATC	http://www.ncbi.nlm.nih.gov/pubmed/24906941	doi:10.1186/1472-6947-14-48
53	Jonkoping County	Jonkoping County Primary Care Database	Sweden	ICD-10	ATC	https://www.ncbi.nlm.nih.gov/pubmed/28277045	doi:10.1080/02813432.2017.1288680
58	Resoprim	Resoprim Project	Belgium	ICPC	ATC	https://www.ncbi.nlm.nih.gov/pubmed/18953130	doi:10.3233/978-1-58603-922-6-103
59	Mediplus	IMS Mediplus	UK	Read V2	BNF	https://www.ncbi.nlm.nih.gov/pubmed/16368704	doi:10.1093/fampra/cmi106
62	CiPCA	Consultations in Primary Care Archive	UK	Read V2	BNF	https://www.keele.ac.uk/mrr/cipcadatabase/	url:https://www.keele.ac.uk/mrr/cipcadatabase/
65	THALES	THALES Database	France	ICD-9 CM	ATC	http://www.ncbi.nlm.nih.gov/pubmed/11111209	doi:10.1159/000052402
72	LRGP	Leidsche Rijn GP database (Mondriaan)	Netherlands	ICPC	ATC	https://www.ncbi.nlm.nih.gov/pubmed/15921047	doi:10.1007/s10654-004-5689-2
74	PHARMO GP	PHARMO GP database	Netherlands	ICPC	ATC	https://www.ncbi.nlm.nih.gov/pubmed/27530399	doi:10.1136/bmj.i3903
76	EIDS	Erewash (Integrated) Diabetes Service	UK	Read V2	DM+D	https://onlinelibrary.wiley.com/doi/full/10.1002/pdi.2099	doi:10.1002/pdi.2099
79	Humedica	Humedica NorthStar from Optum	USA	ICD-9 CM	ATC	https://www.nihcollaboratory.org/Pages/OptumInsight-MetaData-Table.aspx	doi:10.2147/DMSO.S130810
80	GE Healthcare	GE Healthcare Database	USA	ICD-9 CM	ATC	https://www.ncbi.nlm.nih.gov/pubmed/29066925	doi:10.2147/DMSO.S130810

[a] doi: Digital object identifier; url: Uniform resource locator

For peer review only

Multidatabase Systematic Review: Supplementary Appendix

Appendix S1: Review Protocol: Approaches for combining primary care EHR data from multiple sources: a systematic review of observational studies

For peer review only

Appendix S1: Review Protocol: Approaches for combining primary care EHR data from multiple sources: a systematic review of observational studies

Contents

1 Introduction 2
2 Rationale and scope 2
2.1 Objectives 2
3 Methods 2
3.1 Eligibility Criteria 2
3.2 Information sources 3
3.3 Search strategy 3
3.3.1 Test sets 3
3.3.2 Conceptual searches 3
3.3.3 Search of named databases and common terms 4
3.4 Study records 4
3.4.1 Data Management 4
3.4.2 Selection process 4
3.4.3 Data extraction 5
4 Data synthesis 6
5 References 7
6 Appendices 8
6.1 Appendix I: OVID Medline Search Strategy for Systematic Review 8
6.2 Appendix 2: Data extraction tables 10

For peer review only

For peer review only - http://bmjopen.bmj.com/site/about/guidelines.xhtml

1 Introduction

A recent systematic review evaluated methods for data management and analysis of multi-database studies in pharmacoepidemiology. The current study adopted a similar approach, but with a specific focus on studies combining primary care EHR databases, while expanding the scope to include all areas of observational epidemiology and healthcare database research.

2 Rationale and scope

The primary aim was to describe the full range of completed studies which brought together primary care electronic health record (EHR) data from two or more sources, and to generate a clear overview of methods used to manage, assess variability in, and analyse the data. The main motivation for the review was to inform a planned study of cancer risk in patients with Huntington's disease combining data from two UK primary care EHR databases.

The review specifically covered 'horizontal' combination of data from different sources, containing data from different sets of individuals (possibly after deduplication). Here the primary purpose of combining might be to increase the number of individuals available for analysis, or increase the range of population settings to which findings can be applied (i.e. increase external validity). The review did not include studies where data sources were only combined 'vertically' i.e. linkage studies whereby data on the same individuals was combined to provide richer (deeper) information about each study participant.

As the focus was on analysis of primary care EHR data, the review was restricted to studies using primary care EHR data from at least two sources. For the purpose of this review, primary care EHR data was defined as data collected by primary care clinicians and related staff for the purpose of diagnosis, treatment, management, and delivery of care of individual patients. It may include information collected or contributed by other care providers (1). It excludes data generated primarily for administrative purposes such as claims data.

The review was conducted following the PRISMA guidelines for reporting in systematic reviews (2,3) [http://www.prisma-statement.org].

2.1 Objectives

1. Identify studies which combined data from two or more sources of primary care EHR data.
2. Summarise key study characteristics, including the main reasons or motivations for combining data from different EHR databases
3. Describe the methods used to manage and analyse data including, where applicable, methods for combining data.
4. Describe the methods used to assess and report heterogeneity between primary care EHR data sources.
5. Describe and summarise any reported differences between different primary care EHR data sources.

Quality and completeness of reporting of methods was assessed using criteria adapted from the STROBE (4) and RECORD (5) guidelines. No formal assessment of quality in terms of risk of bias was attempted, either for individual studies, or for particular methodological approaches.

3 Methods

3.1 Eligibility Criteria

1. Peer reviewed, English language publication of an observational study.

For peer review only - http://bmjopen.bmj.com/site/about/guidelines.xhtml

2. Study participants selected from at least *two* different primary care EHR data sources.
3. Re-analyses of previously reported cohorts were included if they used substantially different methods.

There were no specific eligibility criteria relating to exposures, comparator groups, outcomes, or study design.

3.2 Information sources

The following databases were searched for eligible studies

1. Medline (OVID)
2. EMBASE (OVID)

3.3 Search strategy

The key challenges anticipated when searching for relevant studies were the lack of a specific MeSH concept for multi-database studies (6), and the lack of consensus on terminology for such studies in the published literature. This raised the possibility of having to hand search all database studies.

3.3.1 Test sets

Given the challenges outlined above, the performance of different search strategies was evaluated for their ability to recall results from the following test references sets:

Test Set 1: 1673 publications using CPRD or GPRD databases were identified using keyword searches in Medline.

Test Set 2: an *ad hoc* sample of 14 records identified from a published systematic review of multi-database pharmacoepidemiology studies (6), plus a small number identified from non-systematic review of the literature. All of these studies used at least one primary care EHR data source.

3.3.2 Conceptual searches

An initial search strategy was defined based on 3 concepts, identified using both MeSH terms and keywords, and the sensitivity of each concept was assessed against Test Sets 1 and 2 :

1. Database studies: this included MeSH terms and keyword searches for databases and related concepts such as Electronic Health Records, and Computerized Medical Records System. This concept was reasonably sensitive for recalling CPRD/GPRD studies (Test Set 1 sensitivity= 1365/1673 = 0.82), and all records in the sample of multi-database studies (Test Set 2 sensitivity 14/14 = 1). However it returned over 395 thousand results.
2. Primary care setting: a combined MeSH term and keyword search returned over 280 thousand records, but had very low sensitivity with both Test Set 1 (526/1673 = 0.31), and only moderate sensitivity with Test Set 2 (10/14 = 0.71).
3. (Observational) epidemiology studies: The InterTASC Information Specialists' Sub-Group Search Filter Resource (7) was accessed to identify potentially suitable and validated search filters. Waffenschmidt *et al* (8) reviewed search strategies to identify epidemiological studies, concluding that there was "no suitable approach to conducting *efficient* systematic searches for epidemiologic publications in bibliographic databases". One filter, from a systematic review of Hepatitis C prevalence in prisons by Larney *et al* (9), was found to be suitably sensitive, recalling almost 96% of their test set of 729 references. This filter had very high sensitivity for CPRD/GPRD studies in Test Set 1 (1593/1673 = 0.95), and also recalled all records in Test Set 2 (sensitivity 14/14 = 1). However it returned over 6 million records.

For peer review only - http://bmjopen.bmj.com/site/about/guidelines.xhtml

A Medline search combining all 3 of the concepts above returned 14309 records and recalled only 10 of the 14 records in Test Set 2 (sensitivity 0.71), with the sensitivity being limited by the 'Primary Care' concept. However any attempt to broaden this concept to improve sensitivity would have increased the number of recalled records beyond what could be feasibly reviewed manually. For example, broadening the Primary Care concept to include 'population-based' studies, allowed recall of 12/14 records from Test Set 2, but increased the total number of records retrieved to 23656.

3.3.3 Search of named databases and common terms

Given the relatively poor performance of the conceptual searches, an alternative strategy was developed using a combination of named databases, and commonly used key words and phrases. Three sources were used to compile a list of candidate primary care EHR (or closely related) databases:

1. ENCePP Resources Database: the European Network of Centres for Pharmacoepidemiology and Pharmacovigilance (ENCePP) maintains a searchable database of research organisations, networks and data sources (10). Despite a strong European focus, the register is not restricted to European data sources. The database was searched to identify 20 registered data sources identified as a 'Routine primary care electronic patient registry'.
2. B.R.I.D.G.E. TO DATA®: a "non-profit online reference describing population healthcare databases for use in epidemiology and health outcomes research" (10). A search interface is provided as a subscription service, however a simple listing of 286 named database resources was downloaded (23 Jan 2018) and searched for candidate primary care databases.
3. A list of 41 databases or primary care research networks identified in a 2017 review by Gentil et al (11) which examined factors associated with successful implementation of initiatives to collect and curate collections of primary care electronic health record data.

Abstracts of published studies identified from the initial named database search were scanned for additional terms and phrases used to describe the primary care EHR data sources, and these were added to the final search. Finally, reference lists of papers selected for full review were searched for additional studies.

The full search strategy used to search the Medline database is included in Appendix I.

This search was able to recall 12/14 records in Test Set 2 (sensitivity = 0.86), and all records in Test Set 1 (by definition - since CPRD/GPRD were among the named databases included in the search).

3.4 Study records

3.4.1 Data Management

All search results were exported from OVID in batches, with copies of export files retained. The references were imported into Mendeley V1.1 (Mendeley, Elsevier, Amsterdam, NL). Details of studies selected for full review were exported into a Microsoft Access database, in which was used to record subsequent inclusion/exclusion decisions and data extraction.

3.4.2 Selection process

Initial screening of all selected titles and abstracts was undertaken by 1 reviewer (DD). A second reviewer (MC) screened a 20% random sample of all abstracts. Full text was reviewed in instances where it was not possible to assess eligibility from the title and abstract alone.

Full text was then obtained for all papers selected during the initial abstract screening, and read by two reviewers, who completed the eligibility assessment before and performing data extraction.

For peer review only - http://bmjopen.bmj.com/site/about/guidelines.xhtml

3.4.3 Data extraction

Each reviewer extracted standardised information via a data collection form into a review database (MS Access). The following information was collected:

Year of publication
Primary care EHR data source details:<br>• number of sources<br>• name(s) of database<br>• country
Other (non-primary care EHR) data source details:<br>• number of sources<br>• name(s) of database<br>• type of database e.g. claims, disease registry<br>• country
Study type or broad objective e.g.:<br>• Descriptive e.g. drug utilisation, disease epidemiology<br>• Comparative or hypothesis testing e.g. comparative treatment effectiveness, drug safety, disease epidemiology<br>• Disease risk prediction<br>• Methodology / data quality assessment<br>• Health service research
Study design e.g.:<br>• Cross sectional<br>• Case-control<br>• Cohort<br>• Case-only designs<br>• Time series
Target population(s) for study:
Main exposure(s) if applicable e.g.:<br>• Drug treatment<br>• Disease risk factor<br>• Other
Main outcome(s) if applicable e.g.:<br>• All cause mortality<br>• Disease<br>• Treatment patterns<br>• Other
Main analysis methods, including confounder control e.g.:<br>• descriptive using summary statistics<br>• incidence or prevalence calculations<br>• multiple regression modelling
Motivation or rationale for using and/or combining data sources e.g.:<br>• increase study power<br>• assess consistency of findings in multiple settings<br>• international comparisons
Assssement of heterogeneity of exposures, outcomes and effect estimates e.g.:<br>• descriptive only (no formal comparisons)<br>• univariate comparisons<br>• formal tests for heterogeneity (Q-test, I-test)
Main approach for combining data sources e.g.:<br>• Data not combined: results presented separately for each source<br>• Meta-analysis of aggregate results from each data source

For peer review only - http://bmjopen.bmj.com/site/about/guidelines.xhtml

• Meta-analysis of semi-aggregated results from each data source<br>• Pooled analysis of individual patient data
Data management and analysis e.g.:<br>• Data managed and analysed separately by each database partner<br>• Use of common protocol<br>• Use of common data model (study specific, or externally defined e.g. OMOP CDM)<br>• Use of common analysis programs<br>• Data management and analysis arrangements (distributed, central, hybrid)<br>• Data sharing model (individual, semi-aggregate, aggregate)

4 Data synthesis

Results will be summarised in tables which will describe .

- basic characteristics of included studies: study design, statistical method
- rationale for combining databases
- methods used to assess heterogeneity
- methods use for combining or synthesising results

Further narrative descriptions will focus on specific subgroups. For example analytical studies which combined two or more databases from the same country.

For peer review only

For peer review only - http://bmjopen.bmj.com/site/about/guidelines.xhtml

5 References

1. Häyrinen K, Saranto K, Nykänen P. Definition, structure, content, use and impacts of electronic health records: a review of the research literature. Int J Med Inform [Internet]. 2008 May;77(5):291–304. Available from: http://www.ncbi.nlm.nih.gov/pubmed/17951106

2. Liberati A, Altman DG, Tetzlaff J, Mulrow C, Gøtzsche PC, Ioannidis JPA, et al. The PRISMA Statement for Reporting Systematic Reviews and Meta-Analyses of Studies That Evaluate Health Care Interventions: Explanation and Elaboration. PLoS Med [Internet]. 2009 Jul 21;6(7):e1000100. Available from: http://dx.plos.org/10.1371/journal.pmed.1000100

3. Moher D, Liberati A, Tetzlaff J, Altman DG, PRISMA Group. Preferred reporting items for systematic reviews and meta-analyses: the PRISMA statement. Ann Intern Med [Internet]. 2009 Aug 18;151(4):264–9, W64. Available from: http://www.ncbi.nlm.nih.gov/pubmed/19622511

4. von Elm E, Altman DG, Egger M, Pocock SJ, Gøtzsche PC, Vandenbroucke JP, et al. The Strengthening the Reporting of Observational Studies in Epidemiology (STROBE) statement: guidelines for reporting observational studies. J Clin Epidemiol [Internet]. 2008 Apr;61(4):344–9. Available from: http://www.ncbi.nlm.nih.gov/pubmed/18313558

5. Benchimol EI, Smeeth L, Guttmann A, Harron K, Moher D, Petersen I, et al. The REporting of studies Conducted using Observational Routinely-collected health Data (RECORD) statement. PLoS Med [Internet]. 2015 Oct;12(10):e1001885. Available from: http://www.ncbi.nlm.nih.gov/pubmed/26440803

6. Bazelier MT, Eriksson I, de Vries F, Schmidt MK, Raitanen J, Haukka J, et al. Data management and data analysis techniques in pharmacoepidemiological studies using a pre-planned multi-database approach: a systematic literature review. Pharmacoepidemiol Drug Saf [Internet]. 2015 Sep [cited 2017 Feb 15];24(9):897–905. Available from: http://doi.wiley.com/10.1002/pds.3828

7. Glanville J, Lefebvre C, Wright K. ISSG Search Filter Resource [Internet] [Internet]. York (UK): The InterTASC Information Specialists' Sub-Group; 2008. 2008 [cited 2018 Mar 12]. Available from: https://sites.google.com/a/york.ac.uk/issg-search-filters-resource/how-to-cite-this-site

8. Waffenschmidt S, Hermanns T, Gerber-Grote A, Mostardt S. No suitable precise or optimized epidemiologic search filters were available for bibliographic databases. J Clin Epidemiol [Internet]. 2017 Feb 1 [cited 2017 Oct 17];82:112–8. Available from: http://www.sciencedirect.com/science/article/pii/S0895435616303663

9. Larney S, Kopinski H, Beckwith CG, Zaller ND, Jarlais D Des, Hagan H, et al. Incidence and prevalence of hepatitis C in prisons and other closed settings: Results of a systematic review and meta-analysis. Hepatology [Internet]. 2013 Oct [cited 2017 Oct 17];58(4):1215–24. Available from: http://www.ncbi.nlm.nih.gov/pubmed/23504650

10. ENCePP. ENCePP Resource Database [Internet]. [cited 2018 Jan 18]. Available from: http://www.encepp.eu/encepp/resourcesDatabase.jsp

11. Gentil M-L, Cuggia M, Fiquet L, Hagenbourger C, Le Berre T, Banâtre A, et al. Factors influencing the development of primary care data collection projects from electronic health records: a systematic review of the literature. BMC Med Inform Decis Mak [Internet]. 2017 Dec 25 [cited 2017 Oct 24];17(1):139. Available from: http://bmcmedinformdecismak.biomedcentral.com/articles/10.1186/s12911-017-0538-x

For peer review only - http://bmjopen.bmj.com/site/about/guidelines.xhtml

6 Appendices

6.1 Appendix I: OVID Medline Search Strategy for Systematic Review

Run on 16 Feb 2018 on Medline to Feb Week 2 2018

Item	Term
1	Lifelink.mp.
2	Disease Analyzer.mp.
3	(OsMed not dysplasia).mp.
4	EpiChron.mp.
5	(Integrated Primary Care Information or IPCI or Interdisciplinary Processing of Clinical Information).mp.
6	PHARMO.mp.
7	(Primary Care Clinical Informatics Unit or PCCIU).mp.
8	(BIFAP or Database for Pharmacoepidemiolog* Research in Primary Care or Base de datos para la Investigacion Farmacoepidemiologica en Atencion Primaria).mp.
9	(SIDIAP or Information System for the Development of Research in Primary Care or (Sistema and Desenvolupament and Atencio Primaria)).mp.
10	((LINH and database) or Netherlands Information Network of General Practice or Landelijk Informatie Netwerk Huisatsenzorg).mp.
11	(NIVEL adj3 database).mp.
12	(CPRD or Clinical Practice Research Data*).mp.
13	(GPRD or General Practice Research Data*).mp.
14	(OPCRD or Optimum Patient Care Research Data*).mp.
15	((THIN adj4 database) or Health Information Network or Health Improvement Network).mp.
16	(QResearch or Q Research).mp.
17	(ResearchOne or (Research One adj4 database*)).mp.
18	(DIN LINK or (DIN adj4 database*) or Doctors Independent Network).mp.
19	((SAIL adj4 Data*) or Secure Anon* Information Link*).mp.
20	(Arianna data* or (Caserta and database)).mp.
21	Pedianet.mp.
22	(Health Search and (Database or Dataset)).mp.
23	Longitudinal Patient Database.mp.
24	(mediplus and database).mp.
25	(centricity and (database* or EMR or electronic medical record*)).mp.
26	OCHIN.mp.
27	PHINEX.mp.
28	Regenstrief Medical Record.mp.
29	(Clalit and database).mp.
30	(Electronic Medical Record Administrative data Linked Database or EMRALD).mp.
31	(Intego or (database* and (general practice or primary care) and Belgi*)).mp.
32	Julius General Practi*.mp.
33	((primary care or primary health care or general practi* or family practi* or ambulatory care) adj4 database*).mp.
34	population database*.mp
35	(healthcare adj2 database*).mp
36	health care database*.mp
37	(electronic health* adj2 database*).mp

For peer review only - http://bmjopen.bmj.com/site/about/guidelines.xhtml

38	(population health* adj2 database*).mp
39	((EHR or electronic health record*) adj2 database*).mp
40	Or/1-39
41	limit 40 to (abstracts and english language and yr="2000 -Current")

For peer review only

For peer review only - http://bmjopen.bmj.com/site/about/guidelines.xhtml

6.2 Appendix 2: Data extraction tables

Table name: StudyInfo1

Description: Basic publication details

Completed for: All studies selected at initial screening round

Name	Type	Size	Description
StudyID	Long Integer	4	UNIQID for study
Authors	Long Text	-	
Title	Long Text	-	
JName	Short Text	255	Name of journal
JVol	Short Text	255	Journal volume
JPage	Short Text	255	Journal pages
YearPub	Long Integer	4	Year of publication

Table name: DataSource1

Description: Key information about each primary care EHR database

Completed for: Each primary care EHR database, plus partial details collected for other databases described in the included studies

Name	Type	Size	Description
DataSourceID	Long Integer	4	UNIQID for datasource
SourceName	Short Text	255	Full or official name of database
Shortname	Short Text	255	Short name for database
Aliases	Short Text	255	Other names used in published papers
IsEHR	Short Text	10	Is it a primary care EHR database
SourceType	Integer	2	What type of database (primary care EHR or some other type)
SourceCountry	Short Text	255	Country of database
ClinicalCoding	Short Text	255	Name of clinical coding scheme (if known)
DrugCoding	Short Text	255	Name of drug coding scheme (if known)
SourceInfo1	Long Text	-	Other relevant information about data source
SourceReference	Long Text	-	Key reference for data source

Table name: Review1

Description: Summary of review process, including whether publication was selected for full review

Completed for: All studies selected at initial screening round

Name	Type	Size	Description
Review1ID	Long Integer	4	record identifier
StudyID	Long Integer	4	ID number of paper being reviewed
ReviewerID	Integer	2	Reviewer: DD or MC (or adjudicated)
IncExc	Integer	2	Inclusion / exclusion with reasons
IncExcComment 1	Long Text	-	Comment on decision to include or exclude
Review1Date	Date With Time	8	Date of completion of review

For peer review only - http://bmjopen.bmj.com/site/about/guidelines.xhtml

Table name: Review2

Description: Full details of study objectives, methods and relevant results

Completed for: All studies included after full paper review

Name	Type	Size	Description
Review2ID	Long Integer	4	record identifier
StudyID	Integer	2	Study ID (FK)
ReviewerID	Short Text	20	Reviewer: DD or MC (or Adjudicated)
Review2Status	Short Text	255	set to "in progress" once data entry is started; user specifies when completed
Review2Date	Date With Time	8	autoset when status is set to completed
Objective	Short Text	50	Short description of study objectives
OBjectiveText	Long Text	-	Further details of study objectives, including quoted text from publication if relevant
TargetPop	Short Text	255	Short description of the target population or patient group for the study
TargetPopText	Long Text	-	Further details of target population, including quoted text from publication if relevant
MainExposures	Short Text	255	Lookup: category for main exposure(s)
MainExposuresText	Long Text	-	Further details of main exposure(s), including quoted text from publication if relevant
MainOutcomes	Short Text	255	Lookup: category for main outcome(s)
MainOutcomesText	Long Text	-	Further details of main outcome(s), including quoted text from publication if relevant
StudyType	Short Text	255	Lookup: type of study e.g drug safety; disease epidemiology;
StudyTypeText	Long Text	-	Further details of study type, including quoted text from publication if relevant
StudyDesign	Short Text	255	Lookup: study design e.g. cohort, case control, etc
StudyDesignText	Long Text	-	Further details of study design, including quoted text from publication if relevant
MainRationale	Short Text	255	Lookup: main reason for combining data from multiple sources
MainRationaleText	Long Text	-	Further details of study rationale, including quoted text from publication if relevant
Stats1	Short Text	255	Lookup: main statistical method or model used
Stats1Text	Long Text	-	Further details of statistical method or model including quoted text from publication if relevant
ExposureTime	Short Text	255	Lookup: main method for modelling exposure
ConfounderControl	Short Text	255	Lookup: main method for confounder adjustment
HeterogeneityAssess	Short Text	255	Lookup: main method for assessing heterogeneity
CombineMethod	Short Text	255	Lookup: main method for combining results
CombineMethodText	Long Text	-	Further details of combination methods including quoted text from publication if relevant

For peer review only - http://bmjopen.bmj.com/site/about/guidelines.xhtml

CompareExposure	Short Text	255	Lookup: main method for comparing exposure variables in each data source
CompareOutcome	Short Text	255	Lookup: main method for comparing outcome variables in each data source
CompareOther	Short Text	255	Lookup: main method for comparing other variables in each data source
CompareText	Long Text	-	Further details of methods used to compare variables including quoted text from publication if relevant
DataManagement	Short Text	255	Lookup: how was data managed e.g. central vs multicentre etc
DataManageText	Long Text	-	Further details of data management approach including quoted text from publication if relevant
Programming	Short Text	255	Lookup: how was programming managed e.g. central vs multicentre etc
ProgrammingText	Long Text	-	Further details of programming approach including quoted text from publication if relevant

For peer review only

For peer review only - http://bmjopen.bmj.com/site/about/guidelines.xhtml

Reporting checklist for meta-analysis of observational studies.

Based on the MOOSE guidelines.

Instructions to authors

Complete this checklist by entering the page numbers from your manuscript where readers will find each of the items listed below.

Your article may not currently address all the items on the checklist. Please modify your text to include the missing information. If you are certain that an item does not apply, please write "n/a" and provide a short explanation.

Upload your completed checklist as an extra file when you submit to a journal.

In your methods section, say that you used the MOOSEreporting guidelines, and cite them as:

Stroup DF, Berlin JA, Morton SC, Olkin I, Williamson GD, Rennie D, Moher D, Becker BJ, Sipe TA, Thacker SB. Meta-analysis of observational studies in epidemiology: a proposal for reporting. Meta-analysis Of Observational Studies in Epidemiology (MOOSE) group. JAMA. 2000; 283(15):2008-2012.

		Reporting Item	Page Number
**Title**			
	#1	Identify the study as a meta-analysis of observational research	1
**Abstract**			

For peer review only

	#2	Provide a structured summary including, as applicable: background; objectives; data sources; study eligibility criteria, participants, and interventions; study appraisal and synthesis methods; results; limitations; conclusions and implications of key findings; systematic review registration number (From PRISMA checklist)	2
**Background**			
	#3a	Problem definition	4
	#3b	Hypothesis statement	n/a
	#3c	Description of study outcomes	4
	#3d	Type of exposure or intervention used	4
	#3e	Type of study designs used	4-5
	#3f	Study population	n/a
**Methods**			
Search strategy	#4a	Qualifications of searchers (eg, librarians and investigators)	23
Search strategy	#4b	Search strategy, including time period included in the synthesis and keywords	4
Search strategy	#4c	Effort to include all available studies, including contact with authors	5
Search strategy	#4d	Databases and registries searched	5

For peer review only

Search strategy	#4e	Search software used, name and version, including special features used (eg, explosion)	5
Search strategy	#4f	Use of hand searching (eg, reference lists of obtained articles)	5
Search strategy	#4g	List of citations located and those excluded, including justification	Figure 1
Search strategy	#4h	Method of addressing articles published in languages other than English	n/a
Search strategy	#4i	Method of handling abstracts and unpublished studies	n/a
Search strategy	#4j	Description of any contact with authors	5
	#5a	Description of relevance or appropriateness of studies gathered for assessing the hypothesis to be tested	n/a
	#5b	Rationale for the selection and coding of data (eg, sound clinical principles or convenience)	n/a
	#5c	Documentation of how data were classified and coded (eg, multiple raters, blinding, and interrater reliability)	5
	#5d	Assessment of confounding (eg, comparability of cases and controls in studies where appropriate)	n/a
	#5e	Assessment of study quality, including blinding of quality assessors; stratification or regression on possible predictors of study results	n/a

For peer review only

	#5f	Assessment of heterogeneity	n/a
	#5g	Description of statistical methods (eg, complete description of fixed or random effects models, justification of whether the chosen models account for predictors of study results, dose-response models, or cumulative meta-analysis) in sufficient detail to be replicated	n/a
	#5h	Provision of appropriate tables and graphics	n/a
**Results**			
	#6a	Graphic summarizing individual study estimates and overall estimate	Figure 2
	#6b	Table giving descriptive information for each study included	7-8,10
	#6c	Results of sensitivity testing (eg, subgroup analysis)	n/a
	#6d	Indication of statistical uncertainty of findings	n/a
**Discussion**			
	#7a	Quantitative assessment of bias (eg. publication bias)	n/a
	#7b	Justification for exclusion (eg, exclusion of non–English-language citations)	n/a
	#7c	Assessment of quality of included studies	5
**Conclusion**			
	#8a	Consideration of alternative explanations for observed results	n/a
	#8b	Generalization of the conclusions (ie, appropriate for the data presented and within the domain of the literature review)	14

For peer review only

#8c	Guidelines for future research	15
#8d	Disclosure of funding source	23

None Reproduced with permission from JAMA. 2000. 283(15):2008-2012. Copyright © 2000 American Medical Association. All rights reserved.This checklist can be completed online using https://www.goodreports.org/, a tool made by the EQUATOR Network in collaboration with Penelope.ai

For peer review only
